# The role of an oxidized lithospheric mantle in gold mobilization

**DOI:** 10.1126/sciadv.ado6262

**Published:** 2024-10-11

**Authors:** Kun-Feng Qiu, Rolf L. Romer, Zheng-Yu Long, Anthony E. Williams-Jones, Hao-Cheng Yu, Simon Turner, Qing-Fei Wang, Shan-Shan Li, Jing-Yuan Zhang, Hao-Ran Duan, Jun Deng

**Affiliations:** ^1^ Frontiers Science Center for Deep-time Digital Earth, State Key Laboratory of Geological Processes and Mineral Resources, School of Earth Sciences and Resources, China University of Geosciences, Beijing, China.; ^2^ GFZ German Research Centre for Geosciences, Telegrafenberg, 14473 Potsdam, Germany.; ^3^ Department of Earth and Planetary Sciences, McGill University, Montreal H3A 0E8, Canada.; ^4^ Department of Earth and Planetary Sciences, Macquarie University, Sydney, Australia.; ^5^ Geological Research Institute of Shandong Gold Group Co., Ltd., Jinan 250013, China.

## Abstract

Phanerozoic orogenic gold mineralization at craton margins is related to the metasomatism of the lithospheric mantle by crustal material. Slab subduction transfers Au from the crust to the metasomatized mantle and oxidizes the latter to facilitate the mobilization of Au into mantle melts. The role of volatiles in the mobilization of Au in the mantle is unclear because of the absence of direct geochemical evidence relating the mantle source of Au to Au mineralization in the overlying crust. This study uses lithium isotopes from a large suite of lamprophyres to characterize the mantle beneath the eastern North China Craton, which hosts giant Mesozoic gold deposits. Our results indicate a strong genetic link between carbonate metasomatism in the mantle and Au mineralization in the overlying crust. Although pre-enrichment of Au in the mantle is critical for forming giant Au provinces, the oxidation of the lithospheric mantle controls the mobilization of Au.

## Teaser

Carbonate metasomatism links the mantle’s gold endowment to giant gold deposits in the crust.

## INTRODUCTION

The distribution of gold (Au) between the crust and mantle is uneven. Known Au resources are located within the continental crust (*1*–*3*). Most of this Au was stored in the crust of Archean cratons prior to 3.0 billion years (*1*, *4*, *5*), whereas the subcontinental lithospheric mantle (SCLM) beneath these ancient cratons is typically depleted in Au and volatiles because of earlier melt extraction (*6*). Nonetheless, at plate boundaries the SCLM is regarded as the source of gold for many Phanerozoic orogenic Au deposits (*7*–*10*). For the SCLM to become the source of Au, it has to be fertilized with crustal Au added via subduction (*6*, *9*, *11*). The subducted material not only generates a gold-enriched metasomatized SCLM but it also influences its oxidation state, which may affect the mobilization of Au during later events (*12*, *13*). The distribution of the chalcophile element Au in the mantle is controlled primarily by sulfides (*14*, *15*). Gold concentration is relatively low in magmas derived from a reduced mantle because Au is retained in residual sulfides or scavenged from the melt by sulfide melt. In contrast, Au and chalcophile elements are mobilized and extracted from the SCLM during partial melting (*16*) when the sulfide minerals are oxidized (*17*–*21*). The subduction of organic-rich continental sediments generally leads to a relatively reduced SCLM. However, fluids derived from subducted oceanic materials containing carbonates (C^4+^), sulfates (S^6+^), and ferric iron oxides (Fe^3+^) in the oxidized upper part of the subducted oceanic slab (*22*, *23*) are likely to oxidize the SCLM (*24*–*26*). For example, carbonate metasomatism leads to a relatively oxidized mantle (*25*), destabilizes Au-bearing sulfides and liberates Au and sulfur-rich fluids/melts (*18*, *27*, *28*). Moreover, CO_2_ can regulate pH, facilitating the transport of Au (*29*). The orogenic Au deposits form from auriferous aqueous-carbonic fluids (*30*, *31*), implying that the presence of carbon might be an inherent source characteristic. The role of volatiles in oxidizing the mantle and facilitating Au mobilization, however, remains unclear, largely due to the lack of geochemical evidence, such as that provided by isotopic and trace-element data, linking a mantle source of Au to Au mineralization in the overlying crust.

Calc-alkaline lamprophyres are important for assessing the role of mantle source preconditioning by crustal recycling because they closely mirror the characteristics of the subducted material. These rocks are the products of hydrous calc-alkaline magmas generated at low degrees of partial melting of the lithospheric mantle. Thus, they provide a geochemical record of the materials responsible for the enrichment and redox state of the mantle and the nature of fluid/melt-mantle interaction at the time of magma generation (*10*, *32*, *33*). Although the Au mineralization is typically not linked directly to such small-volume mantle-derived magmas, the temporal and spatial association of lamprophyres with major Au deposits and the high contents of Au recorded in these rocks (*33*–*35*) imply that lamprophyres sampled from the same metasomatized SCLM as the Au deposits. Thus, the chemical and isotopic composition of lamprophyres may open a window onto the mantle source for the Au deposits (*36*, *37*).

**Fig. 1.**
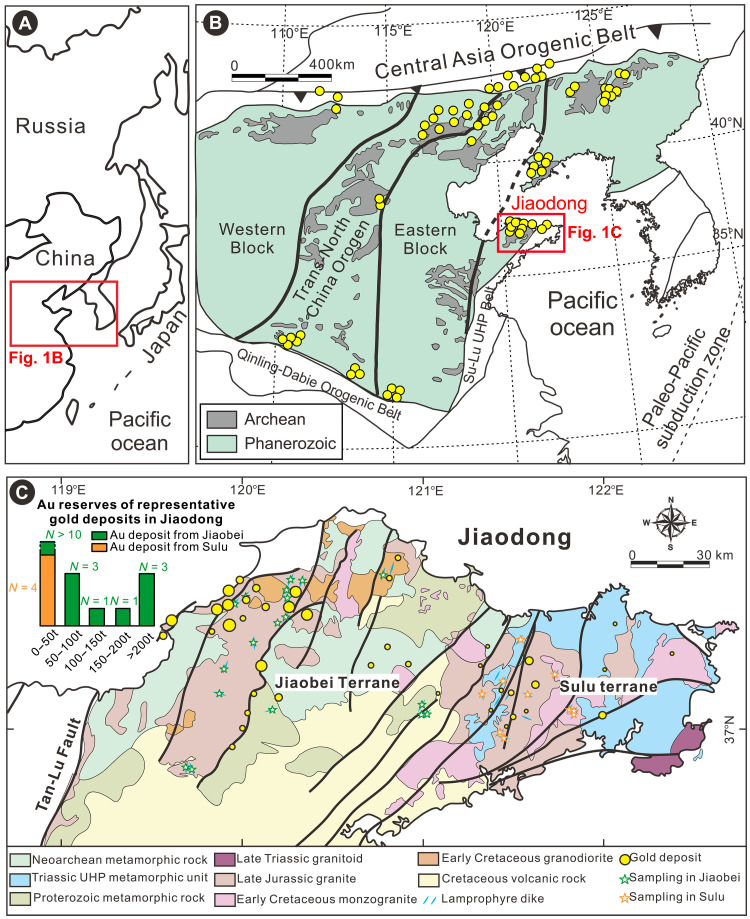
A simplified geological map. (**A**) The location of the North China Craton (NCC). (**B**) The location of the Jiaodong gold province in the NCC. The red rectangle is enlarged in panel (C). (**C**) The location of samples and major gold deposits in the Jiaodong province. The reserves of representative gold deposits in the Jiaodong province are listed in table S1.

The Jiaodong Au province, which is located in the eastern North China Craton (NCC) (Fig. 1), contained a premining resource of >5000 tonnes of Au (*38*). The source of the giant Au mineralization event at ~120 million years (Ma) at Jiaodong is considered to have been sourced from metasomatized SCLM (*33*, *35*, *39*). Early Cretaceous lamprophyres sampled the SCLM beneath the NCC, which was metasomatized during multiple subduction events (*40*–*43*). Subduction of continent-derived sediments and oceanic crust is interpreted to have led to Au enrichment of the SCLM (*30*, *44*–*46*) and caused the chemical conditions that allowed for later Au mobilization. As a result, this province provides an exceptionally good natural laboratory for investigating the genetic connection between metasomatized SCLM and orogenic Au mineralization at the margins of cratons. This study uses lithium (Li) isotopes from a large suite of lamprophyres, granitoids, and metamorphic rocks in the Jiaodong Au province to investigate the mantle metasomatic events that occurred beneath the eastern NCC. Our findings provide compelling evidence that oxidation of the Au-enriched lithospheric mantle played an essential role in the mobilization and recycling of gold into the overlying crust.

### Geological background

The NCC is composed of major Archean crustal blocks assembled in the early Paleoproterozoic during the formation of the Trans-North China Orogen (Fig. 1A) (*47*). These rocks experienced amphibolite- to granulite-facies metamorphism during the Paleoproterozoic that depleted the crustal rocks in Au and volatiles (*44*, *48*). Thus, they could not have been the source of Au mineralization. Metamorphic rocks of lower grade may have acted as sinks for the Au lost from the high-grade rocks during their Paleoproterozoic metamorphism. These lower-grade rocks were later eroded and reworked during the Proterozoic and late Paleozoic to Mesozoic subduction (*45*) and collisional events that eventually led to the assembly of Asia. As a result, Au and volatiles were transported into the SCLM (*33*, *41*, *45*) that subsequently served as the source reservoir for the Cretaceous Au mineralization (Fig. 1). For further detail, see the Supplementary Materials.

Slab subduction in the Jurassic and rollback of the Paleo-Pacific plate in the Early Cretaceous resulted in extensive lithospheric extension in the Jiaodong Au province that was accompanied by felsic (e.g., Jurassic Linglong granites and Early Cretaceous Guojialing granitoids) to mafic magmatism (e.g., Early Cretaceous lamprophyres) and Au mineralization (*31*, *42*, *49*). It is noteworthy that Early Cretaceous lamprophyre dikes are spatially and temporally related to the Au mineralization (*33*, *50*). They have relatively heavy sulfur isotope compositions [δ^34^S = +4 to +6 per mil (‰)] and mass-dependent mercury isotope signatures (Δ^199^Hg = −0.1 to +0.1‰), similar to those of the gold-bearing pyrite and native gold in the ore deposits (*39*, *51*), suggesting that lamprophyric magmas share a common mantle source with the parental melts/fluids that evolved to the auriferous fluids.

More than 85% of the Au resource in the Jiaodong province is hosted in the Jiaobei Terrane in the western part of the Jiaodong Au province (*38*) (Fig. 1B), whereas the remainder is in the Sulu Terrane in eastern Jiaodong. Both the Jiaobei and Sulu terranes share a common post-Triassic tectonic history (*35*, *38*). The reason for the heterogeneous distribution has not been well established (*52*). The heterogeneity may be related to regional variations in the Au endowment or the redox state of the metasomatized SCLM; a more oxidized SCLM would have released its gold to fluids or magmas, whereas a reduced mantle would have retained more Au in sulfides (*42*).

## RESULTS

**Fig. 2.**
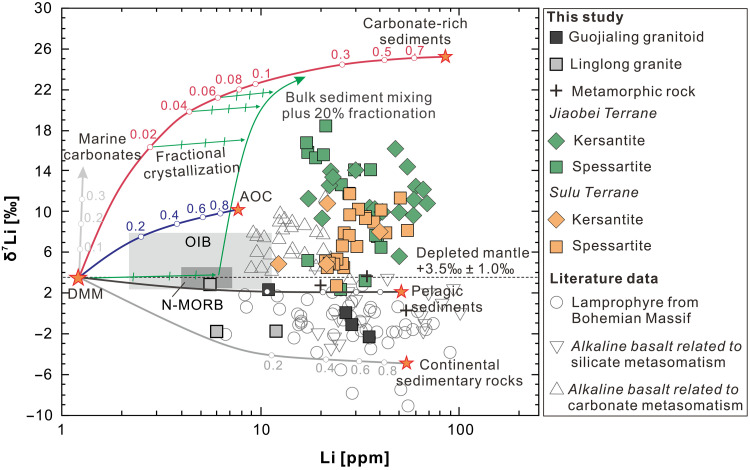
A plot of δ^7^Li versus Li content of granitoids, basalts, metamorphic rock, and lamprophyres from Jiaodong, the Bohemian Massif, and West Qinling. Also shown are δ^7^Li versus Li mixing curves between the depleted MORB mantle (DMM) end-member and various crustal reservoirs. The numbers on the curves indicate the mass fraction of assimilated material. The subhorizontal green arrows, with tick marks at 5% intervals, indicate how fractional crystallization affects the magma composition. The green curve shows the effect of combined assimilation and fractional crystallization at equilibrium isotope fractionation. A more detailed description of the modeling is provided in the Supplementary Materials.

Lamprophyre dikes from the Jiaodong Au province exhibit a wide range of δ^7^Li values (from +2.4 to +18.4‰; *n* = 59) and Li concentrations [12.4 to 68.9 parts per million (ppm)]. Samples from the Jiaobei Terrane (+2.4 to +18.4‰; *n* = 34) and the Sulu Terrane (+2.8 to +11.4‰; *n* = 25) have an overlapping range, although those from the Jiaobei Terrane generally have the higher δ^7^Li values. Kersantites and spessartites, the two lamprophyre subtypes found in Jiaodong (their mineral compositions are reported in the Supplementary Materials), have similar Li concentrations and isotope compositions (Fig. 2). The δ^7^Li values of the lamprophyres are notably higher than those of Precambrian metamorphic rocks (amphibolite and gneiss; −2.3 to +2.4‰; *n* = 4), Jurassic Linglong-type granites (−1.8 to +2.9‰; n = 3), and the Early Cretaceous Guojialing-type granitoids (−1.1 to +3.6‰; *n* = 4), as well as normal peridotitic mantle, ocean island basalt (OIB), and mid-ocean ridge basalt (MORB) (δ^7^Li = 3.5 ± 1‰; Fig. 2) (*53*). Because the observed range of δ^7^Li values cannot be explained by the differentiation of magmas derived from the mantle, crustal assimilation, or surface alteration (see detailed discussion in the Supplementary Materials), the high δ^7^Li values of the Jiaodong lamprophyres are interpreted to reflect their SCLM source.

## DISCUSSION

### Gold introduction into the SCLM

The lithospheric mantle beneath Archean cratons has experienced high degrees of partial melting and, therefore, it is depleted in Au that had partitioned into mantle-derived magmas before and during cratonization (*6*). Depleted SCLM has a low Au content, whereas metasomatized SCLM has a markedly higher Au content (*54*). Early Cretaceous lamprophyres [up to 3.6 parts per billion (ppb)] from Jiaodong and basalts (up to 4.3 ppb) from the NCC have similarly elevated Au contents (*33*, *55*) relative to the depleted mantle (<0.5 ppb) (*33*) and primitive mantle (~0.88 ppb) (*56*). Globally, peridotites, in particular those that have been metasomatized, have Au contents of ~1.2 ppb (*57*) and locally carry nanometric-size Au minerals or Au-bearing inclusions (*58*). To summarize, the SCLM can only be a source of substantial amounts of Au, if subduction-related metasomatism has replenished its Au endowment (*59*, *60*).

Gold returned to the mantle by subduction may have multiple sources. The oceanic crust, with the possible exception of oceanic plateaus (*61*), likely contributes very little to the Au budget of the mantle because of its relatively low Au content. Gold mineralization occurs in magmatic arcs and back-arc basins at continental margins, whereas intra-oceanic arcs do not host such mineralization. This suggests that Au is recycled into the mantle from the terrigenous sedimentary cover overlying the subducting slab (*2*), where it would be concentrated in diagenetic pyrite. As most of the Au is stored within the present continental crust (*1*, *5*, *62*), the distribution of post-Archean Au deposits, in particular Phanerozoic orogenic Au deposits, must reflect the recycling of Au from older continental crust (*1*, *2*, *10*). Subduction of sedimentary rocks or terrigenous sediments deposited at continental margins fertilizes the SCLM and makes Au available for later mobilization via melting of the SCLM.

**Fig. 3.**
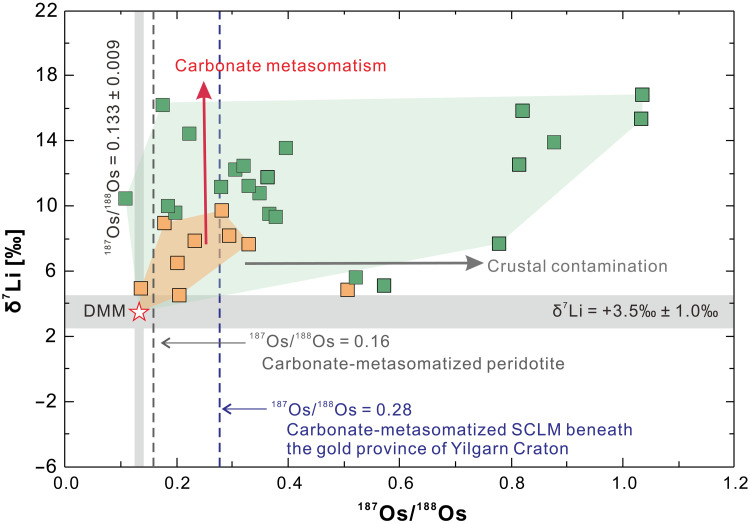
A plot of δ^7^Li versus ^187^Os/^188^Os. Showing the values of lamprophyres from the Jiaobei (green squares) and Sulu (orange squares) terranes. Also shown is the variation of δ^7^Li and age-corrected ^187^Os/^188^Os values of the depleted mantle due to the addition of crustal material (silicate metasomatism) and carbonates. The grey bands show the δ^7^Li and age-corrected ^187^Os/^188^Os values, respectively, of the depleted mantle. The gray and blue dashed lines show the calculated ^187^Os/^188^Os ratios for carbonate metasomatized peridotite mantle (*63*) and metasomatized SCLM (*79*), respectively. Assimilation of material from the continental crust may cause a substantial increase in the ^187^Os/^188^Os values of the mantle melts (*75*) but this does not result in a substantial increase in δ^7^Li values. The modeling is described in the Supplementary Materials.

**Fig. 4.**
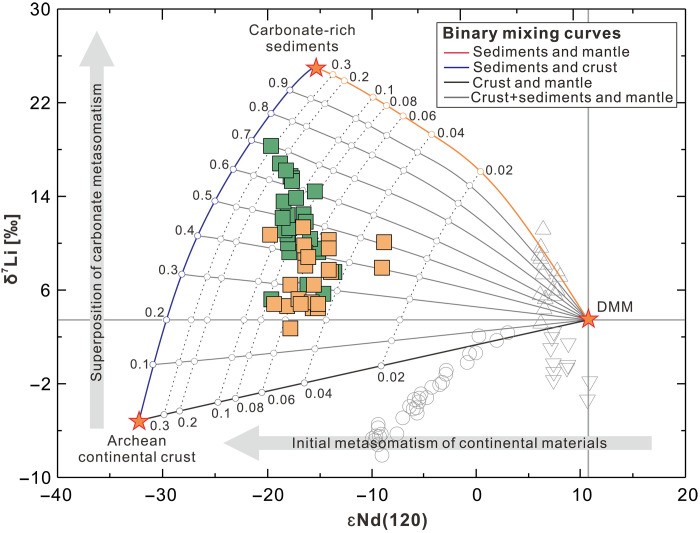
A binary plot of δ^7^Li versus ε_Nd(120)_ showing the variation of δ^7^Li and ε_Nd(120)_ in the three-component system mantle, continental crust, and carbonate-rich sediments. The compositions of the Jiaobei lamprophyres are indicated by the green squares and those of the Sulu lamprophyres by the orange squares. The open symbols are as in Fig. 2. The higher ε_Nd_ values of the Bohemian Massif lamprophyres (open circles) and the alkali basalts (open triangles) reflect the younger average age of the subducted crustal component. For details see the Supplementary Materials.

**Fig. 5.**
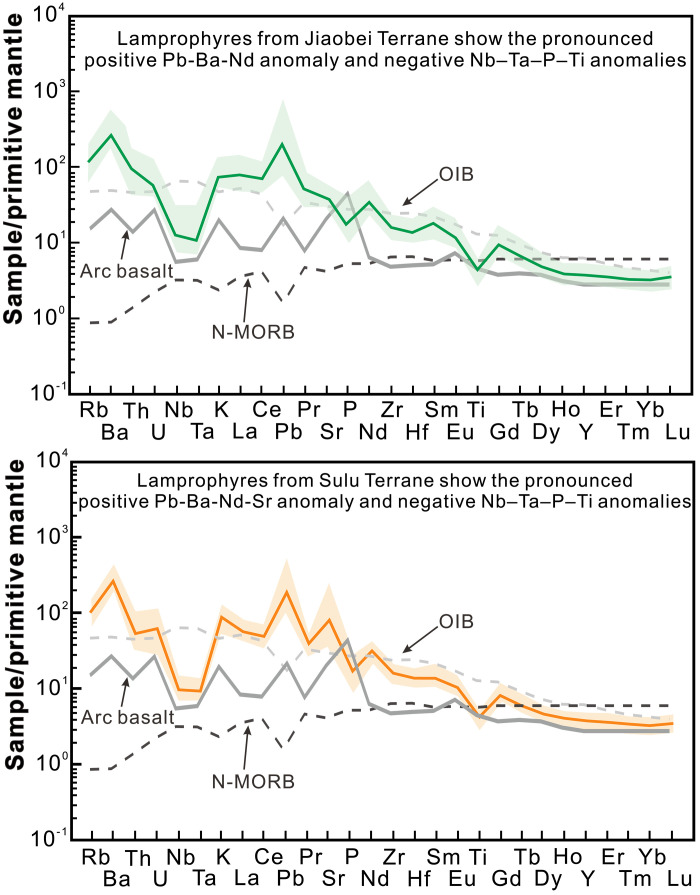
Plots illustrating trace element abundances of lamprophyres. The trace element values are normalized to those of the primitive mantle. All the lamprophyres display negative Ti-Nb-Ta and P anomalies and positive Pb, Ba, and Nd anomalies. Note the positive Sr anomaly for lamprophyres from the Sulu Terrane. The lamprophyre data are from (*35*). The primitive mantle values are from (*83*).

The lamprophyres considered in this study exhibit high Au/Cu_(*N*)_ and Au/Pd_(*N*)_ ratios, as well as high Ba/Nb ratios, indicating a discernible Au addition to their source via subduction-related enrichment (*33*). These rocks have other geochemical signatures typical of a mantle affected by metasomatism from continental material, including high ^87^Sr/^86^Sr_(120)_ (fig. S1) and ^187^Os/^188^Os_(120)_ ratios (*32*, *63*) (Fig. 3) and low ε_Nd(120)_ values (Fig. 4), as well as an enrichment in incompatible elements such as Ba, Pb, Sr, and rare earth element and a relative depletion in Ti, Nb, and Ta (Fig. 5). However, these lamprophyres have higher δ^7^Li values (+2.4 to +18.4‰) than average upper continental crust (+0.6 ± 0.6‰) (*64*), sediments (*65*), and weathering products derived from continental crust and metamorphic rocks (as low as −20‰) (*66*). Metasomatic domains in the SCLM typically have δ^7^Li values ranging between those of depleted MORB mantle (+3.5 ± 1.0‰) (*53*) and those of subducted continental crustal rocks (*67*–*69*). For example, lamprophyres from the SCLM beneath the Bohemian Massif, which was modified by the subduction of continental material, have elevated ^87^Sr/^86^Sr ratios, low ^143^Nd/^144^Nd ratios, and low δ^7^Li values (Fig. 2) (*32*). The subduction of sediment may thus account for the Au enrichment and the crustal trace element and Sr-Nd-Os isotope signatures of the Jiaodong SCLM but not the unusually high δ^7^Li values that point to a SCLM modified by processes in addition to the addition of continental material. These processes erased the crustal signature of Li but did not affect the crustal Sr, Os, and Nd isotope and trace element signatures.

### Oxidation of the SCLM

**Fig. 6.**
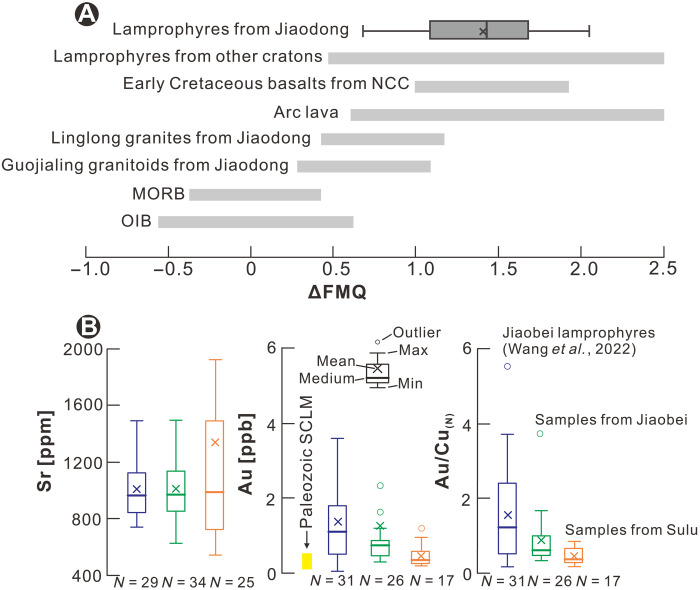
Plots of oxygen fugacity, Au and Sr contents, and Au/Cu_(***N***)_ ratios of lamprophyres. (**A**) The oxygen fugacity (relative to the quartz-fayalite-magnetite buffer, ΔQFM) of different types of rocks. The data for the lamprophyres of the NCC are presented in table S7, the Jiaodong and Guojialing granitoids are from (*70*), the Early Cretaceous basalts from the NCC are from (*43*) and (*84*), and other rock data are from (*84*) and references therein. (**B**) Box and whisker plots of the Sr and Au contents and Au/Cu_(*N*)_ ratios of lamprophyres from the Jiaodong gold province. The data for the Jiaobei and Sulu lamprophyre samples are reported in table S2. Additional data for the Jiaobei lamprophyres are from (*33*). The gold values for the Paleozoic SCLM beneath the eastern NCC (<0.5 ppb) are from (*55*). The primitive mantle values are from (*83*).

Once introduced into the mantle, Au remains immobile until the metasomatized domain is oxidized and Au is released from its sulfide hosts (*18*, *19*). The oxidation state of the SCLM depends on the nature of the subducted material and would be reduced, if the subducted sedimentary material was rich in organic material (*12*, *13*), ensuring that any sulfur would occur as sulfide and sequester Au. There were two events of Early Cretaceous SCLM-related magmatism in the Jiaodong Au province, specifically the granitic magmatism that preceded Au mineralization and the lamprophyric magmatism that was coeval with it. In the first event, partial melting generated melts characterized by the chemical signature of subducted continental material with low δ^7^Li values (−1.1 to +3.6‰, e.g., the Guojialing granitoid at ~130 to 122 Ma; Fig. 2) but without anomalous Au (0.031 to 0.047 ppb) (*70*). The low δ^7^Li values of these felsic rocks contrast with the high δ^7^Li values of the younger lamprophyres (+2.4 to +18.4‰; Fig. 2), indicating either that the lamprophyres had a different mantle source or that their mantle source was modified by plate rollback in the period between the extraction of the granitic and lamprophyric magmas. The Jiaodong lamprophyres have *f*O_2_ values (ΔFMQ of +1 to +2) higher than those of MORB, OIB, and earlier granitoids from the Jiaodong province, but similar to those of Early Cretaceous basalts from the NCC, and arc lavas and lamprophyres from other cratons (Fig. 6A), reflecting a highly oxidized mantle source with enhanced Au contents (Fig. 6B) (*33*).

Subduction of oceanic crust typically leads to oxidation of the SCLM (*24*) and may add Li with elevated δ^7^Li (*53*). Seawater is characterized by high δ^7^Li (up to +31‰). Thus, rocks that have interacted extensively with seawater, such as altered oceanic crust (AOC), and minerals that precipitated from seawater, such as marine carbonates (up to 25‰) and authigenic minerals (clay minerals, glauconite, chlorite, and zeolites) (*53*), generally have elevated δ^7^Li values (*53*). Contributions from AOC (up to +10‰), however, cannot account for the unusually high δ^7^Li values (up to +18‰) recorded in the lamprophyres (Fig. 2). Moreover, additions from AOC would cause high ^143^Nd/^144^Nd ratios (Fig. 4). Although subducted carbonate minerals may be an important source of CO_2_, Sr, and Ca, they would have had little influence on the δ^7^Li value of the SCLM because they have very low Li contents (as shown by the modeled mixing trend between mantle and carbonate in Fig. 2). In contrast, authigenic clay minerals and glauconite have high Li contents and δ^7^Li values. High contents of these minerals in carbonate-rich sediments provide a CO_2_-rich source for mantle metasomatism that is characterized by high Li, Ca, and Sr contents and high δ^7^Li values (Fig. 2) (*71*, *72*).

The subduction of carbonate-rich sediments transfers heavy δ^7^Li into the overlying mantle wedge. Carbonate melts produced from such sediment, because of their very low viscosity, have the potential to infiltrate the mantle, and promote extensive metasomatism (*73*). Evidence for carbonate metasomatism is provided by the heavy Mg isotope compositions of the Jiaodong lamprophyres, which are interpreted to reflect the recycling of carbonates in the mantle (fig. S2) (*74*). Moreover, the low Hf/Sm and elevated (La/Yb)*_N_* and Nb/Ta ratios of these lamprophyres are consistent with carbonate metasomatism (figs. S3 and S4) (*75*, *76*). Carbonate metasomatism will barely affect the ^43^Nd/^144^Nd and ^187^Os/^188^Os ratios of the mantle, as demonstrated by Cenozoic alkaline basalts that have experienced carbonate metasomatism (Fig. 4) (*76*). Last, remelting of the relatively reduced SCLM during the Jurassic could have resulted in the removal of considerable material but with limited mobilization of Au as supported by low δ^7^Li signatures of intrusive rocks. As a result, the residual segments of the SCLM with lower Li contents would be more susceptible to modification by subsequent additions of high δ^7^Li materials. Lamprophyric melts derived from remelting of such a metasomatized mantle would display a mixed trace-element and isotope signature with contributions from both mantle and crustal reservoirs (*68*, *77*, *78*), as well as high Au contents (up to 3 ppb) (*33*).

### Gold enrichment in the source and mantle oxidization

The Jiaobei lamprophyres have higher δ^7^Li values (Fig. 4) and lower Sr contents, on average than those from Sulu (Fig. 5). However, there is a much greater variation in the Sr contents of the Sulu samples, ranging from notably lower to considerably higher than the Sr content of the Jiaobei samples (Fig. 6B). The Sulu lamprophyres with higher Sr contents could reflect a greater abundance of recycled carbonates in the underlying SCLM than the Jiaobei lamprophyres. This, in turn, would imply a more oxidized mantle domain beneath the Sulu Terrane. If the introduction of CO_2_ into the mantle and the resulting oxidation of the latter were the only factors determining the availability of Au in the mantle, the Sr content of the lamprophyres would predict that the Sulu Terrane should show a greater Au endowment than the Jiaobei Terrane. The opposite is observed. Most of the deposits in the Jiaodong gold province are located in the Jiaobei Terrane, which has an order of magnitude larger Au endowment than the Sulu Terrane (Fig. 1B) (*38*). Moreover, the lamprophyres from the Jiaobei Terrane have higher Au contents and Au/Cu_(*N*)_ ratios than the lamprophyres from the Sulu Terrane (Fig. 6B), implying a greater availability of Au in the underlying mantle (*33*–*35*). As the Sr contents suggest that the Jiaobei mantle was less modified by the subduction of carbonates and, thus, less oxidized than the Sulu mantle, it follows that the Jiaobei mantle was more enriched in Au. A higher enrichment would compensate for the Au retained in sulfides and would facilitate the release of a sufficient quantity of Au to produce the resources in the Jiaodong province. In summary, although oxidation of the SCLM was essential for the mobilization of Au, it was the relative degree of enrichment of the SCLM via the subduction of crustal material that was responsible for the Jiaobei Terrane having a much larger Au resource than the Sulu Terrane.

Continental crust and the mantle have very different Re/Os ratios and, therefore, crustal and mantle materials have very different Os isotope compositions. Over time, crustal rocks develop high ^187^Os/^188^Os values. As Os and Au are highly siderophile and chalcophile elements that are likely to behave similarly in the mantle, the Os isotope composition of lamprophyres can readily identify crustal sources of Au recycled into the mantle (*75*, *79*). High ^187^Os/^188^Os values in lamprophyres provide evidence that there was a large input of crustal material, which overwhelmed the mantle signature, or that the mantle had a low Os content before becoming metasomatized by crustal material. Regions with low Os contents acquire higher ^187^Os/^188^Os ratios through minor additions of recycled material during mantle metasomatism. Conversely, small contributions of crustal material imply a small contribution of Au. In either case, the higher ^187^Os/^188^Os ratios in the Jiaobei lamprophyres compared to the Sulu lamprophyres imply differences in mantle enrichment in the two districts. A possible reason for this variation is the environment in which the subducted sediments originated: a shelf setting proximal to an Au-enriched hinterland and a distal shelf setting deprived of Au (*2*, *45*). Differences in the sources of the subducted sediments may also have arisen from the juxtaposition of the Jiaobei and Sulu terranes along the Tanlu fault (*38*). In either case, the sediments that were recycled into the SCLM beneath the Jiaobei Terrane may have had a larger proportion of shale (containing clay minerals and Au-rich pyrite) and pore water with higher-δ^7^Li than the sediments subducted beneath the Sulu Terrane, which would have had a high proportion of carbonate minerals. Such an interpretation is supported by the fact that the Jiaobei lamprophyres (with some overlap) exhibit higher ^87^Sr/^86^Sr_i_ ratios (0.7088 to 0.7110) than the Sulu lamprophyres (0.7079 to 0.7098; fig. S1). This is because detrital clay minerals from old crustal sources inherit the high ^87^Sr/^86^Sr ratios of their precursor minerals and have high^87^Rb contents, the decay of which with time further increases the ^87^Sr/^86^Sr ratios. Carbonates and marine authigenic clay minerals, however, inherit the ^87^Sr/^86^Sr ratios of seawater that for the Phanerozoic is less than 0.7092 (*80*).

Subduction-related enrichment of the SCLM can recycle substantial amounts of Au and volatiles into the mantle that subsequently return to the overriding continental crust. It is important to note, however, that partial melting of relatively reduced mantle would generate melts carrying a crustal signature, without mobilizing chalcophile and siderophile elements (such as Au and Os) so long as sulfide minerals remain stable. Oxidation of the mantle is required to permit mobilization of these metals into melts and/or fluids that ascend into the crust and an effective way to do this is via the subduction of oceanic crust and carbonate-rich sedimentary rocks. Consequently, Au cycling at continental margins is not restricted to the consumption of ancient continental crust or sediments from this crust for Au extraction but also necessitates the acquisition of volatile elements from oceanic sediments to oxidize the lithospheric mantle.

In summary, volatiles from the metasomatized SCLM can effectively release and transport Au from its mantle source but the magnitude of this enrichment in fluid or melt is limited, if the source possesses an inherently low Au content. The essential preconditions for the formation of Au provinces are a metasomatized lithospheric mantle that is enriched in Au and is sufficiently oxidized to permit the mobilization of the Au into the overlying crust.

## MATERIALS AND METHODS

This study is based on samples collected from the main Au districts of the Jiaobei and Sulu terranes in the Jiaodong Au province. The samples are free of any evidence of hydrothermal alteration and/or supergene weathering. A detailed description of these samples, including their mineralogy, petrography, and major and trace element and Sr-Nd-Pb isotope composition, is provided in Supplementary Materials.

The Li isotope compositions presented in this study were determined at the Institute of Geology, Chinese Academy of Geological Sciences, Beijing, China using a Nu Plasma II multicollector inductively coupled plasma mass spectrometer. Sample loss during the chemical separation results in Li isotopic fractionation. To exclude this possibility, we collected pre- and post-Li eluate fractions for all samples and verified the quality of the separation procedure by mass balance. There was generally less than 0.1% Li loss during the ion-exchange procedure. For details on the sample treatment and analytical procedures, see (*81*). Isotope ratios were measured using the standard-sample-standard bracketing method and international reference material L-SVEC as the Li isotope standard (*82*). To ensure the precision and reproducibility of the chemical separation and analysis, standard reference materials (BCR-2 and GSP-2) were processed with each set of 10 unknown samples. The Li isotope compositions of the two reference materials agree with the published values (table S1), confirming that our data are accurate. The long-term external precision of δ^7^Li is better than ±0.47‰ (2SD) (*81*). Lithium isotope fractionation is expressed relative to the L-SVEC standard using the δ^7^Li (‰) notation: δ^7^Li (‰) = [(^7^Li^/6^Li _sample_ / ^7^Li^/6^Li _standard_) − 1] × 1000. The chemical and isotope data for the samples are provided in table S2.

## Supplementary Materials

### This PDF file includes:

Supplementary Text Figs. S1 to S5 Tables S1 to S7 References

## References

[1] H. E. Frimmel, Earth’s continental crustal gold endowment. Earth Planet. Sci. Lett. 267, 45–55 (2008).

[2] R. L. Romer, U. Kroner, Paleozoic gold in the Appalachians and Variscides. Ore Geol. Rev. 92, 475–505 (2018).

[3] R. J. Goldfarb, C. Hart, G. Davis, D. Groves, East Asian gold: Deciphering the anomaly of Phanerozoic gold in Precambrian cratons. Econ. Geol. 102, 341–345 (2007).

[4] D. I. Groves, R. M. Vielreicher, R. J. Goldfarb, K. C. Condie, Controls on the heterogeneous distribution of mineral deposits through time. Geol. Soc. Lond. Spec. Publ. 248, 71–101 (2005).

[5] R. J. Goldfarb, D. I. Groves, S. Gardoll, Orogenic gold and geologic time: A global synthesis. Ore Geol. Rev. 18, 1–75 (2001).

[6] W. L. Griffin, S. Y. O’Reilly, J. C. Afonso, G. C. Begg, The composition and evolution of lithospheric mantle: A re-evaluation and its tectonic implications. J. Petrol. 50, 1185–1204 (2009).

[7] W. L. Griffin, G. C. Begg, S. Y. O’Reilly, Continental-root control on the genesis of magmatic ore deposits. Nat. Geosci. 6, 905–910 (2013).

[8] S. Tassara, A. D. Rooney, J. J. Ague, D. Guido, M. Reich, F. Barra, C. Navarrete, Osmium isotopes fingerprint mantle controls on the genesis of an epithermal gold province. Geology 50, 1291–1295 (2022).

[9] D. I. Groves, L. Zhang, M. Santosh, Subduction, mantle metasomatism, and gold: A dynamic and genetic conjunction. GSA Bull. 132, 1419–1426 (2020).

[10] C. G. Soder, J. Dunga, R. L. Romer, Continental subduction controls regional magma heterogeneity and distribution of porphyry deposits in post-collisional settings. Geochim. Cosmochim. Acta 375, 217–228 (2024).

[11] R. Zhu, W. Sun, The big mantle wedge and decratonic gold deposits. Sci. China Earth Sci. 64, 1451–1462 (2021).

[12] S. Ishimaru, S. Arai, H. Shukuno, Metal-saturated peridotite in the mantle wedge inferred from metal-bearing peridotite xenoliths from Avacha volcano, Kamchatka. Earth Planet. Sci. Lett. 284, 352–360 (2009).

[13] J. Wang, K. H. Hattori, R. Kilian, C. R. Stern, Metasomatism of sub-arc mantle peridotites below southernmost South America: Reduction of *f*O_2_ by slab-melt. Contrib. Mineral. Petrol. 153, 607–624 (2006).

[14] F. E. Jenner, Cumulate causes for the low contents of sulfide-loving elements in the continental crust. Nat. Geosci. 10, 524–529 (2017).

[15] W. Sun, R. J. Arculus, V. S. Kamenetsky, R. A. Binns, Release of gold-bearing fluids in convergent margin magmas prompted by magnetite crystallization. Nature 431, 975–978 (2004). 15496920 10.1038/nature02972

[16] J. E. Mungall, J. J. Hanley, N. T. Arndt, A. Debecdelievre, Evidence from meimechites and other low-degree mantle melts for redox controls on mantle-crust fractionation of platinum-group elements. Proc. Natl. Acad. Sci. U.S.A. 103, 12695–12700 (2006). 16908861 10.1073/pnas.0600878103 PMC1568912

[17] J. E. Mungall, Roasting the mantle: Slab melting and the genesis of major Au and Au-rich Cu deposits. Geology 30, 915–918 (2002).

[18] R. E. Botcharnikov, R. L. Linnen, M. Wilke, F. Holtz, P. J. Jugo, J. Berndt, High gold concentrations in sulphide-bearing magma under oxidizing conditions. Nat. Geosci. 4, 112–115 (2011).

[19] Y. Li, L. Feng, E. S. Kiseeva, Z. Gao, H. Guo, Z. Du, F. Wang, L. Shi, An essential role for sulfur in sulfide-silicate melt partitioning of gold and magmatic gold transport at subduction settings. Earth Planet. Sci. Lett. 528, 115850 (2019).

[20] C. Grondahl, Z. Zajacz, Magmatic controls on the genesis of porphyry Cu–Mo–Au deposits: The Bingham Canyon example. Earth Planet. Sci. Lett. 480, 53–65 (2017).

[21] C. Zhang, W. Sun, J. Wang, L. Zhang, S. Sun, K. Wu, Oxygen fugacity and porphyry mineralization: A zircon perspective of Dexing porphyry Cu deposit, China. Geochim. Cosmochim. Acta 206, 343–363 (2017).

[22] J. C. Alt, Sulfur isotopic profile through the oceanic crust: Sulfur mobility and seawater-crustal sulfur exchange during hydrothermal alteration. Geology 23, 585–588 (1995).

[23] J. C. Alt, E. M. Schwarzenbach, G. L. Früh-Green, W. C. Shanks, S. M. Bernasconi, C. J. Garrido, L. Crispini, L. Gaggero, J. A. Padrón-Navarta, C. Marchesi, The role of serpentinites in cycling of carbon and sulfur: Seafloor serpentinization and subduction metamorphism. Lithos 178, 40–54 (2013).

[24] K. A. Evans, The redox budget of subduction zones. Earth Sci. Rev. 113, 11–32 (2012).

[25] J. A. Padrón-Navarta, V. López Sánchez-Vizcaíno, M. D. Menzel, M. T. Gómez-Pugnaire, C. J. Garrido, Mantle wedge oxidation from deserpentinization modulated by sediment-derived fluids. Nat. Geosci. 16, 268–275 (2023).

[26] W. Li, Z. Yang, M. Chiaradia, Y. Lai, C. Yu, J. Zhang, Redox state of southern Tibetan upper mantle and ultrapotassic magmas. Geology 48, 733–736 (2020).

[27] G. S. Pokrovski, M. A. Kokh, D. Guillaume, A. Y. Borisova, P. Gisquet, J.-L. Hazemann, E. Lahera, W. Del Net, O. Proux, D. Testemale, V. Haigis, R. Jonchière, A. P. Seitsonen, G. Ferlat, R. Vuilleumier, A. M. Saitta, M.-C. Boiron, J. Dubessy, Sulfur radical species form gold deposits on Earth. Proc. Natl. Acad. Sci. U.S.A. 112, 13484–13489 (2015). 26460040 10.1073/pnas.1506378112 PMC4640777

[28] D. E. Blanks, D. A. Holwell, M. L. Fiorentini, M. Moroni, A. Giuliani, S. Tassara, J. M. González-Jiménez, A. J. Boyce, E. Ferrari, Fluxing of mantle carbon as a physical agent for metallogenic fertilization of the crust. Nat. Commun. 11, 4342 (2020). 32859892 10.1038/s41467-020-18157-6 PMC7455710

[29] G. N. Phillips, K. A. Evans, Role of CO_2_ in the formation of gold deposits. Nature 429, 860–863 (2004). 15215861 10.1038/nature02644

[30] R. J. Goldfarb, D. I. Groves, Orogenic gold: Common or evolving fluid and metal sources through time. Lithos 233, 2–26 (2015).

[31] R. J. Goldfarb, R. D. Taylor, G. S. Collins, N. A. Goryachev, O. F. Orlandini, Phanerozoic continental growth and gold metallogeny of Asia. Gondw. Res. 25, 48–102 (2014).

[32] L. Krmíček, R. L. Romer, M. J. Timmerman, J. Ulrych, J. Glodny, A. Přichystal, M. Sudo, Long-lasting (65 Ma) regionally contrasting late- to post-orogenic Variscan mantle-derived potassic magmatism in the Bohemian Massif. J. Petrol. 61, egaa072 (2020).

[33] X. Wang, Z. Wang, H. Cheng, K. Zong, C. Y. Wang, L. Ma, Y.-C. Cai, S. Foley, Z. Hu, Gold endowment of the metasomatized lithospheric mantle for giant gold deposits: Insights from lamprophyre dykes. Geochim. Cosmochim. Acta 316, 21–40 (2022).

[34] E. Choi, M. L. Fiorentini, H. S. R. Hughes, A. Giuliani, Platinum-group element and Au geochemistry of late Archean to Proterozoic calc-alkaline and alkaline magmas in the Yilgarn Craton, Western Australia. Lithos 374–375, 105716 (2020).

[35] J. Deng, X. Liu, Q. Wang, Y. Dilek, Y. Liang, Isotopic characterization and petrogenetic modeling of early Cretaceous mafic diking—Lithospheric extension in the North China Craton, Eastern Asia. GSA Bull. 129, 1379–1407 (2017).

[36] N. M. Rock, D. I. Groves, Do lamprophyres carry gold as well as diamonds? Nature 332, 253–255 (1988).

[37] D. Müller, D. I. Groves, Indirect associations between lamprophyres and gold-copper deposits in *Potassic Igneous Rocks and Associated Gold-Copper Mineralization* (Springer Cham, 2019), pp. 279–306.

[38] K.-F. Qiu, R. J. Goldfarb, J. Deng, H.-C. Yu, Z.-Y. Gou, Z.-J. Ding, Z.-K. Wang, D.-P. Li, Gold deposits of the Jiaodong Peninsula, Eastern China, in *Geology of the World’s Major Gold Deposits and Provinces*, R. H. Sillitoe, R. J. Goldfarb, F. Robert, S. F. Simmons, Eds. (Society of Economic Geologists, 2020), vol. 23, pp. 753–773; https://doi.org/10.5382/SP.23.35.

[39] J.-Y. Zhang, K.-F. Qiu, R. Yin, Z.-Y. Long, Y.-C. Feng, H.-C. Yu, Z.-Y. Gao, J. Deng, Lithospheric mantle as a metal storage reservoir for orogenic gold deposits in active continental margins: Evidence from Hg isotopes. Geology 52, 423–428 (2024).

[40] X. Geng, Y. Liu, X.-C. Wang, Z. Hu, L. Zhou, S. Gao, The role of Earth’s deep volatile cycling in the generation of intracontinental high-Mg andesites: Implication for lithospheric thinning beneath the North China Craton. J. Geophys. Res. Solid Earth 124, 1305–1323 (2019).

[41] Q.-K. Xia, J. Liu, S.-C. Liu, I. Kovacs, M. Feng, L. Dang, High water content in Mesozoic primitive basalts of the North China Craton and implications on the destruction of cratonic mantle lithosphere. Earth Planet. Sci. Lett. 361, 85–97 (2013).

[42] J. Deng, L.-Q. Yang, D. I. Groves, L. Zhang, K.-F. Qiu, Q.-F. Wang, An integrated mineral system model for the gold deposits of the giant Jiaodong province, Eastern China. Earth Sci. Rev. 208, 103274 (2020).

[43] X. Geng, S. F. Foley, Y. Liu, Z. Wang, Z. Hu, L. Zhou, Thermal-chemical conditions of the North China Mesozoic lithospheric mantle and implication for the lithospheric thinning of cratons. Earth Planet. Sci. Lett. 516, 1–11 (2019).

[44] R. J. Goldfarb, M. Santosh, The dilemma of the Jiaodong gold deposits: Are they unique? Geosci. Front. 5, 139–153 (2014).

[45] K.-F. Qiu, J. Deng, C. Laflamme, Z.-Y. Long, R.-Q. Wan, F. Moynier, H.-C. Yu, J.-Y. Zhang, Z.-J. Ding, R. Goldfarb, Giant Mesozoic gold ores derived from subducted oceanic slab and overlying sediments. Geochim. Cosmochim. Acta 343, 133–141 (2023).

[46] Q. Wang, X. Liu, R. Yin, W. Weng, H. Zhao, L. Yang, D. Zhai, D. Li, Y. Ma, D. I. Groves, J. Deng, Metasomatized mantle sources for orogenic gold deposits hosted in high-grade metamorphic rocks: Evidence from Hg isotopes. Geology 52, 115–119 (2023).

[47] T. Kusky, B. F. Windley, M.-G. Zhai, Tectonic evolution of the North China Block: From orogen to craton to orogen. Geol. Soc. Lond. Spec. Publ. 280, 1–34 (2007).

[48] Z. Wang, Z. Xu, H. Cheng, Y. Zou, J. Guo, Y. Liu, J. Yang, K. Zong, L. Xiong, Z. Hu, Precambrian metamorphic crustal basement cannot provide much gold to form giant gold deposits in the Jiaodong Peninsula, China. Precambrian Res. 354, 106045 (2021).

[49] J.-H. Yang, F.-Y. Wu, S. A. Wilde, E. Belousova, W. L. Griffin, Mesozoic decratonization of the North China block. Geology 36, 467–470 (2008).

[50] L. Ma, S.-Y. Jiang, A. W. Hofmann, B.-Z. Dai, M.-L. Hou, K.-D. Zhao, L.-H. Chen, J.-W. Li, Y.-H. Jiang, Lithospheric and asthenospheric sources of lamprophyres in the Jiaodong Peninsula: A consequence of rapid lithospheric thinning beneath the North China Craton? Geochim. Cosmochim. Acta 124, 250–271 (2014).

[51] X. Wang, Z. Wang, W. Zhang, L. Ma, W. Chen, Y.-C. Cai, S. Foley, C. Y. Wang, J. Li, J. Deng, Y. Feng, K. Zong, Z. Hu, Y. Liu, Sulfur isotopes of lamprophyres and implications for the control of metasomatized lithospheric mantle on the giant Jiaodong gold deposits, Eastern China. GSA Bull. 136, 340–3418 (2024).

[52] K.-F. Qiu, J. Deng, S.-X. Sai, H.-C. Yu, M. T. Tamer, Z.-J. Ding, X.-F. Yu, R., Goldfarb, low-temperature thermochronology for defining the tectonic controls on heterogeneous gold endowment across the Jiaodong Peninsula, Eastern China. Tectonics 42, e2022TC007669 (2023).

[53] H. R. Marschall, V. D. Wanless, N. Shimizu, P. A. E. Pogge von Strandmann, T. Elliott, B. D. Monteleone, The boron and lithium isotopic composition of mid-ocean ridge basalts and the mantle. Geochim. Cosmochim. Acta 207, 102–138 (2017).

[54] J. E. Saunders, N. J. Pearson, S. Y. O’Reilly, W. L. Griffin, Sulfide metasomatism and the mobility of gold in the lithospheric mantle. Chem. Geol. 410, 149–161 (2015).

[55] Z. Wang, H. Cheng, K. Zong, X. Geng, Y. Liu, J. Yang, F. Wu, H. Becker, S. Foley, C. Y. Wang, Metasomatized lithospheric mantle for Mesozoic giant gold deposits in the North China craton. Geology 48, 169–173 (2020).

[56] H. Palme, H. St. C. O’Neill, Cosmochemical Estimates of Mantle Composition, in *Treatise on Geochemi*stry, H. D. Holland, K. K. Turekian, Eds. (Pergamon, Oxford, 2007), pp. 1–38; https://www.sciencedirect.com/science/article/pii/B0080437516021770.

[57] J. E. Saunders, N. J. Pearson, S. Y. O’Reilly, W. L. Griffin, Gold in the mantle: A global assessment of abundance and redistribution processes. Lithos 322, 376–391 (2018).

[58] D. A. Holwell, M. Fiorentini, I. McDonald, Y. Lu, A. Giuliani, D. J. Smith, M. Keith, M. Locmelis, A metasomatized lithospheric mantle control on the metallogenic signature of post-subduction magmatism. Nat. Commun. 10, 3511 (2019). 31383863 10.1038/s41467-019-11065-4 PMC6683204

[59] J. Hermann, C. J. Spandler, Sediment melts at sub-arc depths: An experimental study. J. Petrol. 49, 717–740 (2008).

[60] S. Y. O’Reilly, W. L. Griffin, Mantle metasomatism in *Metasomatism and the chemical transformation of rock* (Springer Berlin, 2013) pp. 471–533.

[61] F. P. Bierlein, S. Pisarevsky, Plume-related oceanic plateaus as a potential source of gold mineralization. Econ. Geol. 103, 425–430 (2008).

[62] D. I. Groves, K. C. Condie, R. J. Goldfarb, J. M. A. Hronsky, R. M. Vielreicher, Secular changes in global tectonic processes and their influence on the temporal distribution of gold-bearing mineral deposits. Econ. Geol. 100, 203–224 (2005).

[63] Y. Weiss, C. Class, S. L. Goldstein, T. Hanyu, Key new pieces of the HIMU puzzle from olivines and diamond inclusions. Nature 537, 666–670 (2016). 27595333 10.1038/nature19113

[64] L. Sauzéat, R. L. Rudnick, C. Chauvel, M. Garçon, M. Tang, New perspectives on the Li isotopic composition of the upper continental crust and its weathering signature. Earth Planet. Sci. Lett. 428, 181–192 (2015).

[65] M. Tang, R. L. Rudnick, C. Chauvel, Sedimentary input to the source of Lesser Antilles lavas: A Li perspective. Geochim. Cosmochim. Acta 144, 43–58 (2014).

[66] H. R. Marschall, P. A. E. Pogge von Strandmann, H.-M. Seitz, T. Elliott, Y. Niu, The lithium isotopic composition of orogenic eclogites and deep subducted slabs. Earth Planet. Sci. Lett. 262, 563–580 (2007).

[67] D. Prelević, D. E. Jacob, S. F. Foley, Recycling plus: A new recipe for the formation of Alpine–Himalayan orogenic mantle lithosphere. Earth Planet. Sci. Lett. 362, 187–197 (2013).

[68] C. G. Soder, R. L. Romer, Post-collisional potassic–ultrapotassic magmatism of the Variscan orogen: Implications for mantle metasomatism during continental subduction. J. Petrol. 59, 1007–1034 (2018).

[69] K. M. Abdelfadil, R. L. Romer, J. Glodny, Mantle wedge metasomatism revealed by Li isotopes in orogenic lamprophyres. Lithos 196–197, 14–26 (2014).

[70] L. Dong, Z. Yang, Y. Liu, M. Song, Possible source of Au in the Jiaodong area from lower crustal sulfide cumulates: Evidence from oxygen states and chalcophile elements contents of mesozoic magmatic suites. Ore Geol. Rev. 153, 105268 (2023).

[71] L. D. Benton, J. G. Ryan, I. P. Savov, Lithium abundance and isotope systematics of forearc serpentinites, Conical Seamount, Mariana forearc: Insights into the mechanics of slab-mantle exchange during subduction. Geochem. Geophys. Geosyst. 5, Q08J12 (2004).

[72] T. Elliott, A. Thomas, A. Jeffcoate, Y. Niu, Lithium isotope evidence for subduction-enriched mantle in the source of mid-ocean-ridge basalts. Nature 443, 565–568 (2006). 17024091 10.1038/nature05144

[73] A. Giuliani, V. S. Kamenetsky, D. Phillips, M. A. Kendrick, B. A. Wyatt, K. Goemann, Nature of alkali-carbonate fluids in the sub-continental lithospheric mantle. Geology 40, 967–970 (2012).

[74] Z.-X. Wang, S.-A. Liu, S. Li, D. Liu, J. Liu, Linking deep CO_2_ outgassing to cratonic destruction. Natl. Sci. Rev. 9, nwac001 (2022). 35673528 10.1093/nsr/nwac001 PMC9166544

[75] D. Liu, Z. Zhao, D.-C. Zhu, Y. Niu, E. Widom, F.-Z. Teng, D. J. DePaolo, S. Ke, J.-F. Xu, Q. Wang, X. Mo, Identifying mantle carbonatite metasomatism through Os–Sr–Mg isotopes in Tibetan ultrapotassic rocks. Earth Planet. Sci. Lett. 430, 458–469 (2015).

[76] D.-B. Tan, Y. Xiao, L.-Q. Dai, H. Sun, Y. Wang, H.-O. Gu, Differentiation between carbonate and silicate metasomatism based on lithium isotopic compositions of alkali basalts. Geology 50, 1150–1155 (2022).

[77] D. Prelević, C. Akal, S. F. Foley, R. L. Romer, A. Stracke, P. Van Den Bogaard, Ultrapotassic mafic rocks as geochemical proxies for post-collisional dynamics of orogenic lithospheric mantle: The case of Southwestern Anatolia, Turkey. J. Petrol. 53, 1019–1055 (2012).

[78] D. Prelević, C. Akal, R. L. Romer, R. Mertz-Kraus, C. Helvacı, Magmatic response to slab tearing: Constraints from the Afyon alkaline volcanic complex, Western Turkey. J. Petrol. 56, 527–562 (2015).

[79] S. Graham, D. Lambert, S. Shee, The petrogenesis of carbonatite, melnoite and kimberlite from the Eastern Goldfields province, Yilgarn Craton. Lithos 76, 519–533 (2004).

[80] J. M. McArthur, R. J. Howarth, G. A. Shields, Y. Zhou, Strontium isotope stratigraphy, in *Geologic Time Scale 2020*, F. M. Gradstein, J. G. Ogg, M. D. Schmitz, G. M. Ogg, Eds. (Elsevier, 2020), pp. 211–238.

[81] Z.-Y. Zhu, T. Yang, X.-K. Zhu, Achieving rapid analysis of Li isotopes in high-matrix and low-Li samples with MC-ICP-MS: New developments in sample preparation and mass bias behavior of Li in ICPMS. J. Anal. At. Spectrom 34, 1503–1513 (2019).

[82] G. D. Flesch, A. R. Anderson, H. J. Svec, A secondary isotopic standard for ^6^Li/^7^Li determinations. Int. J. Mass Spectrom. 12, 265–272 (1973).

[83] S. S. Sun, W. F. McDonough, Chemical and isotopic systematics of oceanic basalts: Implications for mantle composition and processes. Geol. Soc. Lond. Spec. Publ. 42, 313–345 (1989).

[84] L. Hong, Y. Xu, L. Zhang, Z. Liu, X. Xia, Y. Kuang, Oxidized late mesozoic subcontinental lithospheric mantle beneath the eastern North China Craton: A clue to understanding cratonic destruction. Gondw. Res. 81, 230–239 (2020).

[85] G. Zhao, M. Sun, S. A. Wilde, L. Sanzhong, Late Archean to Paleoproterozoic evolution of the North China Craton: Key issues revisited. Precambrian Res. 136, 177–202 (2005).

[86] T. M. Kusky, A. Polat, B. F. Windley, K. C. Burke, J. F. Dewey, W. S. F. Kidd, S. Maruyama, J. P. Wang, H. Deng, Z. S. Wang, C. Wang, D. Fu, X. W. Li, H. T. Peng, Insights into the tectonic evolution of the North China Craton through comparative tectonic analysis: A record of outward growth of Precambrian continents. Earth. Sci. Rev. 162, 387–432 (2016).

[87] R.-X. Zhu, J.-H. Yang, F.-Y. Wu, Timing of destruction of the North China Craton. Lithos 149, 51–60 (2012).

[88] B. F. Windley, D. Alexeiev, W. Xiao, A. Kröner, G. Badarch, Tectonic models for accretion of the Central Asian orogenic belt. J. Geol. Soc. London 164, 31–47 (2007).

[89] Y.-F. Zheng, Z.-F. Zhao, R.-X. Chen, “Ultrahigh-pressure metamorphic rocks in the Dabie–Sulu orogenic belt: Compositional inheritance and metamorphic modification” in *HP–UHP Metamorphism and Tectonic Evolution of Orogenic Belts*, L. Zhang, Z. Zhang, H.-P. Schertl, C. Wei, Eds. (Geological Society of London, 2019), vol. 474, p. 0; 10.1144/SP474.9.

[90] H.-Y. Li, X.-L. Huang, Constraints on the paleogeographic evolution of the North China Craton during the late Triassic–Jurassic. J. Asian Earth Sci. 70-71, 308–320 (2013).

[91] Z. Wu, C. Lu, L. Qiu, H. Zhao, H. Wang, W. Tan, M. Zhong, New detrital zircon geochronological results from the Meso-Neoproterozoic sandstones in the southern-eastern Liaoning region, North China Craton, and their paleogeographic implications. Precambrian Res. 381, 106847 (2022).

[92] D.-B. Yang, H.-T. Yang, J.-P. Shi, W.-L. Xu, F. Wang, Sedimentary response to the paleogeographic and tectonic evolution of the southern North China Craton during the late paleozoic and mesozoic. Gondw. Res. 49, 278–295 (2017).

[93] T. M. Kusky, J. Li, Paleoproterozoic tectonic evolution of the North China Craton. J. Asian Earth Sci. 22, 383–397 (2003).

[94] Y. Wang, L. Zhou, S. Liu, J. Li, T. Yang, Post-cratonization deformation processes and tectonic evolution of the North China Craton. Earth Sci. Rev. 177, 320–365 (2018).

[95] H.-F. Zhang, M. Sun, X.-H. Zhou, W.-M. Fan, M.-G. Zhai, J.-F. Yin, Mesozoic lithosphere destruction beneath the North China Craton: Evidence from major-, trace-element and Sr–Nd–Pb isotope studies of Fangcheng basalts. Contrib. Mineral. Petrol. 144, 241–254 (2002).

[96] Q.-L. Yang, Z.-F. Zhao, Y.-F. Zheng, Modification of subcontinental lithospheric mantle above continental subduction zone: Constraints from geochemistry of Mesozoic gabbroic rocks in Southeastern North China. Lithos 146–147, 164–182 (2012).

[97] X. Wang, Z. Wang, H. Cheng, S. Foley, L. Xiong, Z. Hu, Early cretaceous lamprophyre dyke swarms in Jiaodong Peninsula, eastern North China Craton, and implications for mantle metasomatism related to subduction. Lithos 368–369, 105593 (2020).

[98] F.-Y. Wu, J.-H. Yang, Y.-G. Xu, S. A. Wilde, R. J. Walker, Destruction of the North China Craton in the Mesozoic. Annu. Rev. Earth Planet. Sci. 47, 173–195 (2019).

[99] S.-G. Li, W. Yang, S. Ke, X. Meng, H. Tian, L. Xu, Y. He, J. Huang, X.-C. Wang, Q. Xia, W. Sun, X. Yang, Z.-Y. Ren, H. Wei, Y. Liu, F. Meng, J. Yan, Deep carbon cycles constrained by a large-scale mantle Mg isotope anomaly in Eastern China. Natl. Sci. Rev. 4, 111–120 (2017).

[100] Y. Liang, X. Liu, C. Qin, Y. Li, J. Chen, J. Jiang, Petrogenesis of early Cretaceous mafic dikes in southeastern Jiaolai Basin, Jiaodong Peninsula, China. Intl. Geol. Rev. 59, 131–150 (2017).

[101] X. Liu, J. Deng, Y. Liang, Q. Wang, G. Li, Y. Ma, L. Xu, Y. Lu, Geochemical, mineralogical and chronological studies of mafic-intermediate dykes in the Jiaodong Peninsula: Implications for late Mesozoic mantle source metasomatism and lithospheric thinning of the Eastern North China Craton. Intl. Geol. Rev. 62, 2239–2260 (2020).

[102] A. R. Woolley, S. C. Bergman, A. D. Edgar, M. J. Le Bas, R. H. Mitchell, N. M. Rock, B. H. Scott Smith, Classification of lamprophyres, lamproites, kimberlites, and the kalsilitic, melilitic, and leucitic rocks. Can. Mineral. 34, 175–186 (1996).

[103] A. Peccerillo, S. R. Taylor, Geochemistry of eocene calc-alkaline volcanic rocks from the Kastamonu area, Northern Turkey. Contr. Mineral. Petrol. 58, 63–81 (1976).

[104] S. Turner, N. Arnaud, J. Liu, N. Rogers, C. Hawkesworth, N. Harris, S. V. Kelley, P. Van Calsteren, W. Deng, Post-collision, shoshonitic volcanism on the Tibetan Plateau: Implications for convective thinning of the lithosphere and the source of ocean island basalts. J. Petrol. 37, 45–71 (1996).

[105] N. M. S. Rock, The nature and origin of lamprophyres: An overview. Geol. Soc. Lond. Spec. Publ. 30, 191–226 (1987).

[106] S. Duggen, K. Hoernle, P. van den Bogaard, D. Garbe-Schönberg, Post-collisional transition from subduction- to intraplate-type magmatism in the westernmost Mediterranean: Evidence for continental-edge delamination of subcontinental lithosphere. J. Petrol. 46, 1155–1201 (2005).

[107] D. A. Ionov, W. L. Griffin, S. Y. O’Reilly, Volatile-bearing minerals and lithophile trace elements in the upper mantle. Chem. Geol. 141, 153–184 (1997).

[108] T. Furman, D. Graham, Erosion of lithospheric mantle beneath the East African Rift system: Geochemical evidence from the Kivu volcanic province. Lithos 24, 237–262 (1999).

[109] R. E. Ernst, S. M. Jowitt, Large Igneous Provinces (LIPs) and metallogeny, in *Tectonics, Metallogeny, and Discovery: The North American Cordillera and Similar Accretionary Settings*, M. Colpron, T. Bissig, B. G. Rusk, J. F. H. Thompson, Eds. (Society of Economic Geologists, 2013), vol. 17, p. 0; https://doi.org/10.5382/SP.17.02.

[110] P. B. Tomascak, F. Tera, R. T. Helz, R. J. Walker, The absence of lithium isotope fractionation during basalt differentiation: New measurements by multicollector sector ICP-MS. Geochim. Cosmochim. Acta 63, 907–910 (1999).

[111] L. H. Chan, J. M. Edmond, G. Thompson, K. Gillis, Lithium isotopic composition of submarine basalts: Implications for the lithium cycle in the oceans. Earth Planet. Sci. Lett. 108, 151–160 (1992).

[112] R. L. Romer, A. Meixner, H.-J. Förster, Lithium and boron in late-orogenic granites–Isotopic fingerprints for the source of crustal melts? Geochim. Cosmochim. Acta 131, 98–114 (2014).

[113] R. L. Rudnick, P. B. Tomascak, H. B. Njo, L. R. Gardner, Extreme lithium isotopic fractionation during continental weathering revealed in saprolites from South Carolina. Chem. Geol. 212, 45–57 (2004).

[114] W. Fang, L.-Q. Dai, Y.-F. Zheng, Z.-F. Zhao, Basalt Mo isotope evidence for crustal recycling in continental subduction zone. Geochim. Cosmochim. Acta 334, 273–292 (2022).

[115] J. Deng, Q.-F. Wang, L. Zhang, S.-C. Xue, X.-F. Liu, L. Yang, L.-Q. Yang, K.-F. Qiu, Y.-Y. Liang, Metallogenetic model of Jiaodong-type gold deposits, Eastern China. Sci. China Earth Sci. 66, 2287–2310 (2023).

[116] S. Penniston-Dorland, X.-M. Liu, R. L. Rudnick, Lithium isotope geochemistry. Rev. Mineral. Geochem. 82, 165–217 (2017).

[117] X.-J. Wang, L.-H. Chen, A. W. Hofmann, T. Hanyu, H. Kawabata, Y. Zhong, L.-W. Xie, J.-H. Shi, T. Miyazaki, Y. Hirahara, Recycled ancient ghost carbonate in the Pitcairn mantle plume. Proc. Natl. Acad. Sci. U.S.A. 115, 8682–8687 (2018). 30104354 10.1073/pnas.1719570115 PMC6126754

[118] M. C. Johnson, T. Plank, Dehydration and melting experiments constrain the fate of subducted sediments. Geochem. Geophys. Geosyst. 1, 1007 (2000).

[119] Y.-R. Qu, S.-A. Liu, H. Wu, M.-L. Li, H.-C. Tian, Tracing carbonate dissolution in subducting sediments by zinc and magnesium isotopes. Geochim. Cosmochim. Acta 319, 56–72 (2022).

[120] V. J. M. Salters, A. Stracke, Composition of the depleted mantle. Geochem. Geophys. Geosyst. 5, Q05B07 (2004).

[121] S. Labanieh, C. Chauvel, A. Germa, X. Quidelleur, E. Lewin, Isotopic hyperbolas constrain sources and processes under the lesser antilles arc. Earth Planet. Sci. Lett. 298, 35–46 (2010).

[122] T. Plank, C. H. Langmuir, The chemical composition of subducting sediment and its consequences for the crust and mantle. Chem. Geol. 145, 325–394 (1998).

[123] C. Ma, C. Ehlers, C. Xu, Z. Li, K. Yang, The roots of the dabieshan ultrahigh-pressure metamorphic terrane: Constraints from geochemistry and Nd–Sr isotope systematics. Precambrian Res. 102, 279–301 (2000).

[124] F. Ridolfi, A. Zanetti, A. Renzulli, D. Perugini, F. Holtz, R. Oberti, AMFORM, a new mass-based model for the calculation of the unit formula of amphiboles from electron microprobe analyses. Am. Mineral. 103, 1112–1125 (2018).

[125] F. Ridolfi, A. Renzulli, M. Puerini, Stability and chemical equilibrium of amphibole in calc-alkaline magmas: An overview, new thermobarometric formulations and application to subduction-related volcanoes. Contrib. Mineral. Petrol. 160, 45–66 (2010).

[126] X. Liu, “Deep magmatic process of the Early Cretaceous gold metallogenic system in the eastern North China Craton: Evidence from in-situ geochemical analyses of minerals and melt inclusions from mantle xenoliths and intermediate-basic dykes,” thesis, China Univ. of Geoscience (2022).

[127] Y.-Y. Liang, “Petrogenesis of the Early Cretaceous Mafic Dikes and Metallogenic Dynamics in Jiaodong Peninsula,” thesis, China Univ. of Geosciences (Beijing) (2017).

