# Supplementary material for: The role of an oxidized lithospheric mantle in gold mobilization
Source: Sci Adv. 2024 Oct 11;10(41):eado6262. doi: 10.1126/sciadv.ado6262 (PMC11468961; doi:10.1126/sciadv.ado6262)
Supplement: Supplementary file 1 — Supplementary Text Figs. S1 to S5 Tables S1 to S7 References [file sciadv.ado6262_sm.pdf]

Science Advances
AAAS

Supplementary Materials for

The role of an oxidized lithospheric mantle in gold mobilization

Kun-Feng Qiu *et al.*

Corresponding author: Kun-Feng Qiu, kunfengqiu@qq.com; Jun Deng, djun@cugb.edu.cn

Sci. Adv. **10**, eado6262 (2024)
DOI: 10.1126/sciadv.ado6262

This PDF file includes:

Supplementary Text
Figs. S1 to S5
Tables S1 to S7
References

Supplementary Texts

NOTE 1: Multiple subduction and enrichment events in the NCC

The North China Craton (NCC) has been divided into three major blocks, namely the Western Block, the Eastern Block, and the intervening Trans-North China Orogen (*1*) (Fig. 1). The Eastern and Western blocks amalgamed along the Trans-North China Orogen. The assembly of the NCC involved multiple events of subduction and accretion of magmatic arcs during the Neoarchean to the Early Paleoproterozoic (*85*, *86*). Most of the presently exposed basement rocks of the NCC experienced high-grade metamorphism during the Paleoproterozoic (~1.90 to 1.85 Ga). After the erosion of lower-grade metamorphic rocks, the NCC remained a stable platform until the end of the Paleozoic (*87*). During much of the Phanerozoic, the margins of the NCC were affected by numerous subduction events, which led to the formation of orogens around the NCC. For example, the closure of the Paleo-Asian Ocean and the collision with the Siberian Craton in the Carboniferous and Early Permian resulted in the formation of the Xing-Meng Orogen to the north of the NCC (*88*). The Devonian collision of the Yangtze Block with the NCC during the closure of the Paleo-Tethys formed the Qinling orogen along the southern margin of the NCC. This suture was later reactivated and experienced continental subduction and ultra-high pressure (UHP) metamorphism in its eastern part (Dabie-Sulu belt) during the Late Permian to Triassic (*89*) (Fig. 1). The accretion of other continental blocks to growing Asia resulted in similar reactivation of older sutures along the Central Asian Orogenic Belt. These Phanerozoic collisional events (but also earlier, not well-documented interactions of the NCC with neighboring plates) modified the subcontinental lithospheric mantle (SCLM) at the margins of the NCC by introducing crustal material into the mantle.

Since the Paleoproterozic, the upper crustal rocks on the NCC have been eroded during multiple paleo-uplifts and transported and deposited at the margins of the NCC (*87*, *90–92*). The detrital material may have been involved in later subduction events and fertilized the SCLM. It is noteworthy that the composition of the subducted material does not necessarily reflect the composition of the preserved high-grade metamorphic basement because elements that are mobile under medium- and high-grade metamorphism or that partition strongly into crustal melts may have been lost from the high-grade rocks and transferred to the upper crust, which was eroded and transported to the margins of the NCC. Multiple episodes of subduction of continental material from the Archean to the Mesozoic reintroduced some of these shelf sediments into the mantle, fertilizing the SLCM along the margins of the NCC (*85*, *93*).

The chemical composition, oxidation state, and metal endowment of the lithospheric mantle of the NCC likely represent the superposition of several events of metasomatism. During the Mesozoic alone, the ancient SCLM of the NCC underwent two major episodes of mantle metasomatism, i.e., one associated with the Triassic subduction of Yangtze (South China) continental crust and one associated with the Jurassic subduction of the Paleo-Pacific plate (*94*). Metasomatic agents released from the subducted Yangtze crust have modified the overlying SCLM of the eastern NCC (*95*, *96*). The melts originating from the subduction of metamorphic rocks of old continental crust were incorporated into the SCLM of the NCC, which accounts for the enriched Sr-Nd-Pb isotope signature of Mesozoic SCLM (*35*, *97*). Moreover, the elemental and isotopic composition of the SCLM of the NCC was also affected by the subducted paleo-Pacific plate. The aqueous fluids and hydrous melts released from the Paleo-Pacific plate modified the SCLM of the eastern NCC (*41*, *98*). Fluids released from the Paleo-Pacific plate account for low Rb/Sr (0.02 to 0.13), and high Ba/Rb (12 to 54) of Early Cretaceous lamprophyres in the eastern NCC (*35*, *50*). The Early Cretaceous lamprophyres and Late Cretaceous and Cenozoic continental basalts from eastern China have light Mg isotope signatures that are interpreted to reflect contributions of subducted marine carbonates from the Paleo-Pacific plate to the mantle (*76*, *99*).

NOTE 2: Characterization of the studied lamprophyre samples

The samples comprise lamprophyre dikes from the Jiaobei Terrane and the Sulu orogenic belt of the Jiaodong Peninsula. The samples were collected at some distance from the gold lodes to avoid changes caused by the ore-forming fluids. The mineralogy, major and trace elements, Sr-Nd-Pb isotopic compositions, and zircon U-Pb isotope data of the studied samples have been documented in previous studies (*35*, *100*, *101*). Here is a summary:

Mineralogy

The lamprophyre samples are kersantites and spessartites (*102*), displaying porphyritic and a fine-grained panidiomorphic texture. The kersantite samples contain biotite (10 to 30%) and clinopyroxene (10 to 15%) phenocrysts in a groundmass consisting of biotite (20 to 35%), plagioclase (20 to 30%), clinopyroxene (5 to 10%), amphibole (5 to 10%), and carbonate minerals (~5%). The spessartite samples contain amphibole (25% to 35%) and clinopyroxene (5% to 10%) phenocrysts in a groundmass that is composed of amphibole (5% to 10%), biotite (10% to 15%), plagioclase (20% to 30%), alkali feldspar (5% to 10%), clinopyroxene (5% to 10%), and carbonate minerals (~5%). Both types of lamprophyres have accessory proportions of pyrite, magnetite, and apatite.

Major and trace elements

The lamprophyres have SiO_2 contents ranging from 44.3 to 57.9 wt%, K_2O contents from 1.49 to 4.33 wt%, and total alkali (Na_2O+K_2O) contents from 2.74 to 7.28 wt%, with a K_2O/Na_2O ratio of 0.5 to 2. These rocks are classified as high-K to shoshonitic rocks based on their K_2O contents (*103*) and as shoshonitic rocks based on their K_2O/Na_2O values (*104*). The lamprophyres are classified as basalt, trachy-basalt, and basaltic andesites according to their SiO_2 and Na_2O+K_2O contents (*103*, *105*). These samples also have high MgO contents, ranging from 5.83 to 14.52 wt%, high Mg# (55 to 78), and high contents of compatible elements (i.e., Cr = 150 to 838 ppm and Ni = 45 to 353 ppm), but low contents of TiO_2 (0.62 to 1.40 wt%), Al_2O_3 (9.48 to 15.7 wt%), P_2O_5 (0.21 to 0.95 wt%), and total Fe_2O_3 (5.92 to 9.41 wt%).

All of the lamprophyre samples display similar features in spidergrams normalized to the primitive mantle and REE profiles normalized to chondrite. The lamprophyres show significant LREE enrichment with high $(La/Yb)_N$ (12 to 38) and weak negative Eu anomalies (Eu/Eu* = 0.63 to 0.78). In spidergrams normalized to primitive mantle, the lamprophyres show enrichments in large ion lithophile elements (LILEs; e.g., Ba, K, Pb, and Sr) and depletions in high field strength elements (HFSEs; e.g., Nb, Ta, P, and Ti) (Fig. 5). Furthermore, the geochemical characteristics of the samples are similar to those of the Early Cretaceous (110 to 130 Ma) mafic dikes documented from Jiaodong (*35*, *50*).

Sr-Nd-Pb isotopes

The lamprophyres have initial $^{87}Sr/^{86}Sr$ isotope ratios ranging from 0.7072 to 0.7110 and εNd(t) values that range from –19.9 to –8.9. The samples exhibit $(^{206}Pb/^{204}Pb)_i$ isotope ratios of 16.74 to 17.50, $(^{207}Pb/^{204}Pb)_i$ isotope ratios of 15.43 to 15.54, and $(^{208}Pb/^{204}Pb)_i$ isotope ratios of 37.23 to 37.93. These isotopic signatures are similar to those obtained for Early Cretaceous (130 to 110 Ma) mafic dikes with arc-like geochemical signatures (*50*). Although the Sr, Nd, and Pb isotopic compositions of lamprophyres from the Sulu and Jiaobei terranes broadly overlap, the lamprophyres from the Sulu terrane have lower $^{87}Sr/^{86}Sr_i$ ratios (0.7079 to 0.7098 vs. 0.7088 to 0.7110), $^{207}Pb/^{204}Pb_i$ ratios (15.43 to 15.48 vs. 15.45 to 15.54), and $^{208}Pb/^{204}Pb_i$ ratios (37.25 to 37.53 vs. 37.27 to 37.93), and a wider range of $\varepsilon_{Nd(t)}$ values (–16.2 to –8.9 vs. –18.5 to –13.9) (Fig. S1).

Genesis of the lamprophyres
The lamprophyre samples display high SiO_2, MgO, and $Mg^{\#}$ values, as well as compatible element contents, and strong fractionation between LREE and HREE. Furthermore, they show enrichment of LILE and depletion of HFSE, similar to the contemporaneous arc-like mafic dikes (*50*). The enriched lithospheric mantle source of these mafic dikes is reflected in their arc-like trace-element profile, the Sr-Nd-Pb isotopic signatures of an enriched source, and high $Mg^{\#}$ values (*35*). The depletion of HFSE in spidergrams relative to neighboring elements on the primitive mantle-normalized diagram indicates subduction influence (*106*). The high K_2O contents and LILE enrichment patterns of the lamprophyres suggest a LILE-enriched mantle melt source containing phlogopite or amphibole (*107*), whereas the relatively low Rb/Sr (0.01 to 0.12) and high Ba/Rb (15.3 to 52.3) ratios of the lamprophyres favor amphibole in the metasomatized mantle (*108*). The lamprophyres were derived from low-degree partial melting of amphibole-bearing lherzolite under P conditions that based on K/Yb vs. Dy/Yb relationships correspond to the spinel-garnet transition (*106*).
SHRIMP and LA-ICP-MS zircon U-Pb ages of 130 to 110 Ma (*35*, *100*, *101*) indicate that these rocks were emplaced during an extensional phase at the end of lithospheric delamination at the base of NCB and the onset of major changes in the plate kinematics in the Pacific plate and the movement along the Tan-Lu fault system (*30*, *44*). The different Sr-Nd-Pb isotopic compositions of lamprophyres from the Jiaobei Terrane and the Sulu orogen demonstrate that the SCLM is heterogeneous on a local scale (*35*).

Evaluation of crustal assimilation
An increase in SiO_2 content due to crustal assimilation typically results in a corresponding increase in $(^{87}Sr/^{86}Sr)_i$ ratios and a progressive decrease in $\varepsilon_{Nd(t)}$ values, which is not observed in the lamprophyre samples. This suggests that crustal assimilation played an insignificant role during melt evolution (*35*, *50*). The $(Th/Yb)_{PM}$ values in mantle melt compositions are typically low, but these values can increase due to crustal assimilation (*109*). However, the studied lamprophyres exhibit a narrow range of $(Th/Yb)_{PM}$ values, indicating no significant effects due to crustal contamination (*35*). Simple mass balance indicates that more than 70% crustal contamination would be necessary to produce the $(Nb/Th)_N$, $(Th/Yb)_N$, and $(Nb/Yb)_N$ values of the lamprophyre samples, which is not a plausible scenario (*35*). Only a few inherited zircon crystals were recovered from the lamprophyre samples, also indicating that crustal contamination was minor during the evolution of the magmas.

Sulfide Segregation and PGE distribution during the formation of lamprophyres dykes
The Cu/Pd ratios of the lamprophyre dikes range from 24 to 166 and the Cu/Zr ratios range from 0.07 to 0.26. Such values are typical for rocks that crystallized from sulfide-saturated melts (*35*, *109*). Sulfide-saturated melts may be the result of low-degree partial melting with sulfides and highly chalcophile PGEs remaining in the mantle or of the segregation of sulfides from sulfide-saturated melts in the crust (*109*). The Ni/MgO and Cu/Zr ratios of the lamprophyres, as well as small changes in the Pd/Zr and Cu/Pd ratios, support the latter interpretation. The primitive mantle-normalized chalcophile element diagram for the lamprophyre dikes shows depletions in Ir, Ru, and Os relative to Pt, Pd, and Rh. This has been interpreted to be the result of the fractionation of laurite and Os-Ir-Ru alloys during the early stages of crystallization (*35*).

NOTE 3: Factors affecting the Li isotope composition of the samples
The following processes affecting the Li isotope composition of magmas, partial melting, fractional crystallization, crustal contamination, and post-magmatic alteration, are considered in this paper.

During partial melting, Li isotope fractionation is negligible, i.e., less than 0.35‰ at 1350°C (*53*), due to the moderate incompatibility of Li (*110*). The fractionation of mafic minerals may increase δ^7Li values slightly while substantially increasing Li concentrations of the evolved melts (*111*) (Fig. 2). The Jiaodong lamprophyres experienced fractionation of olivine and clinopyroxene, as shown by the wide-ranging MgO contents that positively correlate with CaO/Al_2O_3, Cr, and Ni (*35*). The calculations indicate (details in Note 4) that the variation in δ^7Li values and MgO contents for some Jiaodong samples is consistent with the fractionation of mafic minerals (Fig. S5), which may account for the high Li contents in some lamprophyres and the shift of δ^7Li to higher values by several permil (Fig. 2). It should be noted that some high-MgO samples, which may represent primitive magma compositions, also have high δ^7Li values (Fig. S5). Thus, the SCLM beneath the Jiaobei Terrane is highly heterogeneous with respect to its Li isotopic composition, with the lowest δ^7Li values being close to those of the depleted mantle and the highest δ^7Li values (15.3 ‰) being significantly higher than the values of altered oceanic crust (AOC) (*53*). The range of δ^7Li values in the lamprophyres could represent variable contributions of these end-member type sources. The Jiaobei lamprophyres have higher δ^7Li values than the Sulu lamprophyres. This could indicate that the lamprophyres from the Sulu Terrane received consistently smaller contributions from the high- δ^7Li source or that the SCLM beneath the Sulu Terrane had lower δ^7Li values. We prefer the second interpretation and illustrate this schematically in Fig. S5, showing three end-member type sources: A source-I with δ^7Li values similar to that of the normal mantle, a source-II with intermediate δ^7Li values, and a source-III with exceptionally high δ^7Li values (Fig. S5).
Crustal assimilation can modify the original δ^7Li values of magmas but seems to have been unimportant for the studied lamprophyre samples. Crustal assimilation during magma ascent, would lower the δ^7Li values of the lamprophyres (Fig. 2). Similarly, hydrothermal alteration and weathering lower the δ^7Li values of altered rocks as ^7Li partitions preferentially into the fluid phase (*112*, *113*). Therefore, the high δ^7Li values reflect the SCLM source of the lamprophyres and indirectly the subducted rocks that modified the SCLM.

NOTE 4: Modeling of element content and Li isotope variation of lamprophyre magmas during crystal fractionation

The high concentrations of compatible elements like Ni, Cr, and MgO, together with high $Mg^\#$ values in the Jiaodong lamprophyres suggest that fractional fractionation was not significant. Despite this, we made a detailed assessment to gauge the impact of fractional crystallization on the Li isotope compositions of the lamprophyric melts. The major fractionating phases of our sample sets are olivine and clinopyroxene, as shown by positive correlations between MgO and total Fe_2O_3, CaO/Al_2O_3, Cr, and Ni, and a negative correlation between MgO and SiO_2 (*35*). The absence of Sr and Eu anomalies argues against the fractionation of plagioclase. The lack of any correlation between the P_2O_5 and TiO_2 contents and the MgO values suggests that the fractionation of accessory minerals such as apatite and Fe-Ti oxides was insignificant. Therefore, we focus on the role of Ol and Cpx fractionation on the whole-rock Li isotopic composition. The variation of MgO content and δ^7Li values for the crystal fractionation was calculated using the following equations:

$$D = \Sigma(x_i * Kd_i)$$

$$C_{Mg\ liquids} = 0.6 * C_{MgO\ liquids}$$

$$C_{parental\ liquids} = C_{liquids(previous\ step)}$$

$$C_{liquids} = C_{parental\ liquids} * F^{D-1}$$

$$f = F/(F+D*(1-F))$$

$$\delta^7Li_{parental\text{-}liquids} = \delta^7Li_{liquids(previous\ step)}$$

$$\delta^7Li_{liquids} = \delta^7Li_{parental\text{-}liquids} + 1000 * Ln\alpha * Lnf$$

where D is the bulk partition coefficient; x_i is the proportion of the crystallizing phase; Kdi is the mineral-melt partition coefficient; $C_{\text{parental liquids}}$ is the content of Mg in the parental liquid; C_{liquids} is the content of Mg in the liquid after crystal fractionation; F represents the melt fraction; f represents the fraction of Li remaining in the liquid; and α represents the fractionation factor between minerals and liquids.

The partition coefficients (D_{Mg}^{Ol} = 3.04, D_{Mg}^{Cpx} = 1.35, D_{Li}^{Ol} = 0.27, and D_{Li}^{Cpx} = 0.35) were taken from Geochemical Earth Reference Model (GERM; https://earthref.org/GERM). The fractionation factor (α) of Li in igneous systems has not been determined. For the modeling, we used the bulk Li equilibrium isotope fractionation factor between mafic melt and mineral which was determined to be in the range of 0.992 to 0.998. We assumed the lamprophyre melts fractionated in two stages, i.e., Ol fractionated at MgO ≥ 8 wt.% and Ol and Cpx fractionated at lower MgO content. For the second stage, we modeled the fractionation for Ol : Cpx = 3 : 2, 2 : 1, and 1 : 1. The results indicate that the MgO contents vary little with different Ol : Cpx ratios (Table S4). Therefore, only the results for Ol : Cpx = 3 : 2 are shown in Fig. S5. The Li partition coefficients of Ol and Cpx in the melt are comparable (D_{Li}^{Ol} = 0.27 and D_{Li}^{Cpx} = 0.35). Therefore, we used a bulk partition coefficient of D_{Li} = 0.3 to model the Li isotope fractionation during the fractional crystallization, regardless of the Ol and Cpx proportions. The MgO value of 13.62 wt.% from source-I, source-II, and source-III is based on the average MgO of high-MgO samples. The variable δ^7Li values of the three sources were taken as 3.5‰ (mantle δ^7Li value), 8.77‰ (medium δ^7Li after averaging), and 15.31‰ (the highest δ^7Li value). For details on the parameters and results see Table S4.

We modeled the Li contents and δ^7Li values for crystal fractionation of partial melts derived from a source with the addition of carbonate-bearing sediments (Fig. 2). The equations used are the same as described above. We used a bulk Li equilibrium isotope fractionation factor between the melt and minerals of 0.994 and a D_{Li} of 0.3. The results of the calculations are shown in Table S5.

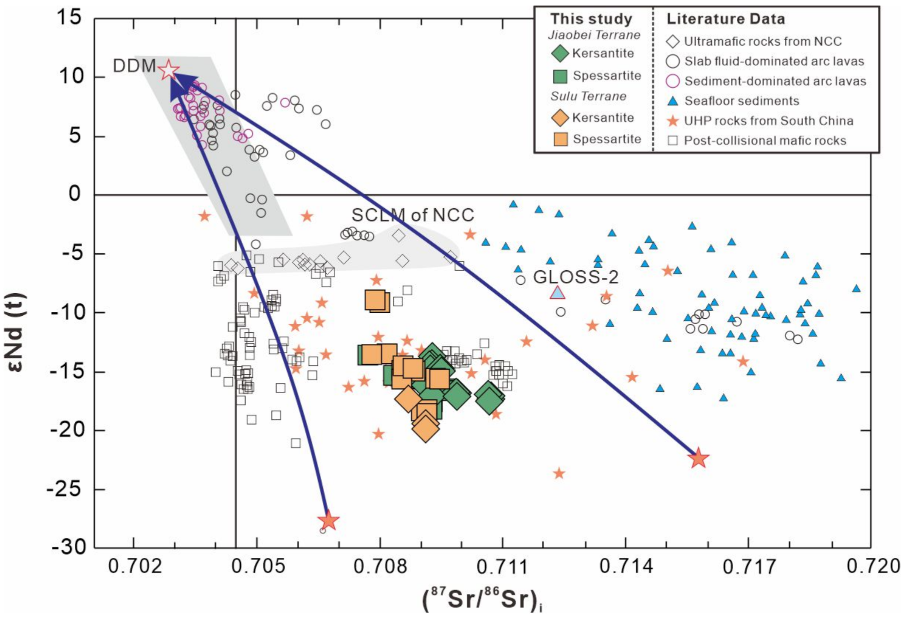

Fig. S1.
A binary plot of $\varepsilon_{Nd(t)}$ values vs. $(^{87}Sr/^{86}Sr)_i$ ratios for lamprophyres from the Jiaodong Au province. The literature data are from (*114*).

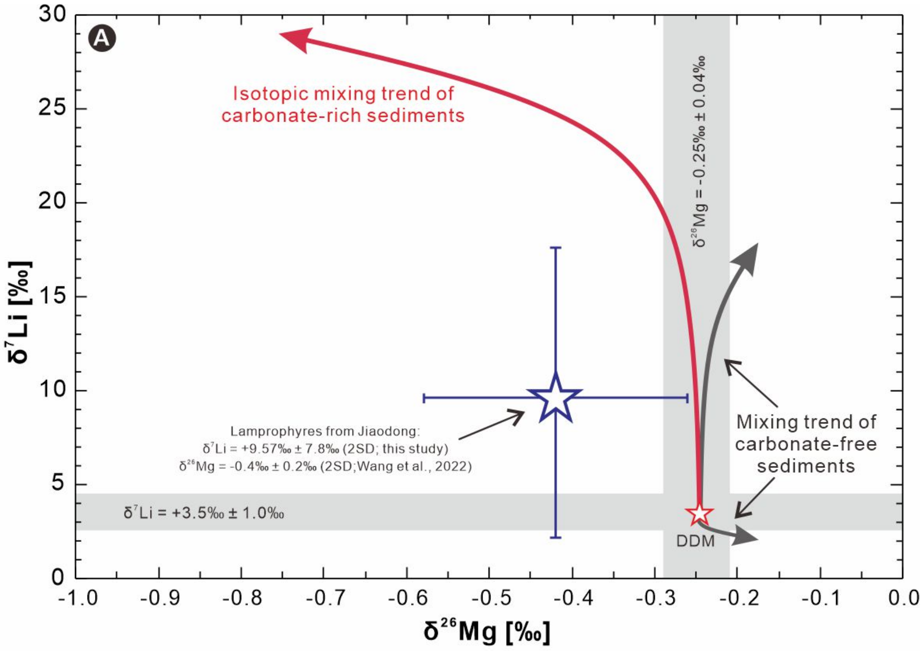

Fig. S2.
A plot showing two end-member mixing models of δ^7Li vs. δ^{26}Mg. The δ^7Li and δ^{26}Mg values of the depleted mantle are represented by the gray band. Values of the parameters for modeling are given in Table S3.

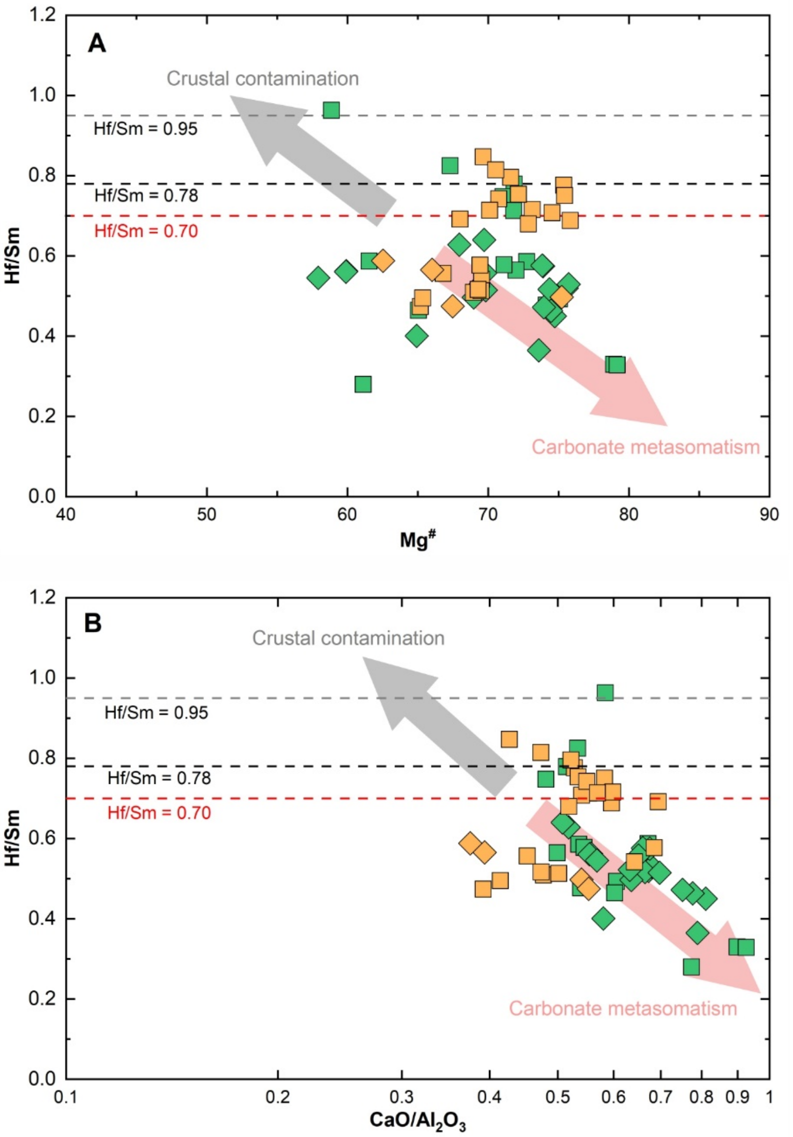

Fig. S3.

Plots of (A) Hf/Sm vs. $Mg^{\#}$ and (B) Hf/Sm vs. CaO/Al_2O_3, showing that the Jiaodong lamprophyres (high Mg#) are more depleted in HFSEs (i.e., Hf/Sm) and have higher CaO/Al_2O_3 (i.e., high CaO content) values. The symbols are the same as in Figure S1.

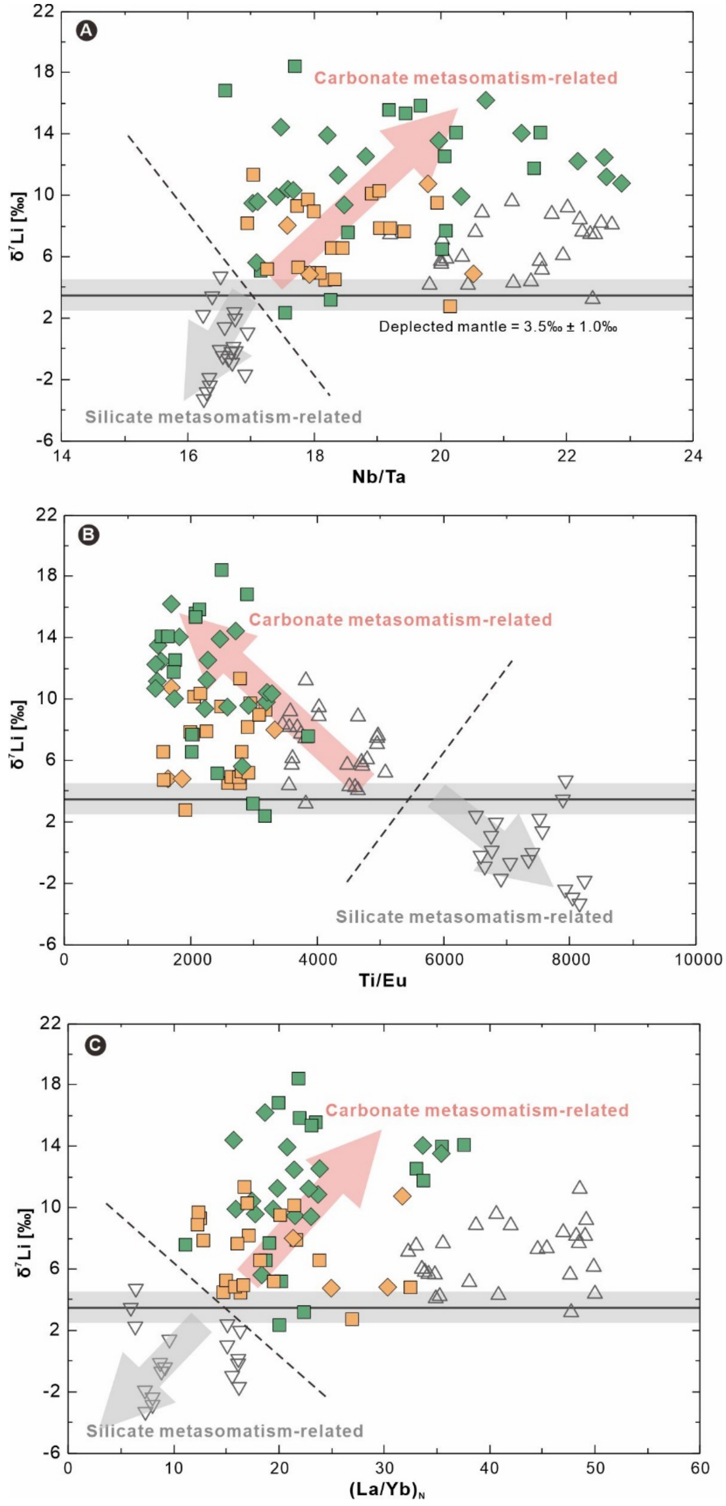

Fig. S4.

Plots of Li isotope ratios vs. (A) Nb/Ta, (B) Ti/Eu, and (C) $(La/Yb)_N$, showing the trends due to carbonate metasomatism and silicate metasomatism. The symbols are the same as in Figure S1. The trace element data for the Jiaodong lamprophyres are from (*35*, *100*, *101*).

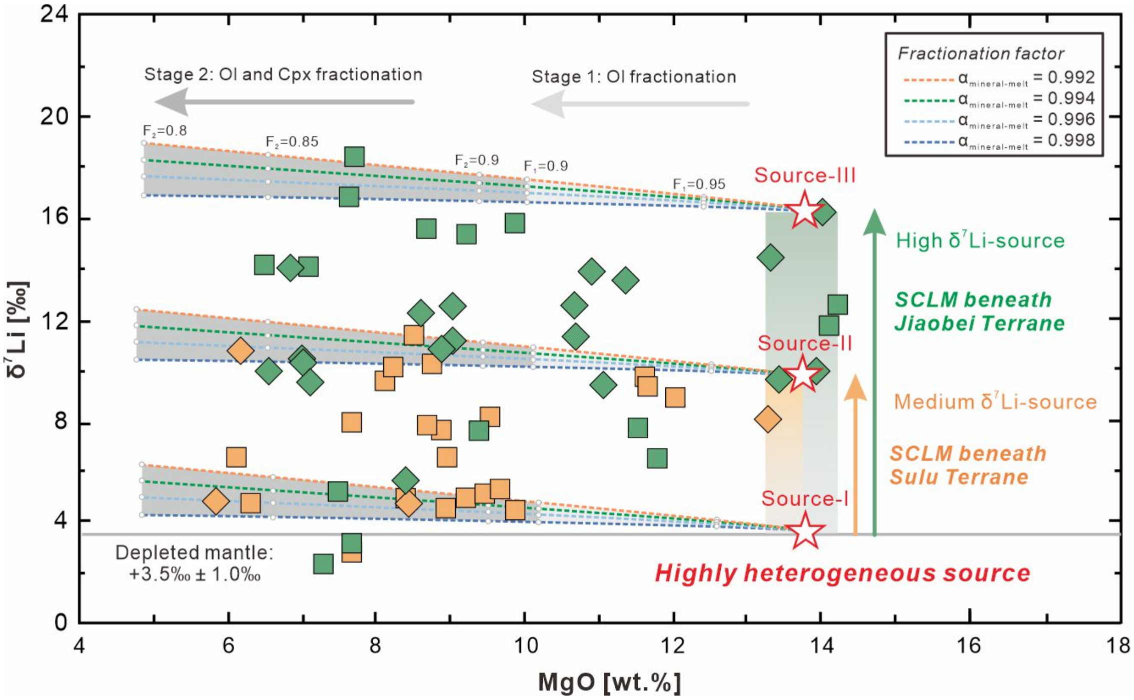

Fig. S5.

Modeled variations of δ^7Li values during crystal fractionation. The three sources differ in initial δ^7Li values, with sources I, II, and III having initial δ^7Li values of 3.5‰, 8.7‰, and 15.3‰, respectively. The symbols are the same as in Figure S1. See Supplementary Materials above and Tables S3, S4, and S5 (below) for detailed descriptions of the calculation of crystal fractionation and the values of the parameters used for mixing modeling.

Table S1.

δ^7Li (‰) values of standard reference materials.

Sample	d^{7}Li ± 2SD	Data source
BCR-2	3.19 ± 0.94	This study
Recommended value	3.13 ± 1.01	GeoREM
GSP-2	–0.72 ± 0.21	This study
Recommended value	–0.79 ± 0.24	GeoREM

Note: reference values are recommended values by GeoREM (http://georem.mpch-mainz.gwdg.de).

Table S2.

Chemical and isotopic compositions of rock samples from the Jiaodong gold province.

	Jiaobei Terrane									
Number	D2017-JD09	D2017-JD10	D2017-JD12	D2017-JD13	D2017-JD15	D2017-JD16	D2017-JD18	D2017-JD19	D2017-JD04	D2017-JD29
Type	Spessartite	Spessartite	Spessartite	Spessartite	Spessartite	Spessartite	Spessartite	Spessartite	Spessartite	Spessartite
δ^7Li(‰)	11.80	12.59	15.87	15.40	15.61	18.44	5.19	16.87	7.61	7.71
2SE(‰)	0.20	0.18	0.09	0.13	0.12	0.24	0.07	0.16	0.07	0.05
^{187}Os/^{188}Os(t)	0.3616	0.8134	0.8197	1.0326			0.5699	1.0345		0.7771
εNd(t)	-15.4	-15.6	-16.7	-16.4	-16.5	-18.2	-18.3	-17.6	-13.6	
Au(ppb)	0.38	0.44	1.63	6.95	9.25	0.43	0.41	0.82		2.31
Ag(ppb)	97	92	96	115	98	87	85	186		110
Os(ppb)	0.06	0.04	0.09	0.05	0.04	0.06	0.02	0.05		0.06
Ir(ppb)	0.03	0.03	0.04	0.04	0.02	0.02	<0.02	0.03		0.05
Ru(ppb)	0.05	0.05	0.06	0.05	0.04	0.04	<0.02	0.04		0.11
Rh(ppb)	0.03	0.03	0.03	0.03	0.02	<0.02	<0.02	0.02		0.09
Pt(ppb)	0.57	0.57	0.55	0.46	0.35	1.07	0.36	0.33		1.18
Pd(ppb)	0.47	0.49	0.38	0.37	0.29	0.27	0.30	0.43		1.19
SiO_2(wt%)	47.7	47.4	54.5	54.6	54.2	56.3	57.9	54.9	48.0	50.7
TiO_2	0.79	0.80	0.73	0.70	0.72	0.62	0.63	0.70	1.11	0.83
Al_2O_3	9.71	9.60	12.7	13.1	13.1	12.8	13.1	12.8	14.9	12.8
FeO	4.27	0.48	4.35	4.38	4.35	3.49	3.32	3.43	5.93	4.50
Fe_2O_3	2.70	6.86	2.47	2.23	2.12	2.10	2.33	2.11	2.44	2.96
MnO	0.08	0.08	0.10	0.09	0.09	0.08	0.09	0.07	0.18	0.11
MgO	14.1	14.2	9.84	9.20	8.65	7.70	7.47	7.62	9.38	11.5
CaO	8.73	8.90	6.80	6.55	7.14	6.60	6.30	7.06	7.92	6.89
Na_2O	1.71	1.64	2.54	2.84	2.72	2.81	2.99	2.27	1.81	2.58
K_2O	1.88	1.84	2.16	2.10	2.19	2.13	2.15	2.42	2.69	2.42
P_2O_5	0.47	0.50	0.41	0.39	0.38	0.23	0.23	0.26	0.47	0.52
LOI	7.69	7.47	3.14	3.67	4.18	4.91	3.30	6.18	4.13	3.99
Total	99.8	99.8	99.8	99.8	99.8	99.8	99.8	99.8	98.9	99.8
Li(ppm)	27.7	25.3	17.2	18.8	20.5	21.2	17.2	16.9	38.4	37.7
Be	1.04	1.14	1.23	1.24	1.27	1.18	1.28	1.19	2.03	1.35
B	1.50	1.38	2.58	3.78	3.47	0.95	0.84	1.13		5.20
La	85.8	84.4	52.6	55.6	56.8	40.0	40.4	34.5	31.9	53.2
Ce	175	176	111	117	119	82.3	90.1	74.4	64.6	124

(Continued)

Pr	19.3	19.5	13.0	13.4	13.6	9.30	9.94	8.72	7.46	14.9
Nd	71.5	72.0	48.1	49.4	50.0	33.9	36.6	32.6	28.2	56.8
Sm	10.6	10.9	7.63	7.49	7.78	5.14	5.51	5.13	5.76	9.08
Eu	2.75	2.75	2.05	2.03	2.08	1.51	1.56	1.46	1.73	2.49
Gd	7.54	8.01	5.87	5.81	5.93	4.09	4.29	4.00	5.44	6.81
Tb	0.93	0.94	0.74	0.76	0.76	0.54	0.58	0.53	0.75	0.87
Dy	4.20	4.23	3.63	3.58	3.64	2.69	2.86	2.55	4.14	4.16
Ho	0.71	0.74	0.65	0.63	0.63	0.48	0.51	0.45	0.78	0.72
Er	1.96	2.05	1.75	1.79	1.80	1.33	1.45	1.28	2.12	2.05
Tm	0.26	0.27	0.24	0.25	0.25	0.19	0.20	0.18	0.33	0.29
Yb	1.72	1.73	1.62	1.63	1.63	1.24	1.37	1.17	1.94	1.88
Lu	0.28	0.28	0.27	0.27	0.27	0.22	0.23	0.19	0.29	0.30
Rb	44.0	43.3	52.7	47.7	50.4	62.7	61.4	52.9	108	54.3
Ba	1961	1975	1678	1657	1727	1487	1386	1726	1830	2324
Th	12.0	11.8	7.55	8.29	8.76	6.41	6.84	7.17	2.62	8.54
U	1.86	1.89	0.86	1.16	1.21	1.50	1.62	1.78	0.58	1.73
Nb	7.30	7.09	8.13	8.66	8.66	6.53	6.95	6.44	11.30	8.41
Ta	0.34	0.35	0.41	0.45	0.45	0.37	0.41	0.39	0.61	0.42
Pb	20.0	20.4	8.75	8.95	9.14	17.9	13.9	25.4	23.6	13.7
Sr	1026	1027	984	954	955	962	945	743	629	898
Zr	158	156	189	195	191	169	172	150	188	179
Hf	3.50	3.58	4.47	4.23	4.50	4.01	4.12	3.65	4.75	4.33
Y	20.8	21.0	18.6	18.3	18.6	13.6	14.7	13.1	19.9	21.1
V	179	178	148	146	146	129	124	127	206	166
Cr	816	816	507	489	466	293	269	295	466	417
Co	42.6	41.8	32.3	31.9	32.6	25.6	25.3	27.0	38.2	40.0
Ni	188	178	137	128	130	78.8	79.4	81.0	137	215
Sc	24.3	24.3	20.8	20.7	21.1	17.0	16.8	18.9	31.2	23.4
Cu	28.6	29.6	13.2	16.2	13.4	21.7	31.5	30.4	21.9	41.8
Zn	79.0	74.1	89.0	91.3	93.5	68.4	69.0	66.8	141	88.0
Ga	15.0	14.3	17.0	17.6	17.9	18.0	18.8	18.4	16.6	18.2
As	0.60	0.49	0.67	0.67	0.58	0.58	0.58	0.40	0.86	0.58
Mo	1.06	1.05	0.22	0.25	0.22	1.91	0.92	0.31	0.83	1.25
In	0.05	0.04	0.05	0.05	0.05	0.04	0.04	0.04	0.05	0.05
Sn	1.25	1.24	1.36	1.08	0.87	1.01	1.17	1.45	0.99	1.15
Sb	0.13	0.12	0.16	0.14	0.10	0.09	0.05	0.06	0.28	0.05

(Continued)

Cs	2.62	2.47	0.23	0.14	0.19	2.04	2.17	1.14	2.53	1.08
W	0.29	0.27	0.21	0.32	0.31	0.32	0.35	0.25	0.54	0.29
Re	2.91	1.92	3.02	2.37	0.66	2.39	1.98	1.83		4.69
Hg	12.6	10.4	5.34	5.34	4.79	9.23	10.4	8.12		7.57
Tl	0.31	0.30	0.27	0.26	0.27	0.41	0.42	0.38	0.63	0.33
Bi	0.04	0.04	0.06	0.08	0.06	0.10	0.08	0.04	0.06	0.05

Table S2*(Continued)*

	Jiaobei Terrane									
Number	D2017-JD30	D2017-JD124	D2017-JD125	D2017-JD126	D2017-JD127	D2017-JD128	D2017-JD21	D2017-JD23	D2017-JD24	D2017-JD25
Type	Spessartite	Spessartite	Spessartite	Spessartite	Spessartite	Kersantite	Kersantite	Kersantite	Kersantite	Kersantite
δ^7Li(‰)	6.51 /6.87	14.13	14.11	2.36	3.22	14.05	13.56	10.29	10.41	9.91
2SE(‰)	0.11 /0.02	0.07	0.10	0.26	0.00	0.23	0.00	0.05	0.08	0.02
$^{187}Os/^{188}Os(t)$							0.396		0.1081	
εNd(t)	-15.3						-17.1	-15.0	-15.1	-14.8
Au(ppb)	1.10						0.84	0.46	0.49	0.46
Ag(ppb)	97						117	114	113	145
Os(ppb)	0.06						0.03	0.02	<0.02	<0.02
Ir(ppb)	0.06						0.07	0.02	<0.02	0.00
Ru(ppb)	0.11						0.04	0.04	0.04	0.06
Rh(ppb)	0.09						0.07	0.02	0.02	<0.02
Pt(ppb)	1.31						1.00	0.32	0.26	0.37
Pd(ppb)	1.28						1.07	0.26	0.24	0.27
SiO_2(wt%)	49.1	52.8	52.6	48.5	49.4	50.6	46.5	48.4	48.4	48.7
TiO_2	0.81	0.45	0.41	1.08	0.72	0.56	0.88	1.37	1.40	1.39
Al_2O_3	12.6	13.2	11.2	13.9	13.9	14.1	11.3	14.7	14.7	14.6
FeO	4.41	4.77	4.86	6.53	4.25	4.08	4.74	5.97	6.03	6.05
Fe_2O_3	2.84	2.72	3.52	2.79	3.42	2.78	2.78	2.61	2.58	2.69
MnO	0.12	0.09	0.03	0.14	0.11	0.06	0.12	0.17	0.16	0.16
MgO	11.8	6.48	7.08	7.26	7.65	6.83	11.3	6.98	7.00	6.54
CaO	7.62	8.89	8.69	8.09	8.39	8.21	8.90	8.19	8.16	8.29
Na_2O	2.41	3.59	1.13	1.44	1.88	2.61	2.04	2.66	2.58	2.59
K_2O	2.51	2.02	3.90	3.11	1.99	3.97	2.79	3.05	3.05	3.13
P_2O_5	0.50	0.38	0.23	0.50	0.28	0.18	0.70	0.82	0.81	0.78
LOI	5.18	3.96	5.89	5.91	8.01	5.53	7.68	4.89	4.89	4.89
Total	99.8	99.4	99.6	99.2	100	996	998	99.8	99.8	99.8
Li(ppm)	40.2	35.5	29.5	25.2	33.3	30.1	23.2	37.8	35.3	41.1
Be	1.22	1.39	1.44	2.80	1.36	0.99	1.56	1.82	1.58	1.65
B	4.84						1.42	1.71	1.84	1.41
La	48.9	67.9	59.1	48.2	52.0	64.5	102	53.4	52.9	51.8
Ce	124	95.2	122	206	128	87.9	223	128	128	127
Pr	14.7	16.3	12.7	14.2	11.9	18.9	25.3	15.6	15.8	15.6
Nd	55.7	35.6	39.5	57.5	45.4	50.1	93.9	59.5	61.7	60.7
Sm	8.64	7.38	9.18	5.94	7.54	7.86	13.9	9.60	9.78	9.90

(Continued)

Eu	2.44	1.68	1.61	2.05	1.45	1.83	3.53	2.51	2.61	2.62
Gd	6.62	6.87	6.96	4.30	4.98	5.55	10.1	7.53	7.42	7.61
Tb	0.86	0.55	0.65	0.55	0.64	0.47	1.18	0.97	0.98	1.03
Dy	3.98	2.72	3.66	4.39	3.37	2.62	5.03	4.76	4.78	4.99
Ho	0.68	0.53	0.36	0.67	0.66	0.46	0.84	0.83	0.82	0.88
Er	1.96	1.95	1.55	3.84	2.14	1.95	2.29	2.35	2.26	2.44
Tm	0.27	0.17	0.15	0.21	0.25	0.20	0.30	0.34	0.32	0.34
Yb	1.76	1.22	1.12	1.58	1.57	1.29	1.93	2.09	2.04	2.20
Lu	0.30	0.25	0.27	0.38	0.20	0.21	0.32	0.35	0.33	0.36
Rb	58.3	69.5	87.8	61.2	44.5	79.2	65.1	74.4	73.0	73.6
Ba	2862	3041	2175	1262	1767	1894	2648	2083	2205	2351
Th	8.44	4.53	15.0	4.80	11.0	18.2	16.6	8.28	7.39	7.66
U	1.68	0.55	1.98	1.53	1.88	3.14	2.93	1.94	1.70	1.70
Nb	8.35	7.49	7.12	7.89	6.39	7.45	10.8	20.6	19.2	19.3
Ta	0.42	0.37	0.33	0.45	0.35	0.35	0.54	1.17	1.09	1.11
Pb	19.4	11.2	16.3	32.8	26.1	8.66	39.4	17.5	14.9	26.1
Sr	820	930	1161	1504	1044	1287	1372	1072	1114	1104
Zr	176	189	175	212	226	156	222	238	235	231
Hf	4.26	4.33	2.57	5.72	3.50	3.15	5.09	5.40	5.49	5.40
Y	19.8	18.4	15.0	11.6	16.3	17.9	23.9	23.9	23.8	25.1
V	171	178	171	195	149	124	187	151	148	151
Cr	397	447	497	269	329	511	494	154	150	154
Co	36.5	26.1	30.5	39.0	32.9	33.2	40.6	27.1	25.3	25.8
Ni	186	155	202	79.8	196	192	174	50.7	45.6	49.9
Sc	21.7	21.8	25.6	23.0	26.0	23.5	251	17.9	16.2	17.0
Cu	41.7	26.5	14.0	34.3	39.6	23.0	40.8	24.8	22.9	22.8
Zn	89.2	64.4	44.8	97.0	71.9	26.1	111	107	96.4	105
Ga	17.7	17.2	17.4	14.2	20.9	15.5	18.8	20.0	18.3	18.7
As	0.58						0.94	1.11	0.85	1.56
Mo	1.28						0.59	1.71	1.58	1.38
In	0.05						0.05	0.06	0.05	0.06
Sn	1.41						1.48	1.26	1.54	1.63
Sb	0.04						0.16	0.07	0.08	0.28
Cs	1.14	0.92	1.02	3.76	3.68	1.58	2.73	2.16	2.15	2.56
W	0.38						0.49	0.39	0.39	0.36
Re	3.62						3.17	2.58	1.87	2.60

(Continued)

Hg	8.12						7.01	17.0	15.9	12.0
Tl	0.40	0.46	0.41	0.82	0.70	0.54	0.41	0.40	0.42	0.40
Bi	0.06						0.08	0.05	0.04	0.04

Table S2 *(Continued)*

	Jiaobei Terrane									
Number	D2017-JD68	D2017-JD69	D2017-JD70	D2017-JD71	D2017-JD113	D2017-JD118	D2017-JD119	D2017-JD120	D2017-JD36	D2017-JD37
Type	Kersantite	Kersantite	Kersantite	Kersantite	Kersantite	Kersantite	Kersantite	Kersantite	Kersantite	Kersantite
δ^7Li(‰)	16.19	9.92	14.44 /14.59	9.60	13.94	9.38	11.30	12.56	10.80	12.22
2SE(‰)	0.04	0.04	0.32 /0.11	0.19	0.02	0.17	0.14	0.03	0.30	0.20
^{187}Os/^{188}Os(t)	0.1756	0.1866	0.2243	0.1981	0.8858	0.3769	0.3288		0.3485	0.3074
εNd(t)	-17.2	-17.1	-14.9	-14.6	-16.6	-16.7	-16.7	-16.9	-17.1	-17.5
Au(ppb)	0.83	0.66	0.73	0.79	0.72	0.81	1.19	0.86	0.49	0.48
Ag(ppb)	111	92	77	75	173	70	76	84	110	91
Os(ppb)	0.20	0.28	0.07	0.09	0.08	0.07	0.14	0.08	0.05	0.11
Ir(ppb)	0.13	0.22	0.05	0.05	0.05	0.07	0.04	0.08	0.13	0.18
Ru(ppb)	0.22	0.31	0.09	0.09	0.08	0.10	0.07	0.10	0.07	0.09
Rh(ppb)	0.08	0.13	0.04	0.04	0.05	0.07	0.03	0.06	0.16	0.15
Pt(ppb)	0.76	2.08	0.54	0.50	0.50	1.04	0.65	1.06	1.75	2.00
Pd(ppb)	0.62	1.02	0.52	0.42	0.43	1.00	0.61	1.06	1.88	1.81
SiO_2(wt%)	47.7	47.6	46.8	46.4	47.8	48.3	49.4	49.2	49.7	50.4
TiO_2	0.98	1.02	1.14	1.18	0.86	0.79	0.78	0.79	0.90	0.84
Al_2O_3	11.2	11.3	11.3	11.6	11.5	11.4	11.5	11.7	13.0	12.7
FeO	6.40	6.49	6.36	6.91	4.36	5.59	5.22	5.53	4.99	5.05
Fe_2O_3	1.84	1.64	2.21	1.73	2.81	1.20	1.46	1.29	2.37	1.96
MnO	0.11	0.12	0.11	0.11	0.10	0.18	0.16	0.17	0.12	0.11
MgO	14.0	14.0	13.3	13.4	11.2	11.1	10.7	10.7	8.89	8.61
CaO	7.55	7.66	7.67	7.67	7.74	9.28	8.96	8.78	8.24	8.05
Na_2O	1.97	2.03	1.69	1.79	1.67	1.00	1.03	1.09	2.08	2.18
K_2O	4.33	4.21	3.55	3.44	2.47	1.74	2.17	2.00	2.58	2.56
P_2O_5	0.87	0.94	0.73	0.74	0.42	0.31	0.32	0.33	0.95	0.88
LOI	2.83	2.95	5.01	4.71	8.87	8.94	8.09	8.31	6.01	6.45
Total	99.8	99.8	99.9	99.8	99.8	99.8	99.8	99.8	99.8	99.8
Li(ppm)	47.7	49.4	54.4	58.7	28.1	20.3	17.4	22.2	68.9	66.0
Be	1.83	2.11	1.45	1.32	1.12	1.05	0.94	1.05	2.62	2.17
B	1.01	0.76	1.23	1.31	2.69	0.70	0.70	0.86	0.80	0.73
La	43.7	46.4	32.4	34.6	39.8	54.7	52.1	55.1	66.9	57.6
Ce	128	131	83.8	84.8	89.0	116	111	115	154	138
Pr	16.2	16.4	10.9	10.7	10.7	13.5	12.9	13.1	18.9	16.9
Nd	68.2	67.8	44.1	43.1	40.7	50.2	48.3	48.5	74.4	67.3
Sm	12.7	12.5	8.22	8.10	6.51	7.78	7.42	7.54	13.2	12.1

(Continued)

Eu	3.46	3.50	2.52	2.42	1.85	2.12	2.08	2.09	3.74	3.40
Gd	9.80	9.64	6.80	6.65	5.07	5.87	5.71	5.76	10.71	9.71
Tb	1.26	1.26	0.96	0.90	0.67	0.74	0.73	0.74	1.36	1.26
Dy	5.61	5.57	4.65	4.47	3.24	3.55	3.46	3.44	6.00	5.77
Ho	0.86	0.85	0.75	0.72	0.57	0.64	0.60	0.60	0.96	0.91
Er	2.13	2.13	1.87	1.77	1.54	1.76	1.64	1.71	2.48	2.28
Tm	0.27	0.27	0.23	0.24	0.22	0.24	0.24	0.24	0.31	0.29
Yb	1.58	1.61	1.39	1.31	1.37	1.60	1.54	1.56	1.90	1.81
Lu	0.23	0.24	0.21	0.19	0.23	0.27	0.25	0.26	0.31	0.29
Rb	108	106	97.9	87.9	73.8	64.7	87.1	79.0	68.3	65.7
Ba	3409	3271	2525	1841	1695	1248	1335	1292	2781	2859
Th	6.77	6.86	3.87	3.89	6.63	6.88	7.24	7.15	9.30	8.45
U	1.11	1.61	0.89	0.90	1.07	1.40	1.33	1.42	1.49	1.31
Nb	11.2	11.4	16.1	16.3	7.25	5.79	5.56	6.02	11.8	10.9
Ta	0.54	0.56	0.92	0.95	0.40	0.31	0.30	0.32	0.52	0.49
Pb	8.30	5.44	7.43	7.42	8.62	97.65	58.46	88.01	8.79	9.43
Sr	909	913	849	808	772	813	855	813	1206	1070
Zr	266	273	191	190	168	148	150	152	294	281
Hf	6.57	6.61	4.73	4.66	3.94	3.50	3.44	3.56	6.55	6.33
Y	24.6	24.4	20.6	20.0	16.0	17.4	16.8	16.9	27.4	26.0
V	173	188	203	206	170	173	169	172	178	166
Cr	701	678	638	657	526	477	480	457	382	349
Co	45.4	45.0	45.1	46.7	39.7	34.4	35.9	34.3	35.2	32.2
Ni	353	336	250	256´	232	161	156	157	112	106
Sc	22.6	22.7	22.9	23.4	21.7	22.4	22.3	21.7	23.1	21.5
Cu	44.2	52.0	38.1	43.4	35.1	14.6	22.4	20.3	30.5	12.0
Zn	65.2	67.6	76.5	79.6	79.3	186	162	168	92.0	85.0
Ga	14.2	14.3	15.2	15.2	15.4	16.7	16.1	16.7	17.8	16.5
As	0.40	0.49	0.40	0.58	0.40	1.29	1.38	1.29	0.40	0.49
Mo	0.49	0.53	1.37	0.48	0.81	0.75	0.73	0.93	4.11	4.57
In	0.05	0.05	0.05	0.05	0.05	0.05	0.04	0.05	0.06	0.05
Sn	1.23	1.18	1.15	1.21	1.07	1.05	0.92	1.29	1.60	1.46
Sb	0.06	0.05	0.05	0.05	0.12	0.25	0.37	0.32	0.05	0.05
Cs	1.76	1.76	2.91	2.20	2.12	2.96	3.78	3.46	1.35	1.12
W	0.23	0.25	0.23	0.30	0.25	0.26	0.25	0.27	0.85	0.78
Re	1.84	2.81	3.10	2.59	2.97	1.56	1.97	2.66	3.83	4.35

(Continued)

Hg	11.5	8.68	9.79	12.0	14.4	9.55	13.2	12.6	8.94	13.2
Tl	0.69	0.68	0.59	0.48	0.39	0.69	0.93	0.84	0.44	0.45
Bi	0.04	0.03	0.04	0.04	0.04	0.05	0.05	0.07	0.12	0.12

Table S2 *(Continued)*

	Jiaobei Terrane				Sulu Terrane					
Number	D2017-JD38	D2017-JD39	D2017-JD43	D2017-JD44	D2017-JD51	D2017-JD53	D2017-JD129	D2017-JD61	D2017-JD45	D2017-JD46
Type	Kersantite	Kersantite	Kersantite	Kersantite	Kersantite	Kersantite	Kersantite	Kersantite	Spessartite	Spessartite
δ^7Li(‰)	12.48	11.19 /10.89	9.49	5.62	10.77	4.83	8.03	4.83	8.99	9.76
2SE(‰)	0.35	0.02 /0.12	0.26	0.37	0.11	0.04	0.03	0.03	0.23	0.35
^{187}Os/^{188}Os(t)	0.3181	0.2805	0.3693	0.5214					0.1775	0.2804
εNd(t)	-17.2	-17.4	-14.3	-13.9	-19.9	-19.5		-17.4	-15.4	-15.6
Au(ppb)	0.59	0.69	0.29	0.86					0.43	0.48
Ag(ppb)	117	131	89	96					100	92
Os(ppb)	0.32	0.05	0.20	0.04					0.24	0.07
Ir(ppb)	0.26	0.08	0.04	0.03					0.21	0.04
Ru(ppb)	0.43	0.05	0.10	0.05					0.39	0.11
Rh(ppb)	0.13	0.08	0.02	0.02					0.06	0.03
Pt(ppb)	1.62	1.71	0.32	0.33					0.71	0.57
Pd(ppb)	1.62	1.36	0.32	0.27					0.47	0.44
SiO_2(wt%)	49.	50.2	53.3	52.8	54.	54.3	47.5	50.5	50.2	51.5
TiO_2	0.87	0.85	0.99	1.12	0.73	0.70	0.68	0.88	0.76	0.76
Al_2O_3	12.4	12.7	13.5	13.5	14.9	14.9	13.1	14.6	12.0	12.6
FeO	5.26	4.93	3.78	4.56			5.03		5.73	5.30
$Fe2O_3$	1.88	2.24	2.44	2.16			3.06		1.21	1.62
MnO	0.12	0.13	0.10	0.11	0.10	0.09	0.11	0.11	0.13	0.11
MgO	9.03	9.00	7.10	8.40	6.17	5.83	13.3	8.40	12.0	11.6
CaO	8.65	8.24	7.00	6.84	5.87	5.60	7.08	8.06	7.15	6.63
Na_2O	1.86	2.01	2.51	2.39	3.63	3.31	1.77	3.98	1.95	2.16
K_2O	2.79	3.05	3.83	3.51	3.65	3.53	2.58	2.51	1.49	1.75
P_2O_5	0.91	0.92	0.64	0.68	0.45	0.43	0.24	0.51	0.26	0.27
LOI	6.76	5.54	4.53	3.72	3.30	3.52	5.14	1.81	6.87	5.53
Total	99.8	99.8	99.8	99.8	99.1	99.1	99.6	99.3	99.8	99.8
Li(ppm)	60.3	59.1	38.1	50.5	21.7	21.5	39.8	12.4	36.9	27.9
Be	2.27	2.51	1.65	1.95	1.44	1.42	1.60	1.23	0.85	0.95
B	0.73	0.80	0.74	0.73					1.45	2.09
La	57.0	54.8	49.6	44.9	72.9	73.5	45.0	73.3	30.4	30.5
Ce	141	135	124	116	136	137	142	144	64.9	64.5
Pr	17.2	16.9	14.6	14.4	14.5	14.5	14.8	15.8	7.81	7.68
Nd	68.8	67.6	55.8	54.9	53.0	53.1	49.5	59.8	30.0	29.6
Sm	12.2	12.2	8.82	8.83	8.12	8.04	8.63	9.58	5.10	5.01

(Continued)

Eu	3.45	3.46	2.32	2.39	2.59	2.56	1.23	2.84	1.48	1.55
Gd	9.67	9.66	6.66	6.67	7.51	7.58	7.38	8.77	4.29	4.20
Tb	1.25	1.30	0.84	0.86	0.76	0.76	0.51	0.91	0.63	0.62
Dy	5.66	5.73	3.93	4.07	3.64	3.64	2.69	4.55	3.32	3.27
Ho	0.88	0.91	0.68	0.69	0.65	0.66	0.53	0.83	0.63	0.61
Er	2.20	2.31	1.80	1.89	1.85	1.89	2.32	2.32	1.72	1.72
Tm	0.28	0.30	0.25	0.25	0.24	0.24	0.20	0.30	0.25	0.26
Yb	1.79	1.86	1.56	1.65	1.55	1.63	1.42	1.97	1.66	1.68
Lu	0.28	0.28	0.25	0.28	0.24	0.24	0.24	0.30	0.27	0.27
Rb	72.9	84.4	130	106	76.9	75.5	79.8	62.5	52.7	60.4
Ba	3355	3300	3652	4040	1817	1837	1837	1218	1347	1449
Th	8.49	8.60	9.91	9.33	10.2	9.90	4.76	9.20	3.82	3.72
U	1.34	1.35	1.98	1.85	2.21	2.56	1.02	1.97	0.74	0.72
Nb	11.3	11.3	21.5	23.2	9.90	9.50	5.45	8.20	5.51	5.51
Ta	0.50	0.50	1.26	1.36	0.50	0.53	0.31	0.40	0.31	0.31
Pb	7.19	7.40	13.9	11.7	19.7	33.8	6.97	12.1	15.4	14.3
Sr	1309	1228	1126	1167	680	674	1608	650	1433	1925
Zr	285	293	237	246 5	188	187	134	175	144	156
Hf	6.27	6.78	5.54	5.66	4.59	4.73	4.29	4.55	3.51	3.89
Y	25.2	26.3	18.8	19.6	16.8	16.8	16.8	20.0	17.6	17.3
V	174	171	143	151	115	113	151	171	169	168
Cr	386	378	196	245	258	247	323	267	584	557
Co	30.1	32.0	24.6	28.3	13.8	20.3	25.7	35.0	42.3	37.9
Ni	116	115	75.6	92.6	114	116	194	148	249	227
Sc	23.2	23.6	17.6	19.4	14.4	14.4	28.1	21.0	22.8	21.3
Cu	47.7	39.5	25.2	28.0	7.50	7.80	28.1	36.2	28.7	37.8
Zn	90.6	89.0	72.8	74.1	76.9	76.4	89.2	81.0	110	91.9
Ga	16.9	17.0	18.7	18.7	19.2	18.9	18.3	17.9	15.9	14.9
As	0.40	0.58	0.58	0.49					0.58	0.58
Mo	2.51	3.46	1.44	1.44	1.98	1.64		1.04	0.24	0.62
In	0.06	0.06	0.05	0.05	0.05	0.05		0.05	0.04	0.04
Sn	1.30	1.50	1.41	1.68					0.97	0.98
Sb	0.05	0.08	0.06	0.05					0.07	0.07
Cs	1.44	1.91	8.06	4.46	0.96	0.90	0.55	1.41	2.78	2.63
W	1.04	1.02	1.19	1.01					0.15	0.20
Re	2.68	2.26	1.68	3.05					3.28	2.08

(Continued)

Hg	13.2	16.3	15.7	16.9					8.68	8.68
Tl	0.46	0.50	0.91	0.66			0.99		0.35	0.36
Bi	0.11	0.13	0.09	0.22	0.31	0.62		0.08	0.05	0.04

Table S2*(Continued)*

	Sulu Terrane									
Number	D2017-JD47	D2017-JD54	D2017-JD55	D2017-JD59	D2017-JD64	D2017-JD65	D2017-JD67	D2017-JD86	D2017-JD87	D2017-JD88
Type	Spessartite	Spessartite	Spessartite	Spessartite	Spessartite	Spessartite	Spessartite	Spessartite	Spessartite	Spessartite
δ^7Li(‰)	9.35	4.76	6.58	2.80	5.22	8.22 /8.42	11.40	5.29	4.97	4.53
2SE(‰)	0.17	0.00	0.05	0.18	0.20	0.33 /0.03	0.04	0.03	0.16	0.15
^{187}Os/^{188}Os(t)						0.2935			0.1352	0.2035
εNd(t)	-15.7	-18.6	-18.2	-18.3	-16.0	-15.5	-15.6	-14.6	-14.5	-14.4
Au(ppb)	0.37					0.35	0.30	1.17	0.94	0.96
Ag(ppb)	84					84	91	75	55	66
Os(ppb)	0.07				0.05	0.03	0.04	0.04	0.03	0.07
Ir(ppb)	0.07				0.03	0.02	0.02	0.02	<0.02	0.07
Ru(ppb)	0.19				0.09	0.06	0.08	0.08	0.06	0.14
Rh(ppb)	0.05				0.03	<0.02	0.03	0.02	<0.02	0.04
Pt(ppb)	0.75				0.43	0.30	0.43	0.38	0.25	0.52
Pd(ppb)	0.56				0.25	<0.20	0.23	0.61	0.34	0.52
SiO_2(wt%)	50.9	55.4	55.2	51.9	52.0	52.4	52.8	50.1	50.3	49.8
TiO_2	0.77	0.66	0.67	1.00	0.71	0.64	0.64	0.83	0.83	0.82
Al_2O_3	12.2	14.7	14.7	15.4	12.1	12.4	13.1	13.8	14.9	13.7
FeO	5.28				4.19	4.19	4.15	4.53	4.71	4.61
Fe_2O_3	1.63				2.22	1.78	1.65	2.54	2.00	2.40
MnO	0.13	0.08	0.07	0.12	0.09	0.09	0.09	0.11	0.09	0.11
MgO	11.6	6.29	6.10	7.65	9.45	9.51	8.49	9.64	8.38	9.84
CaO	7.08	5.77	6.10	6.98	7.23	6.71	6.77	7.21	6.35	7.34
Na_2O	2.14	3.34	3.18	3.25	2.35	2.50	2.58	2.49	2.94	2.52
K_2O	1.55	2.43	2.62	2.96	1.89	1.72	2.10	2.25	2.89	2.18
P_2O_5	0.26	0.34	0.35	0.66	0.58	0.21	0.23	0.43	0.47	0.43
LOI	6.34	3.62	4.24	2.01	7.29	7.67	7.31	5.84	6.00	5.97
Total	99.8	99.3	99.6	99.4	99.8	99.8	99.9	99.8	99.9	99.8
Li(ppm)	33.5	24.4	30.3	24.0	21.5	54.1	50.3	24.1	22.2	21.6
Be	0.96	1.40	1.39	1.53		0.86	0.99	1.08	1.13	0.99
B	1.94					1.18	1.94	4.96	3.86	4.28
La	29.6	73.4	62.4	87.4	35.6	33.2	32.2	32.2	30.7	29.7
Ce	62.5	147	124	156	74.9	68.4	68.8	70.6	70.9	68.4
Pr	7.49	16.3	14.0	18.1	8.84	7.91	8.03	8.67	8.71	8.38
Nd	28.2	60.7	53.8	67.1	32.7	29.2	30.0	33.8	33.6	33.1
Sm	4.89	8.97	8.44	10.3	5.14	4.77	4.80	5.77	5.57	5.77

(Continued)

Eu	1.45	2.54	2.59	3.15	1.47	1.32	1.39	1.78	1.82	1.78
Gd	4.07	7.73	7.73	9.54	4.00	3.80	3.89	4.68	4.56	4.70
Tb	0.60	0.79	0.82	1.00	0.54	0.52	0.54	0.67	0.63	0.66
Dy	3.24	3.68	4.02	5.00	2.71	2.69	2.70	3.40	3.06	3.37
Ho	0.60	0.65	0.73	0.92	0.48	0.48	0.50	0.59	0.52	0.60
Er	1.71	1.82	2.06	2.57	1.36	1.36	1.42	1.62	1.41	1.60
Tm	0.25	0.24	0.27	0.34		0.20	0.20	0.23	0.20	0.22
Yb	1.60	1.53	1.76	2.19	1.23	1.31	1.30	1.45	1.25	1.37
Lu	0.26	0.24	0.26	0.33	0.20	0.22	0.22	0.23	0.20	0.24
Rb	54.8	72.5	57.4	76.5	57.3	50.9	60.0	58.6	74.3	56.4
Ba	1542	1233	1487	1534	1722	1400	1637	1577	1763	1728
Th	3.85	8.60	7.90	9.90	4.63	4.88	5.42	3.00	3.13	2.70
U	0.79	2.06	1.98	1.93	1.19	1.23	1.40	0.65	0.76	0.59
Nb	5.70	8.30	8.30	14.3	5.86	5.03	5.51	8.42	9.02	8.08
Ta	0.32	0.46	0.45	0.71	0.34	0.30	0.32	0.48	0.50	0.44
Pb	14.2	20.6	13.3	26.3	20.8	15.9	19.8	12.1	9.25	14.0
Sr	1446	586	543	670	1264	781	831	1925	890	943
Zr	146	164	161	228	152	139	144	176	183	166
Hf	3.67	4.25	4.18	5.75	3.68	3.38	3.26	4.59	4.71	4.35
Y	16.9	16.2	18.0	22.3	13.5	13.5	13.9	16.7	14.9	16.5
V	171	119	117	155	160	158	152	163	153	161
Cr	557	225	210	210	442	393	352	369	221	370
Co	40.8	16.5	18.2	26.9	32.7	29.1	28.0	34.5	28.5	32.1
Ni	238	128	114	127	139	112	96.3	147	96.3	137
Sc	23.3	17.6	17.2	19.1		18.6	19.7	23.1	17.7	21.8
Cu	28.1	23.7	24.6	66.3		25.8	33.6	45.4	38.6	34.8
Zn	108	70.9	52.7	92.3		63.5	65.2	80.3	80.0	74.1
Ga	16.4	18.2	17.2	18.8		16.3	17.8	18.2	18.9	16.9
As	0.58					0.49	0.49	0.58	0.67	0.49
Mo	0.28	0.41	0.42	0.85		0.81	0.85	0.91	0.80	0.89
In	0.05	0.06	0.05	0.06		0.04	0.03	0.05	0.05	0.04
Sn	0.92					0.76	0.87	1.01	0.87	1.17
Sb	0.04					0.06	0.05	0.03	0.05	0.07
Cs	2.60	1.43	1.72	1.41		4.14	3.71	1.10	0.91	0.93
W	0.15					0.18	0.26	0.21	0.25	0.20
Re	2.12					3.22	2.34	1.91	1.68	2.35

(Continued)

Hg	9.23					13.1	10.9	8.12	9.23	8.68
Tl	0.34					0.36	0.40	0.34	0.45	0.34
Bi	0.05	0.31	0.41	0.19		0.07	0.08	0.03	0.02	0.03

Table S2 *(Continued)*

	Sulu Terrane								
Number	D2017-JD89	D2017-JD90	D2017-JD91	D2017-JD104	D2017-JD107	D2017-JD108	D2017-JD109	D2017-JD110	D2017-JD111
Type	Spessartite	Spessartite	Spessartite	Spessartite	Spessartite	Spessartite	Spessartite	Spessartite	Spessartite
δ^7Li(‰)	4.93	4.53	6.59	9.56	10.31	7.68	7.89 /7.74	10.18	7.95
2SE(‰)	0.08	0.08	0.13	0.07	0.35	0.40	0.05 /0.12	0.10	0.08
^{187}Os/^{188}Os(t)	0.5113		0.1987			0.3285	0.2316		
εNd(t)	-14.9	-14.9	-14.8	-13.5	-13.4	-13.4	-13.5	-0.0	-9.1
Au(ppb)	0.60	0.27	0.58	0.25	0.23	0.28	0.20	0.19	0.28
Ag(ppb)	125	96	71	76	65	92	113	109	130
Os(ppb)	0.06	0.03	0.04	0.17	0.04	0.04	0.05	0.01	0.02
Ir(ppb)	0.04	0.02	0.04	0.07	0.03	0.07	0.03	0.02	0.02
Ru(ppb)	0.11	0.06	0.12	0.21	0.05	0.13	0.07	0.04	0.05
Rh(ppb)	0.03	<0.02	0.03	0.03	0.02	0.04	0.03	<0.02	<0.02
Pt(ppb)	0.38	0.34	0.32	0.29	0.27	0.39	0.24	<0.20	<0.20
Pd(ppb)	0.29	0.39	0.24	0.23	0.21	0.35	0.21	<0.20	0.21
SiO_2(wt%)	49.7	49.7	50.4	50.6	50.7	50.8	51.0	52.0	51.8
TiO_2	0.84	0.84	0.85	0.90	0.80	0.79	0.76	0.83	0.86
Al_2O_3	13.6	13.4	14.2	13.3	14.1	13.9	14.2	12.3	12.3
FeO	4.80	4.57	4.50	3.96	4.58	4.51	4.47	4.39	4.07
Fe_2O_3	2.18	2.45	2.39	2.64	2.70	2.69	2.64	2.29	2.63
MnO	0.11	0.12	0.11	0.10	0.12	0.12	0.12	0.12	0.14
MgO	9.17	8.91	8.94	8.10	8.73	8.86	8.66	8.21	7.66
CaO	7.45	7.62	6.70	8.55	6.70	6.96	6.72	8.42	8.52
Na_2O	2.57	2.64	2.59	2.35	2.56	2.17	2.10	1.77	1.77
K_2O	2.58	2.56	2.67	1.97	3.08	3.51	3.82	2.54	2.70
P_2O_5	0.50	0.51	0.51	0.55	0.58	0.59	0.56	0.44	0.44
LOI	6.36	6.54	5.91	6.79	5.29	4.98	4.73	6.49	6.93
Total	99.8	99.8	99.8	99.8	99.8	99.8	99.8	99.8	99.8
Li(ppm)	25.7	26.1	25.6	33.6	31.0	27.8	26.3	40.3	42.4
Be	1.30	1.34	1.25	1.42	1.50	1.50	1.52	1.38	1.96
B	2.97	2.85	5.57	0.80	0.80	1.13	1.14	0.75	0.70
La	33.8	34.1	36.0	50.5	42.4	44.8	36.7	48.5	44.6
Ce	80.3	80.9	78.7	113	114	110	119	103	97.1
Pr	9.78	9.92	9.36	13.2	13.0	13.5	12.5	12.0	11.7
Nd	37.9	38.4	36.0	50.1	49.9	52.0	50.3	48.8	46.6
Sm	6.29	6.46	5.83	7.91	7.95	8.44	8.37	8.69	8.26

(Continued)

Eu	1.92	1.97	1.82	2.17	2.22	2.33	2.29	2.44	2.31
Gd	4.95	5.10	4.80	6.12	6.02	6.24	6.27	6.58	6.26
Tb	0.67	0.67	0.65	0.79	0.79	0.82	0.83	0.84	0.79
Dy	3.31	3.40	3.19	3.86	3.77	3.98	4.08	3.92	3.72
Ho	0.57	0.59	0.56	0.65	0.66	0.71	0.72	0.63	0.59
Er	1.62	1.64	1.51	1.82	1.84	1.98	2.02	1.72	1.65
Tm	0.23	0.23	0.21	0.26	0.26	0.29	0.29	0.23	0.22
Yb	1.45	1.41	1.34	1.70	1.67	1.89	1.94	1.53	1.39
Lu	0.22	0.23	0.23	0.28	0.28	0.32	0.33	0.26	0.24
Rb	65.3	65.0	70.4	50.4	79.3	89.1	94.2	55.0	64.9
Ba	2018	2101	1888	2017	2736	2850	3076	1529	2158
Th	3.59	3.63	3.46	6.23	7.98	8.16	8.19	8.96	8.69
U	0.85	0.86	0.83	1.47	1.88	1.82	1.89	2.61	2.44
Nb	9.87	9.78	10.0	9.85	10.8	10.5	10.7	8.99	8.93
Ta	0.55	0.54	0.55	0.49	0.57	0.54	0.56	0.48	0.47
Pb	8.10	6.87	8.01	26.4	24.3	22.9	37.2	20.3	35.5
Sr	806	809	1541	1337	998	1003	977	4054	5114
Zr	186	185	192	187	189	192	191	228	240
Hf	4.67	4.61	4.75	4.29	4.05	4.33	4.32	5.02	5.71
Y	16.5	16.8	15.6	18.7	19.7	20.7	20.9	18.4	17.4
V	154	153	156	157	154	150	145	161	160
Cr	353	375	271	287	241	280	269	280	274
Co	32.7	31.4	31.8	32.8	31.0	30.9	31.5	30.0	29.4
Ni	150	135	117	135	123	124	128	66.1	63.7
Sc	20.2	20.7	18.4	19.0	17.7	19.4	19.4	20.6	19.8
Cu	28.5	18.8	25.5	29.8	23.3	28.1	30.1	34.3	34.8
Zn	89.6	92.5	87.0	104	110	101	102	69.6	95.9
Ga	17.6	18.1	18.6	19.6	19.6	19.1	19.5	17.7	17.7
As	0.49	0.49	0.58	0.94	1.29	1.11	1.20	0.94	1.20
Mo	0.55	0.58	1.11	1.59	0.37	0.26	0.34	1.33	1.32
In	0.05	0.04	0.05	0.05	0.05	0.05	0.05	0.04	0.04
Sn	1.20	1.31	1.27	1.30	1.19	1.43	1.01	1.25	1.29
Sb	0.07	0.05	0.06	0.05	0.05	0.05	0.04	0.03	0.04
Cs	0.75	0.70	0.84	2.25	0.53	0.55	0.57	2.06	2.76
W	0.21	0.22	0.21	0.27	0.52	0.44	0.42	0.45	0.50
Re	3.54	1.19	3.55	3.74	1.44	2.10	2.38	3.27	4.66

(Continued)

Hg	9.23	6.46	9.23	14.8	6.46	5.90	6.46	11.5	11.5
Tl	0.40	0.40	0.40	0.23	0.48	0.55	0.57	0.32	0.36
Bi	0.03	0.03	0.02	0.04	0.08	0.07	0.08	0.10	0.11

Table S2 *(Continued)*

	Jiaodong										
Number	112ZK9-642	112ZK9-1242	112ZK9-1322	112ZK9-1492	CD19D001B2	CD19D001B5	CD19D002B2	19LLBY1-1	17SSD08-1	JD21B4	JD21B12
Type	Gneiss	Gneiss	Amphibolite	Amphibolite	Linglong granite	Linglong granite	Linglong granite	Guojialing granitoid	Guojialing granitoid	Guojialing granitoid	Guojialing granitoid
δ^7Li(‰)	2.36	0.06	-1.11	-2.31	2.86	-1.76	-1.79	-1.10	2.71	0.24	3.62
2SE(‰)	0.26	0.13	0.09	0.14	0.20	0.25	0.09	0.04	0.11	0.20	0.25

Note: Li isotope data are from this study. Major, trace, and platinum group element, as well as Nd isotope data, are from references (*35*, *100*, *101*). Os isotope data are from (*115*). Duplicate analyses of Li from the same purified Li solution are separated by a slash "/".

Table S3.

Parameters and compositions of assimilated materials used for mixing modeling.

	δ^7Li (‰)	Li (ppm)	Data source
DDM	3.5	1.2	(*53*)
Carbonates	25	1.5	(*116*, *117*)
Pelagic sediments	2	50	(*53*)
Marine sediments (carbonate-rich)	25	83.6	(*65*, *116*, *118*)
Continental sedimentary rocks	–5.00	52.4	(*112*)
AOC	10	7.6	(*53*)
	$\delta^{26}Mg$ (‰)	MgO (wt.%)	
DDM	–0.25	7,58	(*119*)
Carbonate-free sediments	0.13	1.91	(*119*)
Carbonate-rich sediments	–0.85	1.29	(*119*)
	Nd (ppm)	$^{143}Nd/^{144}Nd$	
DDM	0.713	0.51301	(*120*, *121*)
Carbonate-rich sediments	22.6	0.51181	(*118*, *122*)
Continental crust	29.45	0.51104	(*123*)

Table S4.

Results of the modeling of MgO contents (wt.%) and Li isotope compositions (‰) during crystal fractionation.

**Selected partition coefficient for modeling**				
	**Ol**	**Cpx**		
$\mathbf{D_{Mg}}$	3.04	1.35		
$\mathbf{D_{Li}}$	0.27	0.35		
**Variations of MgO contents during crystal fractionation**				
Stage 1: Ol fractionation (D_{Mg}^{Ol} = 3.04)				
$\mathbf{F_1}$	$\mathbf{D_{ol}}$	$\mathbf{C_{Mg\text{-}parental\ liquids}}$	$\mathbf{C_{Mg\text{-}liquids}}$	$\mathbf{C_{MgO\text{-}liquids}}$
1	3.04	8.17	8.17	13.62
0.95	3.04	8.17	7.36	12.27
0.9	3.04	7.36	5.94	9.9
Stage 2: Ol and Cpx fractionation in the proportion of 3:2 ($D_{Mg}^{Ol:Cpx=3:2}$ **= 2.4**)				
$\mathbf{F_2}$	$\mathbf{D_{ol:cpx=3:2}}$	$\mathbf{C_{Mg\text{-}parental\ liquids}}$	$\mathbf{C_{Mg\text{-}liquids}}$	$\mathbf{C_{MgO\text{-}liquids}}$
1	2.36	5.94	5.94	9.9
0.95	2.36	5.94	5.54	9.23
0.9	2.36	5.54	4.8	7.99
0.85	2.36	4.8	3.84	6.4
0.8	2.36	3.84	2.83	4.72
Stage 2: Ol and Cpx fractionation in the proportion of 2:1 ($D_{Mg}^{Ol:Cpx=2:1}$ **= 2.5**)				
$\mathbf{F_2}$	$\mathbf{D_{ol:cpx=2:1}}$	$\mathbf{C_{Mg\text{-}parental\ liquids}}$	$\mathbf{C_{Mg\text{-}liquids}}$	$\mathbf{C_{MgO\text{-}liquids}}$
1	2.47	5.94	5.94	9.9
0.95	2.47	5.94	5.51	9.18

0.9	2.47	5.51	4.71	7.86
0.85	2.47	4.71	3.71	6.18
0.8	2.47	3.71	2.67	4.45

Stage 2: Ol and Cpx fractionation in the proportion of 1:1 ($D_{Mg}^{Ol:Cpx=1:1}$ **= 2.2***)*

F_2	$D_{ol:cpx=1:1}$	$C_{Mg\text{-parental liquids}}$	$C_{Mg\text{-liquids}}$	$C_{MgO\text{-liquids}}$
1	2.2	5.94	5.94	9.9
0.95	2.2	5.94	5.59	9.31
0.9	2.2	5.59	4.92	8.21
0.85	2.2	4.92	4.06	6.76
0.8	2.2	4.06	3.11	5.18

Variations of Li isotope compositions during crystal fractionation

Source-III: α = 0.992

Stage 1: olivine fractionation

F_1	D_{ol}	$C_{Li\text{-parental liquids}}$	$C_{Li\text{-liquids}}$	f_1	$\delta^7Li_{parental\text{-}liquids}$	α_{ol}	$\delta 7^{Li}_{liquids}$
1	0.3	41.09	41.09	1	15.31	0.99	15.31
0.95	0.3	41.09	42.58	0.98	15.31	0.99	15.44
0.9	0.3	42.58	45.79	0.97	15.44	0.99	15.7

Stage 2: olivine and clinopyroxene fractionation (D_{Li} *= 0.3)*

F_2	$D_{ol:cpx=3:2}$	$C_{Li\text{-parental liquids}}$	$C_{Li\text{-liquids}}$	f_2	$\delta^7Li_{parental\text{-}liquids}$	$\alpha_{ol:cpx=3:2}$	$\delta^7Li_{liquids}$
1	0.3	45.79	45.79	1	15.7	0.99	15.7
0.95	0.3	45.79	47.44	0.98	15.7	0.99	15.83
0.9	0.3	47.44	51	0.97	15.83	0.99	16.09
0.85	0.3	51	56.97	0.95	16.09	0.99	16.51
0.8	0.3	56.97	66.21	0.93	16.51	0.99	17.09

Source-III: α = 0.994

Stage 1: olivine fractionation

F_1	D_{ol}	$C_{Li\text{-parental liquids}}$	$C_{Li\text{-liquids}}$	f_1	$\delta^7Li_{parental\text{-}liquids}$	α_{ol}	$\delta 7^{Li}_{liquids}$
1	0.3	41.09	41.09	1	15.31	0.99	15.31
0.95	0.3	41.09	42.58	0.98	15.31	0.99	15.4
0.9	0.3	42.58	45.79	0.97	15.4	0.99	15.6

Stage 2: olivine and clinopyroxene fractionation (D_{Li} *= 0.3)*

F_2	$D_{ol:cpx=3:2}$	$C_{Li\text{-parental liquids}}$	$C_{Li\text{-liquids}}$	f_2	$\delta^7Li_{parental\text{-}liquids}$	$\alpha_{ol:cpx=3:2}$	$\delta^7Li_{liquids}$
1	0.3	45.79	45.79	1	15.6	0.99	15.6
0.95	0.3	45.79	47.44	0.98	15.6	0.99	15.7
0.9	0.3	47.44	51	0.97	15.7	0.99	15.9
0.85	0.3	51	56.97	0.95	15.9	0.99	16.21
0.8	0.3	56.97	66.21	0.93	16.21	0.99	16.65

Source-III: α = 0.996

Stage 1: olivine fractionation

F_1	D_{ol}	$C_{Li\text{-parental liquids}}$	$C_{Li\text{-liquids}}$	f_1	$\delta^7Li_{parental\text{-}liquids}$	α_{ol}	$\delta 7^{Li}_{liquids}$
1	0.3	41.09	41.09	1	15.31	1	15.31
0.95	0.3	41.09	42.58	0.98	15.31	1	15.37
0.9	0.3	42.58	45.79	0.97	15.37	1	15.5

Stage 2: olivine and clinopyroxene fractionation (D_{Li} *= 0.3)*

**F_2**	**$D_{ol:cpx=3:2}$**	**$C_{Li\text{-}parental\ liquids}$**	**$C_{Li\text{-}liquids}$**	**f_2**	**$\delta^7Li_{parental\text{-}liquids}$**	**$\alpha_{ol:cpx=3:2}$**	**$\delta^7Li_{liquids}$**
1	0.3	45.79	45.79	1	15.5	1	15.5
0.95	0.3	45.79	47.44	0.98	15.5	1	15.57
0.9	0.3	47.44	51	0.97	15.57	1	15.7
0.85	0.3	51	56.97	0.95	15.7	1	15.91
0.8	0.3	56.97	66.21	0.93	15.91	1	16.2
**Source-III: α = 0.998**							
Stage 1: olivine fractionation							
**F_1**	**D_{ol}**	**$C_{Li\text{-}parental\ liquids}$**	**$C_{Li\text{-}liquids}$**	**f_1**	**$\delta^7Li_{parental\text{-}liquids}$**	**α_{ol}**	**$\delta 7^{Li}_{liquids}$**
1	0.3	41.09	41.09	1	15.31	0.998	15.31
0.95	0.3	41.09	42.58	0.984456	15.31	0.998	15.34136
0.9	0.3	42.58	45.79	0.967742	15.34136	0.998	15.40701
Stage 2: olivine and clinopyroxene fractionation (D_{Li} = 0.3)							
**F_2**	**$D_{ol:cpx=3:2}$**	**$C_{Li\text{-}parental\ liquids}$**	**$C_{Li\text{-}liquids}$**	**f_2**	**$\delta^7Li_{parental\text{-}liquids}$**	**$\alpha_{ol:cpx=3:2}$**	**$\delta^7Li_{liquids}$**
1	0.3	45.79	45.79	1	15.41	1	15.41
0.95	0.3	45.79	47.44	0.98	15.41	1	15.44
0.9	0.3	47.44	51	0.97	15.44	1	15.5
0.85	0.3	51	56.97	0.95	15.5	1	15.61
0.8	0.3	56.97	66.21	0.93	15.61	1	15.75
**Source-II: α = 0.992**							
Stage 1: olivine fractionation							
**F_1**	**D_{ol}**	**$C_{Li\text{-}parental\ liquids}$**	**$C_{Li\text{-}liquids}$**	**f_1**	**$\delta^7Li_{parental\text{-}liquids}$**	**α_{ol}**	**$\delta 7^{Li}_{liquids}$**
1	0.3	41.09	41.09	1	8.77	0.99	8.77
0.95	0.3	41.09	42.58	0.98	8.77	0.99	8.9
0.9	0.3	42.58	45.79	0.97	8.9	0.99	9.16
Stage 2: olivine and clinopyroxene fractionation (D_{Li} = 0.3)							
**F_2**	**$D_{ol:cpx=3:2}$**	**$C_{Li\text{-}parental\ liquids}$**	**$C_{Li\text{-}liquids}$**	**f_2**	**$\delta^7Li_{parental\text{-}liquids}$**	**$\alpha_{ol:cpx=3:2}$**	**$\delta^7Li_{liquids}$**
1	0.3	45.79	45.79	1	9.16	0.99	9.16
0.95	0.3	45.79	47.44	0.98	9.16	0.99	9.29
0.9	0.3	47.44	51	0.97	9.29	0.99	9.55
0.85	0.3	51	56.97	0.95	9.55	0.99	9.97
0.8	0.3	56.97	66.21	0.93	9.97	0.99	10.55
**Source-II: α = 0.994**							
Stage 1: olivine fractionation							
**F_1**	**D_{ol}**	**$C_{Li\text{-}parental\ liquids}$**	**$C_{Li\text{-}liquids}$**	**f_1**	**$\delta^7Li_{parental\text{-}liquids}$**	**α_{ol}**	**$\delta 7^{Li}_{liquids}$**
1	0.3	41.09	41.09	1	8.77	0.99	8.77
0.95	0.3	41.09	42.58	0.98	8.77	0.99	8.86
0.9	0.3	42.58	45.79	0.97	8.86	0.99	9.06
Stage 2: olivine and clinopyroxene fractionation (D_{Li} = 0.3)							
**F_2**	**$D_{ol:cpx=3:2}$**	**$C_{Li\text{-}parental\ liquids}$**	**$C_{Li\text{-}liquids}$**	**f_2**	**$\delta^7Li_{parental\text{-}liquids}$**	**$\alpha_{ol:cpx=3:2}$**	**$\delta^7Li_{liquids}$**
1	0.3	45.79	45.79	1	9.06	0.99	9.06
0.95	0.3	45.79	47.44	0.98	9.06	0.99	9.16
0.9	0.3	47.44	51	0.97	9.16	0.99	9.36
0.85	0.3	51	56.97	0.95	9.36	0.99	9.67

0.8	0.3	56.97	66.21	0.93	9.67	0.99	10.11
**Source-II: α = 0.996**							
Stage 1: olivine fractionation							
**F_1**	**D_{ol}**	**$C_{Li\text{-}parental\ liquids}$**	**$C_{Li\text{-}liquids}$**	**f_1**	**$\delta^7Li_{parental\text{-}liquids}$**	**α_{ol}**	**$\delta 7^{Li}_{liquids}$**
1	0.3	41.09	41.09	1	8.77	1	8.77
0.95	0.3	41.09	42.58	0.98	8.77	1	8.83
0.9	0.3	42.58	45.79	0.97	8.83	1	8.96
Stage 2: olivine and clinopyroxene fractionation (D_{Li} = 0.3)							
**F_2**	**$D_{ol:cpx=3:2}$**	**$C_{Li\text{-}parental\ liquids}$**	**$C_{Li\text{-}liquids}$**	**f_2**	**$\delta^7Li_{parental\text{-}liquids}$**	**$\alpha_{ol:cpx=3:2}$**	**$\delta^7Li_{liquids}$**
1	0.3	45.79	45.79	1	8.96	1	8.96
0.95	0.3	45.79	47.44	0.98	8.96	1	9.03
0.9	0.3	47.44	51	0.97	9.03	1	9.16
0.85	0.3	51	56.97	0.95	9.16	1	9.37
0.8	0.3	56.97	66.21	0.93	9.37	1	9.66
**Source-II: α = 0.998**							
Stage 1: olivine fractionation							
**F_1**	**D_{ol}**	**$C_{Li\text{-}parental\ liquids}$**	**$C_{Li\text{-}liquids}$**	**f_1**	**$\delta^7Li_{parental\text{-}liquids}$**	**α_{ol}**	**$\delta 7^{Li}_{liquids}$**
1	0.3	41.09	41.09	1	8.77	1	8.77
0.95	0.3	41.09	42.58	0.98	8.77	1	8.8
0.9	0.3	42.58	45.79	0.97	8.8	1	8.87
Stage 2: olivine and clinopyroxene fractionation (D_{Li} = 0.3)							
**F_2**	**$D_{ol:cpx=3:2}$**	**$C_{Li\text{-}parental\ liquids}$**	**$C_{Li\text{-}liquids}$**	**f_2**	**$\delta^7Li_{parental\text{-}liquids}$**	**$\alpha_{ol:cpx=3:2}$**	**$\delta^7Li_{liquids}$**
1	0.3	45.79	45.79	1	8.87	1	8.87
0.95	0.3	45.79	47.44	0.98	8.87	1	8.9
0.9	0.3	47.44	51	0.97	8.9	1	8.96
0.85	0.3	51	56.97	0.95	8.96	1	9.07
0.8	0.3	56.97	66.21	0.93	9.07	1	9.21
**Source-I: α = 0.992**							
Stage 1: olivine fractionation							
**F_1**	**D_{ol}**	**$C_{Li\text{-}parental\ liquids}$**	**$C_{Li\text{-}liquids}$**	**f_1**	**$\delta^7Li_{parental\text{-}liquids}$**	**α_{ol}**	**$\delta 7^{Li}_{liquids}$**
1	0.3	41.09	41.09	1	3.5	0.99	3.5
0.95	0.3	41.09	42.58	0.98	3.5	0.99	3.63
0.9	0.3	42.58	45.79	0.97	3.63	0.99	3.89
Stage 2: olivine and clinopyroxene fractionation (D_{Li} = 0.3)							
**F_2**	**$D_{ol:cpx=3:2}$**	**$C_{Li\text{-}parental\ liquids}$**	**$C_{Li\text{-}liquids}$**	**f_2**	**$\delta^7Li_{parental\text{-}liquids}$**	**$\alpha_{ol:cpx=3:2}$**	**$\delta^7Li_{liquids}$**
1	0.3	45.79	45.79	1	3.89	0.99	3.89
0.95	0.3	45.79	47.44	0.98	3.89	0.99	4.02
0.9	0.3	47.44	51	0.97	4.02	0.99	4.28
0.85	0.3	51	56.97	0.95	4.28	0.99	4.7
0.8	0.3	56.97	66.21	0.93	4.7	0.99	5.28
**Source-I: α = 0.994**							
Stage 1: olivine fractionation							
**F_1**	**D_{ol}**	**$C_{Li\text{-}parental\ liquids}$**	**$C_{Li\text{-}liquids}$**	**f_1**	**$\delta^7Li_{parental\text{-}liquids}$**	**α_{ol}**	**$\delta 7^{Li}_{liquids}$**

1	0.3	41.09	41.09	1	3.5	0.99	3.5
0.95	0.3	41.09	42.58	0.98	3.5	0.99	3.59
0.9	0.3	42.58	45.79	0.97	3.59	0.99	3.79
Stage 2: olivine and clinopyroxene fractionation (D_{Li} = 0.3)							
**F_2**	**$D_{ol:cpx=3:2}$**	**$C_{Li\text{-}parental\ liquids}$**	**$C_{Li\text{-}liquids}$**	**f_2**	**$\delta^7Li_{parental\text{-}liquids}$**	**$\alpha_{ol:cpx=3:2}$**	**$\delta^7Li_{liquids}$**
1	0.3	45.79	45.79	1	3.79	0.99	3.79
0.95	0.3	45.79	47.44	0.98	3.79	0.99	3.89
0.9	0.3	47.44	51	0.97	3.89	0.99	4.09
0.85	0.3	51	56.97	0.95	4.09	0.99	4.4
0.8	0.3	56.97	66.21	0.93	4.4	0.99	4.84
**Source-I: α = 0.996**							
Stage 1: olivine fractionation							
**F_1**	**D_{ol}**	**$C_{Li\text{-}parental\ liquids}$**	**$C_{Li\text{-}liquids}$**	**f_1**	**$\delta^7Li_{parental\text{-}liquids}$**	**α_{ol}**	**$\delta7^{Li}_{liquids}$**
1	0.3	41.09	41.09	1	3.5	1	3.5
0.95	0.3	41.09	42.58	0.98	3.5	1	3.56
0.9	0.3	42.58	45.79	0.97	3.56	1	3.69
Stage 2: olivine and clinopyroxene fractionation (D_{Li} = 0.3)							
**F_2**	**$D_{ol:cpx=3:2}$**	**$C_{Li\text{-}parental\ liquids}$**	**$C_{Li\text{-}liquids}$**	**f_2**	**$\delta^7Li_{parental\text{-}liquids}$**	**$\alpha_{ol:cpx=3:2}$**	**$\delta^7Li_{liquids}$**
1	0.3	45.79	45.79	1	3.69	1	3.69
0.95	0.3	45.79	47.44	0.98	3.69	1	3.76
0.9	0.3	47.44	51	0.97	3.76	1	3.89
0.85	0.3	51	56.97	0.95	3.89	1	4.1
0.8	0.3	56.97	66.21	0.93	4.1	1	4.39
**Source-I: α = 0.998**							
Stage 1: olivine fractionation							
**F_1**	**D_{ol}**	**$C_{Li\text{-}parental\ liquids}$**	**$C_{Li\text{-}liquids}$**	**f_1**	**$\delta^7Li_{parental\text{-}liquids}$**	**α_{ol}**	**$\delta7^{Li}_{liquids}$**
1	0.3	41.09	41.09	1	3.5	1	3.5
0.95	0.3	41.09	42.58	0.98	3.5	1	3.53
0.9	0.3	42.58	45.79	0.97	3.53	1	3.6
Stage 2: olivine and clinopyroxene fractionation (D_{Li} = 0.3)							
**F_2**	**$D_{ol:cpx=3:2}$**	**$C_{Li\text{-}parental\ liquids}$**	**$C_{Li\text{-}liquids}$**	**f_2**	**$\delta^7Li_{parental\text{-}liquids}$**	**$\alpha_{ol:cpx=3:2}$**	**$\delta^7Li_{liquids}$**
1	0.3	45.79	45.79	1	3.6	1	3.6
0.95	0.3	45.79	47.44	0.98	3.6	1	3.63
0.9	0.3	47.44	51	0.97	3.63	1	3.69
0.85	0.3	51	56.97	0.95	3.69	1	3.8
0.8	0.3	56.97	66.21	0.93	3.8	1	3.94

Table S5.

Results of the modeling of the Li contents (ppm) and Li isotope compositions (‰) during crystal fractionation.

F	D	$C_{Li\text{-}parental\ liquids}$	$C_{Li\text{-}liquids}$	f	$\delta^7Li_{parental\text{-}liquids}$	α	$\delta^7Li_{liquids}$
**Melt derived from a mantle without assimilation**							

1	0.3	1.2	1.2	1	3.5	0.994	3.50
0.95	0.3	2.2	2.28	0.98	3.5	0.994	3.59
0.9	0.3	3.2	3.44	0.97	3.5	0.994	3.70
0.85	0.3	4.2	4.69	0.95	3.5	0.994	3.81
0.8	0.3	5.2	6.05	0.93	3.5	0.994	3.94
**Melt derived from a mantle mixing with 2% sediments**							
1	0.3	2.85	2.85	1	16.12	0.994	16.12
0.95	0.3	3.85	3.99	0.98	16.12	0.994	16.22
0.9	0.3	4.85	5.21	0.97	16.12	0.994	16.32
0.85	0.3	5.85	6.53	0.95	16.12	0.994	16.44
0.8	0.3	6.85	7.96	0.93	16.12	0.994	16.56
**Melt derived from a mantle mixing with 4% sediments**							
1	0.3	4.5	4.5	1	19.49	0.994	19.49
0.95	0.3	5.5	5.7	0.98	19.49	0.994	19.59
0.9	0.3	6.5	6.98	0.97	19.49	0.994	19.69
0.85	0.3	7.5	8.38	0.95	19.49	0.994	19.81
0.8	0.3	8.5	9.88	0.93	19.49	0.994	19.93
**Melt derived from a mantle mixing with 6% sediments**							
1	0.3	6.14	6.14	1	21.05	0.994	21.05
0.95	0.3	7.14	7.4	0.98	21.05	0.994	21.15
0.9	0.3	8.14	8.76	0.97	21.05	0.994	21.25
0.85	0.3	9.14	10.22	0.95	21.05	0.994	21.37
0.8	0.3	10.14	11.8	0.93	21.05	0.994	21.50
**Melt derived from a mantle mixing with 8% sediments**							
1	0.3	7.79	7.79	1	21.95	0.994	21.95
0.95	0.3	8.79	9.11	0.98	21.95	0.994	22.05
0.9	0.3	9.79	10.53	0.97	21.95	0.994	22.15
0.85	0.3	10.79	12.06	0.95	21.95	0.994	22.27
0.8	0.3	11.79	13.71	0.93	21.95	0.994	22.40
**Melt derived from a mantle mixing with 10% sediments**							
1	0.3	9.44	9.44	1	22.54	0.994	22.54
0.95	0.3	10.44	10.82	0.98	22.54	0.994	22.64
0.9	0.3	11.44	12.3	0.97	22.54	0.994	22.74
0.85	0.3	12.44	13.9	0.95	22.54	0.994	22.86
0.8	0.3	13.44	15.63	0.93	22.54	0.994	22.98

Table S6.

Au reserves of representative Au deposits in Jiaodong.

Deposit	Region	Latitude	Longitude	Developed	Res (t)
Shaling	Jiaobei terrane	37.38	120.02	No	389
Sanshandao	Jiaobei terrane	37.41	119.96	Yes	200
Linglong	Jiaobei terrane	37.45	120.48	Yes	166
Dayingezhuang	Jiaobei terrane	37.22	120.35	Yes	125
Jiaojia	Jiaobei terrane	37.39–37.41	120.11–120.13	Yes	56.99

Xincheng	Jiaobei terrane	37.43–37.45	120.12–120.15	Yes	200
Wang'ershan	Jiaobei terrane	37.38–37.43	120.12–120.17	Yes	>60
Xiadian	Jiaobei terrane	37.19	120.39	Yes	77.57
Sanjia	Sulu terrane	37.63	120.95	Yes	2.02
Yinggezhuang	Sulu terrane	37.04–37.05	121.56–121.57	Yes	9
Tangjiagou	Sulu terrane	37.09	121,53	Yes	16.4
Rushan	Sulu terrane	37,1	121,63	Yes	>30

Table S7

Calculation results of oxygen fugacity for Jiaodong lamprophyres.

	Amphibole Oxybarometer														
Label	LK3C-5	LK6A-2	LK6A-3	LK6A-4	LK6B-1	LK6B-2	LK6B-3	LK6B-4	LK6C-1	LK6C-2	LK6C-3	LK6C-4	LK6D-1	LK6D-2	LK6D-3
Stage 1: Amphibole compositions															
SiO_2(wt%)	38.87	41.91	39.97	40.98	40.80	40.47	40.74	40.48	40.52	40.70	41.30	40.42	41.07	40.01	40.20
TiO_2	2.84	3.94	3.89	3.06	3.06	3.72	3.68	3.56	3.54	3.77	2.93	3.72	3.36	3.83	3.61
Al_2O_3	14.62	9.25	12.51	12.13	12.31	12.18	12.34	12.53	12.18	12.11	12.01	12.09	12.16	12.53	12.31
Cr_2O_3	0.00	0.00	0.02	0.07	0.03	0.00	0.00	0.00	0.00	0.05	0.08	0.00	0.10	0.00	0.00
FeO_{tot}	13.70	13.88	12.85	12.68	12.70	12.43	11.09	12.58	12.56	12.80	12.92	12.83	11.90	12.59	12.56
MnO	0.15	0.21	0.08	0.16	0.22	0.13	0.10	0.10	0.25	0.16	0.09	0.12	0.10	0.22	0.15
NiO+ZnO	0.00	0.06	0.00	0.04	0.03	0.00	0.10	0.05	0.15	0.03	0.00	0.00	0.00	0.09	0.00
MgO	11.76	14.17	12.63	12.76	12.92	12.89	13.30	13.07	12.95	13.06	13.24	12.94	13.70	12.74	12.85
CaO	12.14	9.80	11.82	11.63	11.63	11.72	11.86	11.69	11.61	11.63	11.59	11.77	11.61	11.87	11.58
Na_2O	2.18	2.12	2.37	2.34	2.43	2.43	2.33	2.43	2.44	2.39	2.37	2.36	2.16	2.34	2.42
K_2O	1.55	1.07	1.13	1.05	1.19	1.14	1.12	1.13	1.18	1.07	1.07	1.13	1.04	1.08	1.15
F	0.00	0.00	0.00	0.00	0.00	0.00	0.00	0.00	0.00	0.00	0.00	0.00	0.00	0.00	0.00
Cl	0.00	0.00	0.00	0.00	0.00	0.00	0.00	0.00	0.00	0.00	0.00	0.00	0.00	0.00	0.00
Stage 2: Initial total re-calculation															
SiO_2(wt%)	39.01	42.72	40.38	41.50	41.14	40.94	41.40	40.73	40.87	40.91	41.52	40.78	41.50	40.41	40.77
TiO_2	2.85	4.02	3.93	3.10	3.09	3.76	3.74	3.58	3.57	3.79	2.95	3.75	3.39	3.87	3.66
Al_2O_3	14.67	9.43	12.64	12.28	12.41	12.32	12.54	12.61	12.29	12.17	12.07	12.20	12.29	12.66	12.49
Cr_2O_3	0.00	0.00	0.02	0.07	0.03	0.00	0.00	0.00	0.00	0.05	0.08	0.00	0.10	0.00	0.00
FeO_{tot}	13.75	14.15	12.98	12.84	12.81	12.57	11.27	12.66	12.67	12.87	12.99	12.94	12.02	12.72	12.74
MnO	0.15	0.21	0.08	0.16	0.22	0.13	0.10	0.10	0.25	0.16	0.09	0.12	0.10	0.22	0.15
NiO+ZnO	0.00	0.06	0.00	0.04	0.03	0.00	0.10	0.05	0.15	0.03	0.00	0.00	0.00	0.09	0.00
MgO	11.80	14.44	12.76	12.92	13.03	13.04	13.52	13.15	13.06	13.13	13.31	13.05	13.84	12.87	13.03
CaO	12.18	9.99	11.94	11.78	11.73	11.86	12.05	11.76	11.71	11.69	11.65	11.87	11.73	11.99	11.74
Na_2O	2.19	2.16	2.39	2.37	2.45	2.46	2.37	2.45	2.46	2.40	2.38	2.38	2.18	2.36	2.45
K_2O	1.56	1.09	1.14	1.06	1.20	1.15	1.14	1.14	1.19	1.08	1.08	1.14	1.05	1.09	1.17
F	0.00	0.00	0.00	0.00	0.00	0.00	0.00	0.00	0.00	0.00	0.00	0.00	0.00	0.00	0.00
Cl	0.00	0.00	0.00	0.00	0.00	0.00	0.00	0.00	0.00	0.00	0.00	0.00	0.00	0.00	0.00
Sum	98.15	98.27	98.28	98.12	98.13	98.23	98.22	98.23	98.23	98.27	98.11	98.24	98.21	98.28	98.21
Fe_2O_3	5.43	3.82	3.68	3.62	3.49	3.51	3.50	3.54	3.26	3.28	3.73	3.56	3.49	3.27	3.72
FeO	8.86	10.71	9.67	9.58	9.67	9.42	8.12	9.48	9.73	9.92	9.64	9.74	8.88	9.77	9.39

(Continued)															
O=F,Cl	0.00	0.00	0.00	0.00	0.00	0.00	0.00	0.00	0.00	0.00	0.00	0.00	0.00	0.00	0.00
H_2O	1.42	1.32	1.32	1.50	1.51	1.39	1.40	1.38	1.43	1.37	1.50	1.37	1.41	1.36	1.39
TEO	100.12	99.96	99.97	99.98	99.99	99.97	99.97	99.97	99.98	99.97	99.98	99.97	99.97	99.97	99.97
Stage 3: AMFORM formula															
Mg(apfu)	2.632	3.208	2.842	2.861	2.890	2.895	2.980	2.921	2.904	2.918	2.947	2.904	3.055	2.865	2.895
Si	5.836	6.365	6.033	6.163	6.122	6.097	6.124	6.068	6.096	6.101	6.167	6.085	6.144	6.035	6.074
Al	0.423	0.021	0.259	0.313	0.299	0.259	0.310	0.281	0.255	0.240	0.281	0.230	0.288	0.263	0.267
Ti	0.321	0.450	0.442	0.346	0.345	0.422	0.416	0.401	0.401	0.425	0.329	0.421	0.378	0.435	0.410
Fe^{3+}	0.612	0.429	0.414	0.405	0.390	0.393	0.389	0.397	0.366	0.368	0.416	0.400	0.389	0.368	0.417
Fe^{2+}	1.108	1.334	1.208	1.190	1.203	1.173	1.005	1.180	1.214	1.237	1.197	1.215	1.100	1.220	1.170
Ca	0.069	0.072	0.089	0.023	0.032	0.050	0.036	0.077	0.062	0.086	0.046	0.085	0.096	0.108	0.053
Na	0.635	0.624	0.694	0.682	0.707	0.710	0.679	0.706	0.712	0.695	0.686	0.689	0.627	0.684	0.709
K	0.297	0.207	0.218	0.201	0.228	0.219	0.215	0.216	0.226	0.205	0.204	0.217	0.198	0.208	0.222
Stage 4: Calculated Mg, △NNO and △FMQ*															
Mg*	2.472	2.924	2.482	2.622	2.645	2.547	2.612	2.597	2.584	2.575	2.736	2.575	2.771	2.505	2.566
△NNO	0.054	0.797	0.071	0.300	0.338	0.176	0.284	0.260	0.238	0.224	0.488	0.223	0.546	0.109	0.208
△FMQ	0.754	1.497	0.771	1.000	1.038	0.876	0.984	0.960	0.938	0.924	1.188	0.923	1.246	0.809	0.908

Table S7*(Continued)*

	Amphibole Oxybarometer														
Label	LK6D-4	LK6E-1	LK6E-2	LK6E-3	LK6E-4	LK6F-1	LK6F-2	ZYL7E-6	PC2C-2	PC2C-3	PC2C-4	PC2C-5	PC2F-4	PC2D-8	RS1B-1
Stage 1:Amphibole compositions															
SiO_2(wt%)	40.85	41.19	41.38	41.25	40.84	40.69	40.69	40.59	53.88	53.87	39.68	38.31	54.57	38.42	39.03
TiO_2	3.64	2.86	2.79	2.85	3.59	3.31	3.66	3.54	0.09	0.26	2.89	4.05	0.11	4.03	2.95
Al_2O_3	12.26	11.89	11.66	11.83	12.11	12.15	12.02	12.41	2.57	2.18	13.68	14.50	1.90	14.76	15.16
Cr_2O_3	0.09	0.03	0.01	0.05	0.00	0.00	0.02	0.00	0.07	0.27	0.04	0.00	0.15	0.00	0.03
FeO_{tot}	12.77	12.85	12.88	12.70	12.54	12.50	12.91	12.64	11.06	10.75	14.27	14.44	10.17	13.81	13.97
MnO	0.14	0.14	0.25	0.17	0.19	0.22	0.21	0.22	0.32	0.37	0.26	0.14	0.32	0.19	0.05
NiO+ZnO	0.12	0.05	0.00	0.00	0.00	0.01	0.00	0.07	0.11	0.05	0.05	0.00	0.01	0.05	0.03
MgO	12.70	13.00	13.18	13.25	13.26	13.07	13.02	12.75	16.29	16.66	11.51	10.73	16.72	10.56	11.82
CaO	11.52	11.56	11.57	11.50	11.69	11.52	11.42	11.49	12.58	12.61	11.72	11.85	12.81	11.96	10.65
Na_2O	2.51	2.49	2.39	2.33	2.37	2.44	2.34	2.36	0.53	0.51	2.11	2.02	0.37	2.07	2.48
K_2O	1.18	1.13	1.14	1.06	1.10	1.15	1.11	1.43	0.13	0.10	1.78	1.92	0.05	1.91	1.31
F	0.00	0.00	0.00	0.00	0.00	0.00	0.00	0.00	0.00	0.00	0.00	0.00	0.00	0.00	0.00
Cl	0.00	0.00	0.00	0.00	0.00	0.00	0.00	0.00	0.00	0.00	0.00	0.00	0.00	0.00	0.00
Stage 2: Initial total re-calculation															
SiO_2(wt%)	41.05	41.56	41.73	41.71	41.07	41.15	41.04	40.90	53.84	53.85	39.75	38.48	54.78	38.67	39.32
TiO_2	3.66	2.89	2.81	2.88	3.61	3.35	3.69	3.57	0.09	0.26	2.90	4.07	0.11	4.06	2.97
Al_2O_3	12.32	12.00	11.76	11.96	12.18	12.29	12.12	12.50	2.57	2.18	13.70	14.56	1.91	14.85	15.27
Cr_2O_3	0.09	0.03	0.01	0.05	0.00	0.00	0.02	0.00	0.07	0.27	0.04	0.00	0.15	0.00	0.03
FeO_{tot}	12.83	12.97	12.99	12.84	12.61	12.64	13.02	12.74	11.05	10.75	14.30	14.50	10.21	13.90	14.07
MnO	0.14	0.14	0.25	0.17	0.19	0.22	0.21	0.22	0.32	0.37	0.26	0.14	0.32	0.19	0.05
NiO+ZnO	0.12	0.05	0.00	0.00	0.00	0.01	0.00	0.07	0.11	0.05	0.05	0.00	0.01	0.05	0.03
MgO	12.76	13.12	13.29	13.40	13.33	13.22	13.13	12.85	16.28	16.65	11.53	10.78	16.78	10.63	11.91
CaO	11.58	11.67	11.67	11.63	11.75	11.65	11.52	11.58	12.57	12.61	11.74	11.90	12.86	12.04	10.73
Na_2O	2.52	2.51	2.41	2.36	2.38	2.47	2.36	2.38	0.53	0.51	2.11	2.03	0.37	2.08	2.50
K_2O	1.19	1.14	1.15	1.07	1.11	1.16	1.12	1.44	0.13	0.10	1.78	1.93	0.05	1.92	1.32
F	0.00	0.00	0.00	0.00	0.00	0.00	0.00	0.00	0.00	0.00	0.00	0.00	0.00	0.00	0.00
Cl	0.00	0.00	0.00	0.00	0.00	0.00	0.00	0.00	0.00	0.00	0.00	0.00	0.00	0.00	0.00
Sum	98.25	98.07	98.06	98.08	98.23	98.16	98.25	98.24	97.56	97.60	98.17	98.39	97.55	98.39	98.21
Fe_2O_3	3.22	3.72	3.53	3.71	3.29	3.48	3.33	3.28	1.00	1.32	4.22	5.73	0.00	4.81	4.82
FeO	9.93	9.62	9.81	9.51	9.65	9.51	10.02	9.78	10.15	9.56	10.50	9.35	10.21	9.57	9.74
O=F,Cl	0.00	0.00	0.00	0.00	0.00	0.00	0.00	0.00	0.00	0.00	0.00	0.00	0.00	0.00	0.00
H_2O	1.40	1.54	1.58	1.54	1.41	1.47	1.39	1.41	2.10	2.10	1.44	1.20	2.05	1.23	1.34
TEO	99.97	99.99	99.99	99.99	99.98	99.98	99.97	99.98	99.76	99.83	100.03	100.16	99.61	100.10	100.03

(Continued)															
Stage 3: AMFORM formula															
Mg(apfu)	2.837	2.906	2.944	2.963	2.959	2.931	2.919	2.857	3.472	3.547	2.583	2.421	3.572	2.385	2.650
Si	6.122	6.177	6.200	6.188	6.113	6.121	6.119	6.101	7.703	7.693	5.974	5.799	7.820	5.821	5.869
Al	0.287	0.279	0.259	0.280	0.249	0.275	0.249	0.300	0.137	0.060	0.401	0.386	0.141	0.457	0.556
Ti	0.410	0.323	0.314	0.322	0.404	0.375	0.414	0.400	0.010	0.028	0.327	0.461	0.012	0.459	0.334
Fe^{3+}	0.361	0.416	0.395	0.414	0.369	0.390	0.374	0.368	0.108	0.142	0.477	0.650	0.000	0.545	0.541
Fe^{2+}	1.239	1.195	1.219	1.180	1.201	1.183	1.250	1.221	1.215	1.142	1.320	1.178	1.219	1.205	1.215
Ca	0.027	0.005	0.021	0.034	0.080	0.038	0.074	0.034	0.000	0.000	0.042	0.036	0.000	0.023	0.026
Na	0.729	0.724	0.694	0.678	0.688	0.712	0.682	0.688	0.074	0.071	0.616	0.593	0.070	0.608	0.723
K	0.226	0.216	0.218	0.203	0.210	0.221	0.213	0.274	0.024	0.018	0.342	0.371	0.009	0.369	0.251
Stage 4: Calculated Mg, △NNO and △FMQ*															
Mg*	2.500	2.694	2.753	2.762	2.639	2.644	2.599	2.549	3.847	3.902	2.437	2.130	3.918	2.061	2.426
△NNO	0.100	0.418	0.516	0.531	0.328	0.336	0.263	0.181	2.314	2.405	-0.004	-0.508	2.430	-0.622	-0.022
△FMQ	0.800	1.118	1.216	1.231	1.028	1.036	0.963	0.881	3.014	3.105	0.696	0.192	3.130	0.078	0.678

Table S7 *(Continued)*

Label	RS1B-2	RS1E-2	RS2A-1	RS2A-2	RS2A-3	RS2A-4	RS2B-1	RS2B-3	RS2B-4	RS2C-2	RS2D-2	RS2D-3	RS2D-4	RS2F-1	RS2F-2
	**Amphibole Oxybarometer**														
Stage 1: Amphibole compositions															
SiO_2(wt%)	40.09	40.19	39.80	40.70	40.09	39.43	39.82	39.86	39.45	40.12	39.77	39.52	39.85	41.50	40.09
TiO_2	2.61	2.82	3.98	2.65	3.59	3.70	3.22	2.98	3.98	3.21	3.32	2.94	3.12	1.87	3.27
Al_2O_3	14.34	15.15	13.11	12.61	13.20	13.32	13.04	13.12	13.53	12.85	12.97	12.99	12.99	11.71	12.98
Cr_2O_3	0.00	0.01	0.00	0.02	0.00	0.04	0.03	0.03	0.01	0.00	0.03	0.00	0.00	0.13	0.00
FeO_{tot}	13.41	11.44	11.73	12.78	12.15	12.78	12.89	13.02	11.24	12.72	13.11	13.32	13.07	13.89	12.90
MnO	0.16	0.14	0.10	0.17	0.10	0.11	0.08	0.08	0.08	0.09	0.12	0.21	0.15	0.33	0.17
NiO+ZnO	0.07	0.00	0.00	0.01	0.00	0.11	0.00	0.00	0.02	0.00	0.02	0.00	0.07	0.03	0.08
MgO	12.15	13.18	13.18	13.05	12.99	12.49	12.36	12.44	13.22	12.84	12.85	12.57	12.42	12.68	12.51
CaO	10.53	10.78	12.14	11.66	12.08	11.90	11.95	11.84	12.08	12.01	11.74	11.66	11.70	11.42	11.87
Na_2O	2.68	2.56	2.40	2.49	2.52	2.47	2.60	2.45	2.36	2.34	2.44	2.41	2.37	2.38	2.68
K_2O	1.17	1.33	1.14	1.10	1.16	1.18	1.31	1.20	1.16	1.23	1.17	1.30	1.25	1.18	1.25
F	0.00	0.00	0.00	0.00	0.00	0.00	0.00	0.00	0.00	0.00	0.00	0.00	0.00	0.00	0.00
Cl	0.00	0.00	0.00	0.00	0.00	0.00	0.00	0.00	0.00	0.00	0.00	0.00	0.00	0.00	0.00
Stage 2: Initial total re-calculation															
SiO_2(wt%)	40.46	40.43	40.10	41.04	40.24	39.73	40.17	40.31	39.93	40.43	40.04	40.00	40.33	41.83	40.24
TiO_2	2.63	2.84	4.01	2.67	3.60	3.73	3.25	3.01	4.03	3.23	3.34	2.98	3.16	1.89	3.28
Al_2O_3	14.47	15.24	13.21	12.71	13.25	13.42	13.15	13.27	13.70	12.95	13.06	13.15	13.15	11.80	13.03
Cr_2O_3	0.00	0.01	0.00	0.02	0.00	0.04	0.03	0.03	0.01	0.00	0.03	0.00	0.00	0.13	0.00
FeO_{tot}	13.53	11.51	11.82	12.89	12.19	12.88	13.00	13.17	11.38	12.82	13.20	13.48	13.23	14.00	12.95
MnO	0.16	0.14	0.10	0.17	0.10	0.11	0.08	0.08	0.08	0.09	0.12	0.21	0.15	0.33	0.17
NiO+ZnO	0.07	0.00	0.00	0.01	0.00	0.11	0.00	0.00	0.02	0.00	0.02	0.00	0.07	0.03	0.08
MgO	12.26	13.26	13.28	13.16	13.04	12.59	12.47	12.58	13.38	12.94	12.94	12.72	12.57	12.78	12.56
CaO	10.63	10.84	12.23	11.76	12.12	11.99	12.05	11.97	12.23	12.10	11.82	11.80	11.84	11.51	11.91
Na_2O	2.70	2.58	2.42	2.51	2.53	2.49	2.62	2.48	2.39	2.36	2.46	2.44	2.40	2.40	2.69
K_2O	1.18	1.34	1.15	1.11	1.16	1.19	1.32	1.21	1.17	1.24	1.18	1.32	1.26	1.19	1.25
F	0.00	0.00	0.00	0.00	0.00	0.00	0.00	0.00	0.00	0.00	0.00	0.00	0.00	0.00	0.00
Cl	0.00	0.00	0.00	0.00	0.00	0.00	0.00	0.00	0.00	0.00	0.00	0.00	0.00	0.00	0.00
Sum	98.10	98.19	98.31	98.05	98.24	98.27	98.14	98.11	98.32	98.16	98.19	98.11	98.15	97.90	98.17
Fe_2O_3	3.88	3.51	4.36	3.83	4.34	5.03	4.95	4.09	4.42	3.93	4.98	5.08	3.90	3.81	4.89
FeO	10.04	8.35	7.89	9.44	8.29	8.36	8.55	9.48	7.40	9.28	8.71	8.91	9.71	10.58	8.55
O=F,Cl	0.00	0.00	0.00	0.00	0.00	0.00	0.00	0.00	0.00	0.00	0.00	0.00	0.00	0.00	0.00
H_2O	1.49	1.44	1.32	1.56	1.39	1.34	1.45	1.46	1.29	1.43	1.40	1.47	1.44	1.73	1.46
TEO	99.98	99.97	100.07	99.99	100.06	100.12	100.09	99.98	100.05	99.99	100.09	100.09	99.98	100.01	100.12

(Continued)															
Stage 3: AMFORM formula															
Mg(apfu)	2.717	2.916	2.941	2.915	2.888	2.798	2.770	2.797	2.958	2.875	2.875	2.831	2.798	2.837	2.789
Si	6.014	5.965	5.957	6.098	5.980	5.925	5.987	6.013	5.922	6.027	5.968	5.970	6.022	6.228	5.995
Al	0.550	0.615	0.269	0.324	0.300	0.284	0.297	0.345	0.316	0.302	0.262	0.282	0.335	0.299	0.282
Ti	0.295	0.315	0.448	0.299	0.403	0.418	0.364	0.338	0.449	0.363	0.375	0.334	0.355	0.211	0.368
Fe^{3+}	0.434	0.389	0.487	0.428	0.485	0.564	0.555	0.460	0.493	0.441	0.559	0.570	0.439	0.427	0.548
Fe^{2+}	1.248	1.031	0.981	1.173	1.030	1.042	1.065	1.183	0.918	1.157	1.086	1.113	1.213	1.317	1.065
Ca	0.000	0.000	0.086	0.036	0.050	0.054	0.000	0.050	0.091	0.083	0.066	0.044	0.062	0.000	0.000
Na	0.745	0.735	0.696	0.723	0.729	0.720	0.749	0.717	0.687	0.682	0.710	0.706	0.694	0.680	0.762
K	0.224	0.252	0.218	0.210	0.221	0.226	0.251	0.231	0.222	0.236	0.224	0.251	0.241	0.226	0.238
Stage 4: Calculated Mg, △NNO and △FMQ*															
Mg*	2.516	2.633	2.545	2.723	2.548	2.466	2.505	2.565	2.548	2.617	2.612	2.632	2.554	2.811	2.513
△NNO	0.126	0.318	0.174	0.467	0.179	0.043	0.108	0.207	0.179	0.293	0.284	0.317	0.188	0.611	0.121
△FMQ	0.826	1.018	0.874	1.167	0.879	0.743	0.808	0.907	0.879	0.993	0.984	1.017	0.888	1.311	0.821

Table S7 *(Continued)*

Label	Amphibole Oxybarometer														
	RS2F-3	RS2F-4	WD2A-1	WD2A-2	WD2B-1	WD2B-2	WD2B-3	WD2B-4	WD2B-5	WD2B-6	WD2E-1	WD2E-2	WD2E-3	WD2E-4	WD2F-1
Stage 1: Amphibole compositions															
SiO_2(wt%)	39.81	39.63	40.87	39.71	40.39	40.05	40.59	40.52	40.28	40.45	40.52	40.25	40.63	40.29	41.09
TiO_2	3.58	2.99	3.11	3.20	2.79	3.19	3.07	3.16	3.02	3.25	3.41	3.02	2.58	3.03	2.52
Al_2O_3	13.42	13.08	13.22	13.57	12.75	13.09	12.88	13.01	13.23	13.33	12.91	13.12	12.92	12.71	12.60
Cr_2O_3	0.02	0.00	0.02	0.04	0.00	0.10	0.13	0.00	0.04	0.08	0.12	0.00	0.05	0.01	0.06
FeO_{tot}	12.27	12.80	10.76	11.67	11.48	10.75	11.07	11.52	11.32	10.84	10.80	11.28	11.51	11.52	11.56
MnO	0.18	0.13	0.15	0.13	0.12	0.12	0.21	0.13	0.10	0.12	0.20	0.14	0.16	0.11	0.15
NiO+ZnO	0.06	0.00	0.00	0.00	0.05	0.05	0.03	0.00	0.06	0.04	0.03	0.00	0.04	0.13	0.00
MgO	12.63	12.82	13.74	13.43	13.96	13.74	13.77	13.46	13.79	13.95	13.92	13.80	13.68	13.73	14.25
CaO	11.95	11.69	11.96	11.98	11.82	12.14	11.86	12.00	12.01	12.03	12.22	12.00	11.83	11.78	11.90
Na_2O	2.45	2.44	2.46	2.42	2.34	2.44	2.46	2.29	2.46	2.40	2.36	2.38	2.42	2.35	2.36
K_2O	1.23	1.22	1.27	1.16	1.21	1.23	1.25	1.22	1.24	1.27	1.16	1.19	1.20	1.25	1.25
F	0.00	0.00	0.00	0.00	0.00	0.00	0.00	0.00	0.00	0.00	0.00	0.00	0.00	0.00	0.00
Cl	0.00	0.00	0.00	0.00	0.00	0.00	0.00	0.00	0.00	0.00	0.00	0.00	0.00	0.00	0.00
Stage 2: Initial total re-calculation															
SiO_2(wt%)	40.08	40.17	41.12	40.07	40.88	40.57	40.94	40.88	40.53	40.64	40.76	40.65	41.06	40.80	41.22
TiO_2	3.60	3.03	3.13	3.23	2.82	3.23	3.10	3.19	3.04	3.26	3.43	3.05	2.61	3.07	2.53
Al_2O_3	13.51	13.26	13.30	13.69	12.90	13.26	12.99	13.12	13.31	13.39	12.99	13.25	13.06	12.87	12.64
Cr_2O_3	0.02	0.00	0.02	0.04	0.00	0.10	0.13	0.00	0.04	0.08	0.12	0.00	0.05	0.01	0.06
FeO_{tot}	12.35	12.97	10.83	11.77	11.62	10.89	11.17	11.62	11.39	10.89	10.86	11.39	11.63	11.67	11.60
MnO	0.18	0.13	0.15	0.13	0.12	0.12	0.21	0.13	0.10	0.12	0.20	0.14	0.16	0.11	0.15
NiO+ZnO	0.06	0.00	0.00	0.00	0.05	0.05	0.03	0.00	0.06	0.04	0.03	0.00	0.04	0.13	0.00
MgO	12.71	12.99	13.82	13.55	14.13	13.92	13.89	13.58	13.87	14.01	14.00	13.94	13.83	13.90	14.30
CaO	12.03	11.85	12.03	12.09	11.96	12.30	11.96	12.11	12.08	12.09	12.29	12.12	11.96	11.93	11.94
Na_2O	2.47	2.47	2.48	2.44	2.37	2.47	2.48	2.31	2.48	2.41	2.37	2.40	2.45	2.38	2.37
K_2O	1.24	1.24	1.28	1.17	1.22	1.25	1.26	1.23	1.25	1.28	1.17	1.20	1.21	1.27	1.25
F	0.00	0.00	0.00	0.00	0.00	0.00	0.00	0.00	0.00	0.00	0.00	0.00	0.00	0.00	0.00
Cl	0.00	0.00	0.00	0.00	0.00	0.00	0.00	0.00	0.00	0.00	0.00	0.00	0.00	0.00	0.00
Sum	98.25	98.11	98.16	98.18	98.09	98.16	98.16	98.17	98.15	98.21	98.23	98.14	98.05	98.14	98.06
Fe_2O_3	4.06	4.80	3.27	4.75	4.05	4.31	3.53	3.55	4.63	4.05	3.40	4.00	3.78	4.13	3.71
FeO	8.70	8.65	7.88	7.50	7.97	7.01	7.98	8.43	7.22	7.24	7.80	7.79	8.23	7.95	8.26
O=F,Cl	0.00	0.00	0.00	0.00	0.00	0.00	0.00	0.00	0.00	0.00	0.00	0.00	0.00	0.00	0.00
H_2O	1.39	1.46	1.49	1.42	1.50	1.45	1.49	1.46	1.46	1.43	1.44	1.47	1.56	1.45	1.56
TEO	100.05	100.04	99.98	100.08	99.99	100.04	100.01	99.98	100.08	100.05	100.01	100.01	99.99	100.01	99.99

(Continued)

Stage 3: AMFORM formula															
Mg(apfu)	2.822	2.884	3.042	2.993	3.117	3.066	3.063	2.999	3.058	3.086	3.088	3.073	3.048	3.071	3.151
Si	5.967	5.980	6.070	5.936	6.050	5.996	6.056	6.056	5.992	6.002	6.030	6.013	6.073	6.046	6.096
Al	0.338	0.306	0.384	0.326	0.301	0.305	0.321	0.347	0.312	0.333	0.294	0.323	0.349	0.293	0.299
Ti	0.404	0.339	0.347	0.360	0.314	0.359	0.345	0.355	0.338	0.363	0.382	0.339	0.290	0.342	0.281
Fe^{3+}	0.455	0.538	0.363	0.529	0.451	0.479	0.393	0.396	0.515	0.451	0.379	0.446	0.421	0.461	0.413
Fe^{2+}	1.083	1.078	0.973	0.930	0.987	0.867	0.988	1.044	0.893	0.895	0.966	0.964	1.018	0.985	1.021
Ca	0.053	0.051	0.034	0.077	0.089	0.057	0.050	0.079	0.055	0.069	0.099	0.084	0.052	0.077	0.083
Na	0.712	0.714	0.708	0.701	0.680	0.708	0.712	0.664	0.710	0.690	0.681	0.689	0.701	0.684	0.679
K	0.235	0.235	0.241	0.221	0.231	0.235	0.238	0.233	0.235	0.240	0.220	0.227	0.229	0.239	0.237
Stage 4: Calculated Mg, △NNO and △FMQ*															
Mg*	2.486	2.656	2.733	2.706	2.893	2.758	2.773	2.718	2.791	2.774	2.750	2.803	2.842	2.814	2.969
△NNO	0.077	0.356	0.483	0.438	0.746	0.524	0.548	0.459	0.579	0.550	0.512	0.599	0.662	0.616	0.870
△FMQ	0.777	1.056	1.183	1.138	1.446	1.224	1.248	1.159	1.279	1.250	1.212	1.299	1.362	1.316	1.570

Table S7*(Continued)*

Label	Amphibole Oxybarometer														
	WD2F-2	WD2F-3	WD2F-4	WD2G-2	WD2G-3	WD2G-4	SSB5-1 Line 002	SSB5-1 Line 003	SSB5-1 Line 004	SSB5-1 Line 005	SSB5-1 Line 006	SSB5-1 Line 007	SSB5-1 Line 008	SSB5-1 Line 009	SSB5-1 Line 010
Stage 1: Amphibole compositions															
SiO_2(wt%)	40.50	40.66	39.92	40.63	42.57	40.44	41.20	41.40	40.60	39.90	41.00	40.80	40.60	41.70	41.50
TiO_2	3.22	3.47	3.45	2.73	0.63	1.82	2.41	2.37	2.06	2.11	2.07	2.21	2.20	2.28	2.30
Al_2O_3	12.94	12.42	13.14	12.71	11.71	12.87	13.00	13.10	13.10	13.60	13.00	13.60	13.50	12.80	12.80
Cr_2O_3	0.10	0.15	0.04	0.00	0.02	0.00	0.04	0.02	0.01	0.03	0.00	0.00	0.00	0.02	0.00
FeO_{tot}	10.99	10.78	10.98	11.70	12.98	13.52	11.40	11.10	13.40	14.70	13.40	13.10	12.40	10.90	11.40
MnO	0.11	0.09	0.13	0.19	0.32	0.20	0.11	0.12	0.17	0.19	0.15	0.14	0.13	0.11	0.12
NiO+ZnO	0.05	0.10	0.02	0.05	0.00	0.00	0.00	0.00	0.00	0.00	0.00	0.00	0.00	0.00	0.00
MgO	13.66	14.09	13.83	13.71	14.30	13.31	14.30	14.50	12.70	12.20	13.00	13.20	13.40	14.50	14.60
CaO	11.88	11.87	11.94	11.63	11.62	11.81	11.80	11.80	11.70	11.60	11.30	11.90	11.60	11.50	11.70
Na_2O	2.28	2.37	2.37	2.32	2.38	2.45	2.02	2.05	2.07	1.96	2.04	1.97	2.08	2.07	2.15
K_2O	1.29	1.28	1.24	1.20	0.99	1.11	1.48	1.61	1.61	1.66	1.59	1.67	1.60	1.52	1.42
F	0.00	0.00	0.00	0.00	0.00	0.00	0.00	0.00	0.00	0.00	0.00	0.00	0.00	0.00	0.00
Cl	0.00	0.00	0.00	0.00	0.00	0.00	0.00	0.00	0.00	0.00	0.00	0.00	0.00	0.00	0.00
Stage 2: Initial total re-calculation															
SiO_2(wt%)	40.99	41.06	40.40	41.14	42.64	40.60	41.34	41.40	40.84	39.93	41.19	40.58	40.82	41.98	41.52
TiO_2	3.26	3.50	3.49	2.76	0.63	1.83	2.42	2.37	2.07	2.11	2.08	2.20	2.21	2.30	2.30
Al_2O_3	13.10	12.54	13.30	12.87	11.73	12.92	13.04	13.10	13.18	13.61	13.06	13.53	13.57	12.88	12.81
Cr_2O_3	0.10	0.15	0.04	0.00	0.02	0.00	0.04	0.02	0.01	0.03	0.00	0.00	0.00	0.02	0.00
FeO_{tot}	11.12	10.89	11.11	11.85	13.00	13.57	11.44	11.10	13.48	14.71	13.46	13.03	12.47	10.97	11.41
MnO	0.11	0.09	0.13	0.19	0.32	0.20	0.11	0.12	0.17	0.19	0.15	0.14	0.13	0.11	0.12
NiO+ZnO	0.05	0.10	0.02	0.05	0.00	0.00	0.00	0.00	0.00	0.00	0.00	0.00	0.00	0.00	0.00
MgO	13.82	14.23	14.00	13.88	14.32	13.36	14.35	14.50	12.77	12.21	13.06	13.13	13.47	14.60	14.61
CaO	12.02	11.99	12.08	11.78	11.64	11.86	11.84	11.80	11.77	11.61	11.35	11.84	11.66	11.58	11.71
Na_2O	2.31	2.39	2.40	2.35	2.38	2.46	2.03	2.05	2.08	1.96	2.05	1.96	2.09	2.08	2.15
K_2O	1.31	1.29	1.25	1.21	0.99	1.11	1.48	1.61	1.62	1.66	1.60	1.66	1.61	1.53	1.42
F	0.00	0.00	0.00	0.00	0.00	0.00	0.00	0.00	0.00	0.00	0.00	0.00	0.00	0.00	0.00
Cl	0.00	0.00	0.00	0.00	0.00	0.00	0.00	0.00	0.00	0.00	0.00	0.00	0.00	0.00	0.00
Sum	98.19	98.24	98.22	98.08	97.67	97.91	98.08	98.08	97.99	98.03	98.01	98.05	98.04	98.05	98.05
Fe_2O_3	3.56	3.82	4.55	3.80	4.11	4.60	3.81	3.73	4.20	4.40	4.18	4.08	4.11	3.72	3.81
FeO	7.92	7.45	7.02	8.43	9.31	9.44	8.01	7.75	9.70	10.75	9.70	9.36	8.77	7.62	7.98
O=F,Cl	0.00	0.00	0.00	0.00	0.00	0.00	0.00	0.00	0.00	0.00	0.00	0.00	0.00	0.00	0.00
H_2O	1.43	1.40	1.39	1.53	1.95	1.66	1.53	1.54	1.58	1.52	1.57	1.53	1.54	1.57	1.56

(Continued)															
TEO	99.97	100.02	100.07	99.99	100.03	100.03	99.98	99.99	99.99	99.99	99.99	99.99	99.99	99.99	99.99
Stage 3: AMFORM formula															
Mg(apfu)	3.048	3.136	3.085	3.062	3.145	2.964	3.157	3.188	2.837	2.732	2.895	2.913	2.979	3.199	3.212
Si	6.062	6.072	5.973	6.088	6.281	6.041	6.102	6.106	6.084	5.993	6.124	6.040	6.054	6.170	6.124
Al	0.345	0.257	0.291	0.332	0.317	0.306	0.371	0.383	0.398	0.400	0.413	0.413	0.427	0.403	0.350
Ti	0.363	0.390	0.388	0.308	0.070	0.205	0.269	0.263	0.232	0.238	0.233	0.246	0.247	0.254	0.255
Fe^{3+}	0.396	0.425	0.506	0.423	0.455	0.515	0.423	0.414	0.471	0.497	0.467	0.457	0.458	0.412	0.423
Fe^{2+}	0.980	0.921	0.868	1.043	1.146	1.174	0.989	0.955	1.208	1.349	1.207	1.165	1.088	0.937	0.984
Ca	0.069	0.070	0.076	0.066	0.012	0.079	0.100	0.085	0.048	0.111	0.041	0.099	0.069	0.044	0.088
Na	0.662	0.686	0.688	0.674	0.681	0.710	0.580	0.586	0.601	0.571	0.591	0.565	0.601	0.594	0.615
K	0.246	0.244	0.237	0.229	0.186	0.212	0.280	0.303	0.308	0.318	0.303	0.315	0.304	0.287	0.267
Stage 4: Calculated Mg, △NNO and △FMQ*															
Mg*	2.749	2.799	2.754	2.850	3.272	2.922	3.019	3.047	2.795	2.723	2.854	2.850	2.886	3.063	3.079
△NNO	0.509	0.592	0.518	0.675	1.369	0.794	0.953	1.000	0.586	0.466	0.682	0.675	0.735	1.025	1.051
△FMQ	1.209	1.292	1.218	1.375	2.069	1.494	1.653	1.700	1.286	1.166	1.382	1.375	1.435	1.725	1.751

Table S7*(Continued)*

Label	Amphibole Oxybarometer														
	SSB5-1 Line 011	SSB5-1 Line 012	SSB5-1 Line 013	SSB5-1 Line 014	SSB5-1 Line 015	SSB5-1 Line 016	SSB5-1 Line 017	SSB5-1 Line 018	SSB5-1 Line 019	SSB5-1 Line 020	SSB5-1 Line 021	SSB5-1 Line 022	SSB5-1 Line 023	SSB5-1 Line 024	SSB5-1 Line 025
Stage 1: Amphibole compositions															
SiO_2(wt%)	42.00	41.80	42.10	42.10	41.80	41.60	42.00	42.00	42.70	42.50	42.60	42.70	42.50	42.20	42.00
TiO_2	2.26	2.24	2.16	2.15	2.19	2.08	1.99	2.10	2.13	2.19	2.22	2.18	2.08	2.25	2.24
Al_2O_3	12.80	12.60	12.70	12.60	12.50	12.30	12.50	12.20	11.70	12.20	11.80	11.80	12.20	12.20	12.50
Cr_2O_3	0.07	0.06	0.06	0.01	0.03	0.02	0.01	0.04	0.01	0.07	0.02	0.00	0.06	0.03	0.02
FeO_{tot}	10.80	10.60	10.60	11.20	11.60	11.80	11.80	10.70	10.80	10.70	10.90	10.50	10.50	11.50	11.80
MnO	0.11	0.09	0.11	0.10	0.10	0.10	0.14	0.12	0.12	0.12	0.10	0.13	0.10	0.11	0.12
NiO+ZnO	0.00	0.00	0.00	0.00	0.00	0.00	0.00	0.00	0.00	0.00	0.00	0.00	0.00	0.00	0.00
MgO	14.90	14.90	14.90	14.50	14.20	14.30	14.60	15.00	15.30	15.00	15.20	15.10	14.90	14.90	14.30
CaO	11.70	11.80	11.60	11.40	11.70	11.40	11.40	11.60	11.50	11.60	11.50	11.50	11.60	11.60	11.50
Na_2O	2.07	2.07	2.11	2.13	2.14	2.21	2.21	2.16	2.18	2.14	2.00	2.12	2.20	2.16	2.15
K_2O	1.56	1.56	1.53	1.50	1.44	1.40	1.35	1.44	1.37	1.51	1.42	1.40	1.45	1.40	1.31
F	0.00	0.00	0.00	0.00	0.00	0.00	0.00	0.00	0.00	0.00	0.00	0.00	0.00	0.00	0.00
Cl	0.00	0.00	0.00	0.00	0.00	0.00	0.00	0.00	0.00	0.00	0.00	0.00	0.00	0.00	0.00
Stage 2: Initial total re-calculation															
SiO_2(wt%)	41.91	41.94	42.17	42.25	41.93	41.93	41.99	42.27	42.78	42.50	42.71	42.95	42.67	42.06	42.04
TiO_2	2.26	2.25	2.16	2.16	2.20	2.10	1.99	2.11	2.13	2.19	2.23	2.19	2.09	2.24	2.24
Al_2O_3	12.77	12.64	12.72	12.64	12.54	12.40	12.50	12.28	11.72	12.20	11.83	11.87	12.25	12.16	12.51
Cr_2O_3	0.06	0.06	0.06	0.01	0.03	0.02	0.01	0.04	0.01	0.07	0.02	0.00	0.06	0.03	0.02
FeO_{tot}	10.78	10.63	10.62	11.24	11.64	11.89	11.80	10.77	10.82	10.70	10.93	10.56	10.54	11.46	11.81
MnO	0.10	0.09	0.11	0.10	0.10	0.10	0.13	0.12	0.12	0.12	0.10	0.13	0.10	0.11	0.12
NiO+ZnO	0.00	0.00	0.00	0.00	0.00	0.00	0.00	0.00	0.00	0.00	0.00	0.00	0.00	0.00	0.00
MgO	14.87	14.95	14.93	14.55	14.24	14.41	14.60	15.10	15.33	15.00	15.24	15.19	14.96	14.85	14.31
CaO	11.68	11.84	11.62	11.44	11.74	11.49	11.40	11.68	11.52	11.60	11.53	11.57	11.65	11.56	11.51
Na_2O	2.07	2.08	2.11	2.14	2.15	2.23	2.21	2.17	2.18	2.14	2.01	2.13	2.21	2.15	2.15
K_2O	1.56	1.57	1.53	1.51	1.44	1.41	1.35	1.45	1.37	1.51	1.42	1.41	1.46	1.40	1.31
F	0.00	0.00	0.00	0.00	0.00	0.00	0.00	0.00	0.00	0.00	0.00	0.00	0.00	0.00	0.00
Cl	0.00	0.00	0.00	0.00	0.00	0.00	0.00	0.00	0.00	0.00	0.00	0.00	0.00	0.00	0.00
Sum	98.06	98.04	98.03	98.02	98.01	97.98	97.98	97.99	97.99	98.02	98.03	98.00	97.99	98.03	98.03
Fe_2O_3	3.63	3.73	3.62	3.74	3.81	4.05	3.85	3.77	3.61	3.51	3.64	3.51	3.59	3.75	3.71
FeO	7.52	7.28	7.36	7.87	8.21	8.25	8.33	7.37	7.57	7.54	7.66	7.40	7.32	8.09	8.47
O=F,Cl	0.00	0.00	0.00	0.00	0.00	0.00	0.00	0.00	0.00	0.00	0.00	0.00	0.00	0.00	0.00
H_2O	1.57	1.58	1.60	1.60	1.60	1.61	1.63	1.62	1.64	1.62	1.60	1.64	1.65	1.59	1.59

(Continued)															
TEO	99.99	99.99	99.99	99.99	99.99	99.99	99.99	99.99	99.99	99.99	99.99	99.99	100.00	99.99	99.99
Stage 3: AMFORM formula															
Mg(apfu)	3.259	3.275	3.265	3.188	3.131	3.169	3.206	3.304	3.351	3.282	3.333	3.315	3.268	3.262	3.144
Si	6.162	6.164	6.189	6.209	6.183	6.183	6.186	6.206	6.273	6.237	6.267	6.288	6.252	6.196	6.195
Al	0.375	0.354	0.389	0.399	0.361	0.338	0.356	0.331	0.298	0.347	0.313	0.336	0.367	0.308	0.368
Ti	0.249	0.249	0.239	0.239	0.244	0.233	0.221	0.233	0.235	0.242	0.246	0.242	0.230	0.249	0.249
Fe^{3+}	0.401	0.413	0.400	0.414	0.423	0.450	0.427	0.417	0.398	0.388	0.402	0.387	0.395	0.416	0.412
Fe^{2+}	0.924	0.894	0.904	0.967	1.012	1.017	1.026	0.905	0.928	0.925	0.939	0.906	0.896	0.997	1.044
Ca	0.069	0.067	0.043	0.021	0.042	0.038	0.053	0.047	0.038	0.031	0.061	0.017	0.004	0.072	0.051
Na	0.589	0.592	0.601	0.609	0.614	0.637	0.631	0.619	0.621	0.609	0.570	0.605	0.627	0.615	0.615
K	0.292	0.293	0.287	0.282	0.272	0.265	0.254	0.271	0.257	0.283	0.266	0.263	0.272	0.262	0.246
Stage 4: Calculated Mg, △NNO and △FMQ*															
Mg*	3.127	3.144	3.138	3.075	3.022	3.076	3.123	3.188	3.234	3.155	3.223	3.187	3.144	3.144	3.028
△NNO	1.131	1.159	1.149	1.045	0.958	1.047	1.124	1.231	1.308	1.177	1.289	1.229	1.159	1.159	0.968
△FMQ	1.831	1.859	1.849	1.745	1.658	1.747	1.824	1.931	2.008	1.877	1.989	1.929	1.859	1.859	1.668

Table S7 *(Continued)*

Label	SSB5-1 Line 026	SSB5-1 Line 027	SSB5-1 Line 028	SSB5-1 Line 029	SSB5-1 Line 030	SSB5-1 Line 031	SSB5-1 Line 032	SSB5-1 Line 033	SSB5-1 Line 034	SSB5-1 Line 035	SSB5-1 Line 036	SSB5-1 Line 037	SSB5-1 Line 038	SSB5-1 Line 039	SSB5-2 Line 001
	**Amphibole Oxybarometer**														
Stage 1: Amphibole compositions															
SiO_2(wt%)	41.60	41.90	41.80	41.80	41.70	41.20	41.50	40.00	40.60	40.20	41.10	41.00	41.30	41.10	40.70
TiO_2	2.15	2.32	2.18	2.23	2.26	2.27	2.18	2.07	2.07	2.04	2.29	2.33	2.29	2.27	2.06
Al_2O_3	12.50	12.70	12.60	12.70	13.00	13.00	12.90	13.40	13.70	13.70	13.30	13.20	13.00	13.10	13.70
Cr_2O_3	0.00	0.03	0.10	0.05	0.05	0.01	0.07	0.02	0.03	0.02	0.03	0.02	0.05	0.02	0.00
FeO_{tot}	12.40	11.10	10.80	10.60	11.00	11.30	11.60	15.10	13.30	14.70	12.30	11.20	11.50	11.30	13.20
MnO	0.13	0.08	0.13	0.10	0.14	0.11	0.11	0.15	0.18	0.17	0.10	0.13	0.11	0.11	0.14
NiO+ZnO	0.00	0.00	0.00	0.00	0.00	0.00	0.00	0.00	0.00	0.00	0.00	0.00	0.00	0.00	0.00
MgO	13.90	14.80	14.70	14.80	14.40	14.20	14.20	12.10	12.20	12.00	13.70	14.20	14.00	14.30	13.20
CaO	11.50	11.70	11.70	11.80	11.70	11.60	11.60	11.50	11.00	11.40	11.60	11.70	11.70	11.70	11.80
Na_2O	2.13	2.20	2.05	2.16	2.01	2.07	2.13	2.14	2.00	1.97	2.01	2.03	2.14	1.99	2.08
K_2O	1.35	1.44	1.52	1.57	1.63	1.58	1.58	1.43	1.80	1.58	1.60	1.61	1.60	1.59	1.53
F	0.00	0.00	0.00	0.00	0.00	0.00	0.00	0.00	0.00	0.00	0.00	0.00	0.00	0.00	0.00
Cl	0.00	0.00	0.00	0.00	0.00	0.00	0.00	0.00	0.00	0.00	0.00	0.00	0.00	0.00	0.00
Stage 2: Initial total re-calculation															
SiO_2(wt%)	41.75	41.80	42.00	41.89	41.77	41.50	41.57	40.03	41.08	40.30	41.12	41.27	41.45	41.34	40.54
TiO_2	2.16	2.31	2.19	2.23	2.26	2.29	2.18	2.07	2.09	2.04	2.29	2.35	2.30	2.28	2.05
Al_2O_3	12.54	12.67	12.66	12.73	13.02	13.09	12.92	13.41	13.86	13.73	13.31	13.29	13.05	13.18	13.64
Cr_2O_3	0.00	0.03	0.10	0.05	0.05	0.01	0.07	0.02	0.03	0.02	0.03	0.02	0.05	0.02	0.00
FeO_{tot}	12.44	11.07	10.85	10.62	11.02	11.38	11.62	15.11	13.46	14.74	12.31	11.27	11.54	11.37	13.15
MnO	0.13	0.08	0.13	0.10	0.14	0.11	0.11	0.15	0.18	0.17	0.10	0.13	0.11	0.11	0.14
NiO+ZnO	0.00	0.00	0.00	0.00	0.00	0.00	0.00	0.00	0.00	0.00	0.00	0.00	0.00	0.00	0.00
MgO	13.95	14.77	14.77	14.83	14.43	14.30	14.22	12.11	12.34	12.03	13.71	14.29	14.05	14.38	13.15
CaO	11.54	11.67	11.75	11.83	11.72	11.68	11.62	11.51	11.13	11.43	11.60	11.78	11.74	11.77	11.75
Na_2O	2.14	2.19	2.06	2.16	2.01	2.09	2.13	2.14	2.02	1.97	2.01	2.04	2.15	2.00	2.07
K_2O	1.35	1.44	1.53	1.57	1.63	1.59	1.58	1.43	1.82	1.58	1.60	1.62	1.61	1.60	1.52
F	0.00	0.00	0.00	0.00	0.00	0.00	0.00	0.00	0.00	0.00	0.00	0.00	0.00	0.00	0.00
Cl	0.00	0.00	0.00	0.00	0.00	0.00	0.00	0.00	0.00	0.00	0.00	0.00	0.00	0.00	0.00
Sum	98.00	98.05	98.03	98.03	98.06	98.04	98.03	98.00	98.02	98.02	98.07	98.06	98.05	98.05	98.02
Fe_2O_3	3.95	3.73	3.67	3.68	3.62	3.92	3.84	4.53	4.18	4.36	3.97	3.84	3.82	3.92	4.16
FeO	8.89	7.72	7.55	7.31	7.77	7.85	8.17	11.03	9.70	10.81	8.74	7.82	8.10	7.84	9.40
O=F,Cl	0.00	0.00	0.00	0.00	0.00	0.00	0.00	0.00	0.00	0.00	0.00	0.00	0.00	0.00	0.00
H_2O	1.59	1.57	1.59	1.59	1.57	1.55	1.57	1.54	1.55	1.53	1.52	1.54	1.56	1.54	1.56

(Continued)

TEO	99.99	99.99	99.99	99.99	99.99	99.99	99.99	99.99	99.99	99.99	99.99	99.99	99.99	99.99	99.99
Stage 3: AMFORM formula															
Mg(apfu)	3.074	3.240	3.237	3.251	3.166	3.145	3.130	2.710	2.735	2.685	3.027	3.144	3.094	3.164	2.916
Si	6.172	6.153	6.175	6.159	6.150	6.120	6.136	6.009	6.106	6.033	6.091	6.090	6.122	6.100	6.030
Al	0.358	0.352	0.368	0.364	0.410	0.396	0.384	0.381	0.535	0.456	0.414	0.401	0.393	0.391	0.422
Ti	0.240	0.256	0.242	0.247	0.251	0.254	0.243	0.234	0.234	0.230	0.255	0.260	0.255	0.253	0.230
Fe^{3+}	0.439	0.413	0.406	0.407	0.401	0.436	0.426	0.512	0.467	0.492	0.442	0.427	0.425	0.435	0.466
Fe^{2+}	1.099	0.950	0.928	0.899	0.956	0.968	1.008	1.385	1.206	1.353	1.082	0.965	1.001	0.968	1.170
Ca	0.055	0.066	0.061	0.049	0.056	0.059	0.051	0.095	0.000	0.074	0.078	0.078	0.045	0.088	0.094
Na	0.613	0.626	0.587	0.617	0.575	0.596	0.611	0.623	0.559	0.573	0.578	0.585	0.615	0.573	0.598
K	0.256	0.270	0.286	0.295	0.307	0.299	0.298	0.274	0.345	0.302	0.302	0.305	0.303	0.301	0.289
Stage 4: Calculated Mg, △NNO and △FMQ*															
Mg*	2.989	3.095	3.118	3.111	3.042	3.021	3.023	2.697	2.696	2.680	2.928	3.011	2.965	3.047	2.862
△NNO	0.905	1.077	1.116	1.105	0.991	0.956	0.959	0.424	0.423	0.395	0.803	0.940	0.865	0.999	0.694
△FMQ	1.605	1.777	1.816	1.805	1.691	1.656	1.659	1.124	1.123	1.095	1.503	1.640	1.565	1.699	1.394

Table S7*(Continued)*

Label	Amphibole Oxybarometer														
	SSB5-2 Line 002	SSB5-2 Line 003	SSB5-2 Line 004	SSB5-2 Line 005	SSB5-2 Line 006	SSB5-2 Line 007	SSB5-2 Line 008	SSB5-2 Line 009	SSB5-2 Line 010	SSB5-2 Line 011	SSB5-2 Line 012	SSB5-2 Line 013	SSB5-2 Line 014	SSB5-2 Line 015	SSB5-2 Line 016
Stage 1: Amphibole compositions															
SiO_2(wt%)	41.20	41.30	41.50	41.40	41.70	40.40	40.20	40.60	40.80	41.20	41.00	41.20	41.50	41.20	41.20
TiO_2	2.18	2.25	2.28	2.13	2.28	2.24	1.95	2.13	1.85	2.09	2.30	2.26	2.28	2.28	2.22
Al_2O_3	13.20	13.20	12.90	13.10	13.00	14.00	14.00	13.50	13.40	13.20	13.20	13.30	13.00	12.90	13.00
Cr_2O_3	0.01	0.03	0.10	0.07	0.02	0.01	0.02	0.00	0.00	0.02	0.02	0.01	0.10	0.04	0.01
FeO_{tot}	12.50	12.30	11.10	11.20	11.60	13.70	14.20	13.40	12.90	12.10	11.90	11.00	10.10	11.20	11.80
MnO	0.13	0.11	0.10	0.11	0.09	0.19	0.16	0.15	0.14	0.11	0.12	0.10	0.07	0.12	0.11
NiO+ZnO	0.00	0.00	0.00	0.00	0.00	0.00	0.00	0.00	0.00	0.00	0.00	0.00	0.00	0.00	0.00
MgO	13.50	13.80	14.60	14.20	14.40	12.30	12.50	13.10	13.40	14.10	14.30	14.60	14.90	14.70	14.20
CaO	11.80	11.50	11.70	11.80	11.60	11.70	11.60	11.60	11.60	11.60	11.60	11.50	11.80	11.70	11.60
Na_2O	2.09	2.11	2.11	2.13	2.01	2.11	2.08	2.07	2.05	2.10	2.14	2.12	2.11	2.16	2.10
K_2O	1.49	1.54	1.60	1.57	1.52	1.52	1.52	1.55	1.53	1.57	1.56	1.58	1.59	1.62	1.48
F	0.00	0.00	0.00	0.00	0.00	0.00	0.00	0.00	0.00	0.00	0.00	0.00	0.00	0.00	0.00
Cl	0.00	0.00	0.00	0.00	0.00	0.00	0.00	0.00	0.00	0.00	0.00	0.00	0.00	0.00	0.00
Stage 2: Initial total re-calculation															
SiO_2(wt%)	41.17	41.26	41.53	41.53	41.63	40.35	40.11	40.57	40.93	41.17	40.97	41.36	41.76	41.25	41.33
TiO_2	2.18	2.25	2.28	2.14	2.28	2.24	1.95	2.13	1.86	2.09	2.30	2.27	2.29	2.28	2.23
Al_2O_3	13.19	13.19	12.91	13.14	12.98	13.98	13.97	13.49	13.44	13.19	13.19	13.35	13.08	12.92	13.04
Cr_2O_3	0.01	0.03	0.10	0.07	0.02	0.01	0.02	0.00	0.00	0.02	0.02	0.01	0.10	0.04	0.01
FeO_{tot}	12.49	12.29	11.11	11.24	11.58	13.68	14.17	13.39	12.94	12.09	11.89	11.04	10.16	11.21	11.84
MnO	0.13	0.11	0.10	0.11	0.09	0.19	0.16	0.15	0.14	0.11	0.12	0.10	0.07	0.12	0.11
NiO+ZnO	0.00	0.00	0.00	0.00	0.00	0.00	0.00	0.00	0.00	0.00	0.00	0.00	0.00	0.00	0.00
MgO	13.49	13.79	14.61	14.25	14.38	12.28	12.47	13.09	13.44	14.09	14.29	14.66	14.99	14.72	14.25
CaO	11.79	11.49	11.71	11.84	11.58	11.69	11.57	11.59	11.64	11.59	11.59	11.55	11.87	11.72	11.64
Na_2O	2.09	2.11	2.11	2.14	2.01	2.11	2.08	2.07	2.06	2.10	2.14	2.13	2.12	2.16	2.11
K_2O	1.49	1.54	1.60	1.58	1.52	1.52	1.52	1.55	1.53	1.57	1.56	1.59	1.60	1.62	1.48
F	0.00	0.00	0.00	0.00	0.00	0.00	0.00	0.00	0.00	0.00	0.00	0.00	0.00	0.00	0.00
Cl	0.00	0.00	0.00	0.00	0.00	0.00	0.00	0.00	0.00	0.00	0.00	0.00	0.00	0.00	0.00
Sum	98.03	98.05	98.06	98.02	98.06	98.05	98.00	98.03	97.97	98.02	98.06	98.06	98.05	98.05	98.04
Fe_2O_3	3.94	3.91	3.76	3.82	3.81	3.98	4.37	4.18	4.26	4.05	4.20	3.88	3.72	4.24	3.97
FeO	8.94	8.77	7.73	7.80	8.15	10.10	10.24	9.63	9.11	8.45	8.11	7.55	6.81	7.40	8.26
O=F,Cl	0.00	0.00	0.00	0.00	0.00	0.00	0.00	0.00	0.00	0.00	0.00	0.00	0.00	0.00	0.00
H_2O	1.57	1.54	1.55	1.59	1.54	1.54	1.55	1.54	1.60	1.56	1.53	1.54	1.56	1.55	1.55

(Continued)															
TEO	99.99	99.99	99.99	99.99	99.99	99.99	99.99	99.99	99.99	99.99	100.01	99.98	99.99	100.02	99.99
Stage 3: AMFORM formula															
Mg(apfu)	2.980	3.042	3.211	3.130	3.161	2.732	2.778	2.906	2.973	3.108	3.151	3.218	3.280	3.237	3.138
Si	6.102	6.108	6.123	6.122	6.140	6.019	5.993	6.042	6.073	6.091	6.060	6.092	6.129	6.086	6.107
Al	0.405	0.409	0.366	0.405	0.395	0.477	0.452	0.409	0.423	0.391	0.360	0.409	0.391	0.331	0.377
Ti	0.243	0.250	0.253	0.237	0.253	0.251	0.219	0.238	0.207	0.232	0.256	0.251	0.253	0.253	0.248
Fe^{3+}	0.440	0.436	0.417	0.424	0.423	0.447	0.491	0.468	0.475	0.451	0.467	0.430	0.411	0.471	0.442
Fe^{2+}	1.108	1.085	0.953	0.962	1.005	1.260	1.279	1.199	1.131	1.045	1.004	0.930	0.836	0.913	1.021
Ca	0.066	0.063	0.073	0.049	0.080	0.061	0.094	0.090	0.077	0.080	0.093	0.075	0.060	0.076	0.082
Na	0.600	0.605	0.604	0.611	0.574	0.610	0.601	0.597	0.592	0.602	0.613	0.608	0.604	0.619	0.603
K	0.282	0.291	0.301	0.296	0.285	0.289	0.289	0.294	0.291	0.296	0.294	0.298	0.300	0.305	0.280
Stage 4: Calculated Mg, △NNO and △FMQ*															
Mg*	2.893	2.939	3.080	3.018	3.048	2.653	2.761	2.850	2.947	3.027	3.034	3.081	3.123	3.110	3.029
△NNO	0.747	0.823	1.054	0.951	1.001	0.352	0.528	0.675	0.836	0.966	0.978	1.055	1.124	1.103	0.970
△FMQ	1.447	1.523	1.754	1.651	1.701	1.052	1.228	1.375	1.536	1.666	1.678	1.755	1.824	1.803	1.670

Table S7*(Continued)*

Label	SSB5-2 Line 017	SSB5-2 Line 018	SSB5-2 Line 019	SSB5-2 Line 020	SSB5-2 Line 021	SSB5-2 Line 023	SSB5-2 Line 024	SSB5-2 Line 027	SSB5-2 Line 028	SSB5-2 Line 029	SSB5-2 Line 030	SSB5-2 Line 031	SSB5-2 Line 032	SSB5-2 Line 033	SSB5-2 Line 034
	**Amphibole Oxybarometer**														
Stage 1: Amphibole compositions															
SiO_2(wt%)	41.50	41.00	41.00	40.70	41.30	40.60	41.20	41.40	41.40	41.00	40.70	41.10	41.30	41.10	41.50
TiO_2	2.28	2.30	2.06	2.05	2.15	2.36	2.30	2.33	2.12	2.13	2.08	2.14	2.36	2.37	2.30
Al_2O_3	12.80	13.00	13.20	13.50	13.10	13.10	13.20	13.30	13.20	13.30	13.30	13.00	12.90	12.90	13.00
Cr_2O_3	0.02	0.03	0.00	0.00	0.01	0.05	0.01	0.00	0.00	0.01	0.00	0.00	0.01	0.03	0.02
FeO_{tot}	11.30	12.00	12.80	13.00	12.30	11.80	12.20	11.60	12.20	13.20	13.20	12.50	11.40	11.70	11.10
MnO	0.10	0.12	0.17	0.16	0.13	0.13	0.13	0.11	0.12	0.14	0.18	0.11	0.10	0.09	0.10
NiO+ZnO	0.00	0.00	0.00	0.00	0.00	0.00	0.00	0.00	0.00	0.00	0.00	0.00	0.00	0.00	0.00
MgO	14.40	14.10	13.30	13.10	13.50	13.80	13.60	13.90	13.80	13.20	13.50	13.70	14.10	14.20	14.50
CaO	11.80	11.60	11.80	11.70	11.90	11.90	11.50	11.90	11.80	11.80	11.50	11.60	11.70	11.70	11.60
Na_2O	2.08	2.08	2.11	2.13	2.04	2.01	2.13	1.96	2.20	2.11	2.07	2.11	2.04	2.03	2.07
K_2O	1.49	1.43	1.41	1.39	1.49	1.61	1.63	1.69	1.33	1.38	1.42	1.43	1.53	1.48	1.53
F	0.00	0.00	0.00	0.00	0.00	0.00	0.00	0.00	0.00	0.00	0.00	0.00	0.00	0.00	0.00
Cl	0.00	0.00	0.00	0.00	0.00	0.00	0.00	0.00	0.00	0.00	0.00	0.00	0.00	0.00	0.00
Stage 2: Initial total re-calculation															
SiO_2(wt%)	41.61	41.17	41.06	40.81	41.34	40.89	41.27	41.35	41.33	40.89	40.73	41.23	41.56	41.29	41.64
TiO_2	2.29	2.31	2.06	2.06	2.15	2.38	2.30	2.33	2.12	2.12	2.08	2.15	2.37	2.38	2.31
Al_2O_3	12.83	13.05	13.22	13.54	13.11	13.19	13.22	13.28	13.18	13.27	13.31	13.04	12.98	12.96	13.04
Cr_2O_3	0.02	0.03	0.00	0.00	0.01	0.05	0.01	0.00	0.00	0.01	0.00	0.00	0.01	0.03	0.02
FeO_{tot}	11.33	12.05	12.82	13.04	12.31	11.88	12.22	11.59	12.18	13.17	13.21	12.54	11.47	11.76	11.14
MnO	0.10	0.12	0.17	0.16	0.13	0.13	0.13	0.11	0.12	0.14	0.18	0.11	0.10	0.09	0.10
NiO+ZnO	0.00	0.00	0.00	0.00	0.00	0.00	0.00	0.00	0.00	0.00	0.00	0.00	0.00	0.00	0.00
MgO	14.44	14.16	13.32	13.14	13.51	13.90	13.62	13.88	13.78	13.17	13.51	13.74	14.19	14.27	14.55
CaO	11.83	11.65	11.82	11.73	11.91	11.99	11.52	11.89	11.78	11.77	11.51	11.64	11.77	11.76	11.64
Na_2O	2.09	2.09	2.11	2.14	2.04	2.02	2.13	1.96	2.20	2.10	2.07	2.12	2.05	2.04	2.08
K_2O	1.49	1.44	1.41	1.39	1.49	1.62	1.63	1.69	1.33	1.38	1.42	1.43	1.54	1.49	1.54
F	0.00	0.00	0.00	0.00	0.00	0.00	0.00	0.00	0.00	0.00	0.00	0.00	0.00	0.00	0.00
Cl	0.00	0.00	0.00	0.00	0.00	0.00	0.00	0.00	0.00	0.00	0.00	0.00	0.00	0.00	0.00
Sum	98.04	98.05	98.00	98.00	98.02	98.06	98.06	98.07	98.01	98.01	98.02	98.01	98.06	98.07	98.06
Fe_2O_3	3.81	3.98	4.00	4.08	3.89	4.01	3.86	3.76	3.89	4.05	4.13	4.08	3.86	3.96	3.78
FeO	7.90	8.47	9.22	9.36	8.81	8.28	8.74	8.20	8.68	9.52	9.49	8.87	8.00	8.20	7.73
O=F,Cl	0.00	0.00	0.00	0.00	0.00	0.00	0.00	0.00	0.00	0.00	0.00	0.00	0.00	0.00	0.00
H_2O	1.56	1.53	1.60	1.58	1.59	1.53	1.55	1.54	1.60	1.57	1.56	1.57	1.54	1.52	1.55

(Continued)

TEO	99.99	99.99	99.99	99.99	99.99	99.99	99.99	99.99	99.99	99.99	99.99	99.99	99.99	99.98	99.99
Stage 3: AMFORM formula															
Mg(apfu)	3.174	3.122	2.946	2.908	2.982	3.071	3.007	3.058	3.036	2.917	2.994	3.035	3.121	3.143	3.194
Si	6.136	6.090	6.092	6.060	6.120	6.060	6.111	6.110	6.110	6.077	6.054	6.108	6.132	6.103	6.132
Al	0.366	0.366	0.404	0.430	0.408	0.364	0.418	0.423	0.405	0.401	0.386	0.384	0.389	0.361	0.396
Ti	0.254	0.257	0.230	0.230	0.240	0.265	0.257	0.259	0.235	0.238	0.233	0.239	0.264	0.265	0.256
Fe^{3+}	0.423	0.443	0.446	0.456	0.433	0.447	0.430	0.418	0.432	0.453	0.462	0.455	0.429	0.440	0.419
Fe^{2+}	0.974	1.048	1.144	1.163	1.091	1.026	1.083	1.014	1.073	1.183	1.180	1.098	0.987	1.013	0.952
Ca	0.075	0.100	0.070	0.073	0.062	0.098	0.040	0.068	0.064	0.084	0.111	0.073	0.063	0.098	0.069
Na	0.596	0.599	0.608	0.615	0.586	0.582	0.612	0.561	0.629	0.606	0.597	0.608	0.587	0.584	0.593
K	0.281	0.271	0.267	0.264	0.282	0.307	0.308	0.318	0.250	0.261	0.269	0.271	0.290	0.280	0.288
Stage 4: Calculated Mg, △NNO and △FMQ*															
Mg*	3.049	3.008	2.880	2.842	2.900	2.952	2.893	2.942	2.937	2.849	2.938	2.953	2.989	3.019	3.060
△NNO	1.002	0.935	0.724	0.662	0.757	0.844	0.746	0.827	0.818	0.675	0.821	0.845	0.903	0.952	1.020
△FMQ	1.702	1.635	1.424	1.362	1.457	1.544	1.446	1.527	1.518	1.375	1.521	1.545	1.603	1.652	1.720

Table S7*(Continued)*

Label	Amphibole Oxybarometer															
	SSB5-2 Line 035	SSB5-2 Line 036	SSB5-2 Line 037	SSB5-2 Line 038	SSB5-2 Line 039	SSB5-2 Line 040	SSB5-2 Line 041	SSB5-2 Line 042	SSB5-2 Line 043	SSB5-2 Line 044	SSB5-2 Line 045	SSB5-2 Line 046	SSB5-2 Line 047	SSB5-2 Line 048	SSB5-2 Line 049	SSB5-2 Line 050
Stage 1: Amphibole compositions																
SiO_2(wt%)	41.50	41.60	41.20	41.20	40.80	40.80	40.10	41.60	41.20	41.60	41.20	41.20	40.90	40.90	41.20	41.40
TiO_2	2.30	2.09	2.27	2.35	2.11	2.18	2.10	2.16	2.32	2.29	2.07	2.09	2.05	2.03	2.18	2.36
Al_2O_3	13.10	13.10	12.80	13.20	13.50	13.40	13.70	12.80	13.10	13.00	13.00	13.20	13.30	13.10	12.80	13.10
Cr_2O_3	0.03	0.03	0.00	0.01	0.00	0.00	0.02	0.03	0.10	0.09	0.00	0.03	0.00	0.00	0.01	0.00
FeO_{tot}	10.20	11.10	11.50	11.60	13.30	13.10	14.00	10.90	11.10	11.00	12.40	12.60	12.60	13.50	11.70	12.00
MnO	0.10	0.06	0.11	0.12	0.12	0.16	0.14	0.11	0.10	0.11	0.11	0.12	0.14	0.13	0.14	0.08
NiO+ZnO	0.00	0.00	0.00	0.00	0.00	0.00	0.00	0.00	0.00	0.00	0.00	0.00	0.00	0.00	0.00	0.00
MgO	14.70	14.70	14.20	14.10	13.30	13.10	12.60	14.60	14.40	14.60	13.90	13.80	13.50	13.00	14.20	14.30
CaO	11.60	11.80	11.70	11.70	11.60	11.70	11.60	11.60	11.60	11.60	11.70	11.70	11.80	11.40	11.80	11.80
Na_2O	2.07	2.17	2.06	2.03	2.07	2.15	2.04	2.15	2.08	2.19	2.07	2.12	2.01	2.10	2.07	2.13
K_2O	1.58	1.54	1.54	1.50	1.52	1.52	1.54	1.54	1.60	1.57	1.50	1.47	1.52	1.47	1.49	1.43
F	0.00	0.00	0.00	0.00	0.00	0.00	0.00	0.00	0.00	0.00	0.00	0.00	0.00	0.00	0.00	0.00
Cl	0.00	0.00	0.00	0.00	0.00	0.00	0.00	0.00	0.00	0.00	0.00	0.00	0.00	0.00	0.00	0.00
Stage 2: Initial total re-calculation																
SiO_2(wt%)	41.87	41.53	41.48	41.31	40.68	40.77	40.18	41.82	41.40	41.60	41.22	41.07	40.98	41.05	41.38	41.17
TiO_2	2.32	2.09	2.29	2.36	2.10	2.18	2.10	2.17	2.33	2.29	2.07	2.08	2.05	2.04	2.19	2.35
Al_2O_3	13.22	13.08	12.89	13.23	13.46	13.39	13.73	12.87	13.16	13.00	13.01	13.16	13.33	13.15	12.86	13.03
Cr_2O_3	0.03	0.03	0.00	0.01	0.00	0.00	0.02	0.03	0.10	0.09	0.00	0.03	0.00	0.00	0.01	0.00
FeO_{tot}	10.29	11.08	11.58	11.63	13.26	13.09	14.03	10.96	11.15	11.00	12.41	12.56	12.62	13.55	11.75	11.93
MnO	0.10	0.05	0.11	0.12	0.12	0.16	0.14	0.11	0.10	0.11	0.11	0.11	0.14	0.13	0.14	0.08
NiO+ZnO	0.00	0.00	0.00	0.00	0.00	0.00	0.00	0.00	0.00	0.00	0.00	0.00	0.00	0.00	0.00	0.00
MgO	14.83	14.67	14.30	14.14	13.26	13.09	12.62	14.68	14.47	14.60	13.91	13.76	13.53	13.05	14.26	14.22
CaO	11.70	11.78	11.78	11.73	11.57	11.69	11.62	11.66	11.66	11.60	11.71	11.66	11.82	11.44	11.85	11.74
Na_2O	2.09	2.17	2.07	2.04	2.06	2.15	2.04	2.16	2.09	2.19	2.07	2.11	2.01	2.11	2.08	2.12
K_2O	1.59	1.54	1.55	1.50	1.52	1.52	1.54	1.55	1.61	1.57	1.50	1.47	1.52	1.48	1.50	1.42
F	0.00	0.00	0.00	0.00	0.00	0.00	0.00	0.00	0.00	0.00	0.00	0.00	0.00	0.00	0.00	0.00
Cl	0.00	0.00	0.00	0.00	0.00	0.00	0.00	0.00	0.00	0.00	0.00	0.00	0.00	0.00	0.00	0.00
Sum	98.05	98.01	98.03	98.07	98.03	98.03	98.02	98.02	98.07	98.06	98.01	98.02	98.01	97.99	98.02	98.06
Fe_2O_3	3.73	3.95	3.96	3.81	4.21	4.03	4.38	3.81	3.81	3.68	4.10	4.06	4.10	4.24	3.94	3.92
FeO	6.93	7.53	8.01	8.20	9.47	9.46	10.08	7.53	7.72	7.69	8.72	8.91	8.94	9.74	8.20	8.41
O=F,Cl	0.00	0.00	0.00	0.00	0.00	0.00	0.00	0.00	0.00	0.00	0.00	0.00	0.00	0.00	0.00	0.00
H_2O	1.56	1.58	1.56	1.53	1.54	1.56	1.53	1.59	1.53	1.56	1.57	1.57	1.57	1.57	1.58	1.53

(Continued)																
TEO	99.99	99.99	99.99	99.99	99.99	99.99	99.99	99.99	99.98	99.99	99.99	99.99	99.99	99.99	99.99	99.99
Stage 3: AMFORM formula																
Mg(apfu)	3.243	3.221	3.146	3.112	2.940	2.902	2.812	3.219	3.181	3.206	3.070	3.040	2.990	2.892	3.142	3.136
Si	6.142	6.115	6.123	6.100	6.051	6.063	6.003	6.152	6.104	6.128	6.105	6.087	6.078	6.104	6.115	6.090
Al	0.427	0.384	0.366	0.403	0.410	0.410	0.420	0.383	0.392	0.385	0.375	0.386	0.407	0.409	0.354	0.361
Ti	0.256	0.231	0.254	0.262	0.235	0.244	0.236	0.240	0.259	0.254	0.231	0.232	0.229	0.228	0.243	0.261
Fe^{3+}	0.412	0.437	0.440	0.423	0.471	0.452	0.492	0.422	0.423	0.408	0.457	0.453	0.457	0.474	0.438	0.436
Fe^{2+}	0.851	0.927	0.989	1.013	1.178	1.176	1.260	0.926	0.952	0.947	1.080	1.104	1.109	1.211	1.014	1.040
Ca	0.043	0.070	0.072	0.086	0.093	0.066	0.101	0.046	0.072	0.055	0.084	0.085	0.088	0.054	0.086	0.104
Na	0.594	0.618	0.594	0.583	0.595	0.619	0.592	0.616	0.598	0.625	0.595	0.607	0.579	0.608	0.596	0.607
K	0.298	0.289	0.292	0.283	0.288	0.288	0.294	0.291	0.302	0.295	0.284	0.277	0.288	0.280	0.282	0.268
Stage 4: Calculated Mg, △NNO and △FMQ*																
Mg*	3.086	3.111	3.030	2.983	2.884	2.823	2.775	3.095	3.043	3.061	3.003	2.967	2.933	2.851	3.042	3.010
△NNO	1.063	1.104	0.972	0.895	0.732	0.631	0.553	1.078	0.993	1.023	0.927	0.868	0.811	0.678	0.992	0.938
△FMQ	1.763	1.804	1.672	1.595	1.432	1.331	1.253	1.778	1.693	1.723	1.627	1.568	1.511	1.378	1.692	1.638

Note: 1) Calculation of amphibole composition is based on AMFORM (*124*).

2) Oxygen fugacity based on the three equations of the amphibole oxybarometer: i) $Mg^{*} = Mg+Si/47-{}^{VI}Al/9-1.3\times{}^{VI}Ti+Fe^{3+}/3.7+Fe^{2+}/52-{}^{B}Ca/20-{}^{A}Na/28+{}^{A}K/9.5$; ii) $\Delta NNO = 1.644Mg^{*}-4.01$; iii) $\Delta FMQ = \Delta NNO+0.7$ (*125*).

3) Compositional data for amphibole from Jiaodong lamprophyres are from (*126*, *127*).

REFERENCES AND NOTES

1. H. E. Frimmel, Earth's continental crustal gold endowment. *Earth Planet. Sci. Lett.* **267**, 45–55 (2008).

2. R. L. Romer, U. Kroner, Paleozoic gold in the Appalachians and Variscides. *Ore Geol. Rev.* **92**, 475–505 (2018).

3. R. J. Goldfarb, C. Hart, G. Davis, D. Groves, East Asian gold: Deciphering the anomaly of Phanerozoic gold in Precambrian cratons. *Econ. Geol.* **102**, 341–345 (2007).

4. D. I. Groves, R. M. Vielreicher, R. J. Goldfarb, K. C. Condie, Controls on the heterogeneous distribution of mineral deposits through time. *Geol. Soc. Lond. Spec. Publ.* **248**, 71–101 (2005).

5. R. J. Goldfarb, D. I. Groves, S. Gardoll, Orogenic gold and geologic time: A global synthesis. *Ore Geol. Rev.* **18**, 1–75 (2001).

6. W. L. Griffin, S. Y. O'Reilly, J. C. Afonso, G. C. Begg, The composition and evolution of lithospheric mantle: A re-evaluation and its tectonic implications. *J. Petrol.* **50**, 1185–1204 (2009).

7. W. L. Griffin, G. C. Begg, S. Y. O'Reilly, Continental-root control on the genesis of magmatic ore deposits. *Nat. Geosci.* **6**, 905–910 (2013).

8. S. Tassara, A. D. Rooney, J. J. Ague, D. Guido, M. Reich, F. Barra, C. Navarrete, Osmium isotopes fingerprint mantle controls on the genesis of an epithermal gold province. *Geology* **50**, 1291–1295 (2022).

9. D. I. Groves, L. Zhang, M. Santosh, Subduction, mantle metasomatism, and gold: A dynamic and genetic conjunction. *GSA Bull.* **132**, 1419–1426 (2020).

10. C. G. Soder, J. Dunga, R. L. Romer, Continental subduction controls regional magma heterogeneity and distribution of porphyry deposits in post-collisional settings. *Geochim. Cosmochim. Acta* **375**, 217–228 (2024).

11. R. Zhu, W. Sun, The big mantle wedge and decratonic gold deposits. *Sci. China Earth Sci.* **64**, 1451–1462 (2021).

12. S. Ishimaru, S. Arai, H. Shukuno, Metal-saturated peridotite in the mantle wedge inferred from metal-bearing peridotite xenoliths from Avacha volcano, Kamchatka. *Earth Planet. Sci. Lett.* **284**, 352–360 (2009).

13. J. Wang, K. H. Hattori, R. Kilian, C. R. Stern, Metasomatism of sub-arc mantle peridotites below southernmost South America: Reduction of fO_2 by slab-melt. *Contrib. Mineral. Petrol.* **153**, 607–624 (2006).

14. F. E. Jenner, Cumulate causes for the low contents of sulfide-loving elements in the continental crust. *Nat. Geosci.* **10**, 524–529 (2017).

15. W. Sun, R. J. Arculus, V. S. Kamenetsky, R. A. Binns, Release of gold-bearing fluids in convergent margin magmas prompted by magnetite crystallization. *Nature* **431**, 975–978 (2004).

16. J. E. Mungall, J. J. Hanley, N. T. Arndt, A. Debecdelievre, Evidence from meimechites and other low-degree mantle melts for redox controls on mantle-crust fractionation of platinum-group elements. *Proc. Natl. Acad. Sci. U.S.A.* **103**, 12695–12700 (2006).

17. J. E. Mungall, Roasting the mantle: Slab melting and the genesis of major Au and Au-rich Cu deposits. *Geology* **30**, 915–918 (2002).

18. R. E. Botcharnikov, R. L. Linnen, M. Wilke, F. Holtz, P. J. Jugo, J. Berndt, High gold concentrations in sulphide-bearing magma under oxidizing conditions. *Nat. Geosci.* **4**, 112–115 (2011).

19. Y. Li, L. Feng, E. S. Kiseeva, Z. Gao, H. Guo, Z. Du, F. Wang, L. Shi, An essential role for sulfur in sulfide-silicate melt partitioning of gold and magmatic gold transport at subduction settings. *Earth Planet. Sci. Lett.* **528**, 115850 (2019).

20. C. Grondahl, Z. Zajacz, Magmatic controls on the genesis of porphyry Cu–Mo–Au deposits: The Bingham Canyon example. *Earth Planet. Sci. Lett.* **480**, 53–65 (2017).

21. C. Zhang, W. Sun, J. Wang, L. Zhang, S. Sun, K. Wu, Oxygen fugacity and porphyry mineralization: A zircon perspective of Dexing porphyry Cu deposit, China. *Geochim. Cosmochim. Acta* **206**, 343–363 (2017).

22. J. C. Alt, Sulfur isotopic profile through the oceanic crust: Sulfur mobility and seawater-crustal sulfur exchange during hydrothermal alteration. *Geology* **23**, 585–588 (1995).

23. J. C. Alt, E. M. Schwarzenbach, G. L. Früh-Green, W. C. Shanks, S. M. Bernasconi, C. J. Garrido, L. Crispini, L. Gaggero, J. A. Padrón-Navarta, C. Marchesi, The role of serpentinites in cycling of carbon and sulfur: Seafloor serpentinization and subduction metamorphism. *Lithos* **178**, 40–54 (2013).

24. K. A. Evans, The redox budget of subduction zones. *Earth Sci. Rev.* **113**, 11–32 (2012).

25. J. A. Padrón-Navarta, V. López Sánchez-Vizcaíno, M. D. Menzel, M. T. Gómez-Pugnaire, C. J. Garrido, Mantle wedge oxidation from deserpentinization modulated by sediment-derived fluids. *Nat. Geosci.* **16**, 268–275 (2023).

26. W. Li, Z. Yang, M. Chiaradia, Y. Lai, C. Yu, J. Zhang, Redox state of southern Tibetan upper mantle and ultrapotassic magmas. *Geology* **48**, 733–736 (2020).

27. G. S. Pokrovski, M. A. Kokh, D. Guillaume, A. Y. Borisova, P. Gisquet, J.-L. Hazemann, E. Lahera, W. Del Net, O. Proux, D. Testemale, V. Haigis, R. Jonchière, A. P. Seitsonen, G. Ferlat, R. Vuilleumier, A. M. Saitta, M.-C. Boiron, J. Dubessy, Sulfur radical species form gold deposits on Earth. *Proc. Natl. Acad. Sci. U.S.A.* **112**, 13484–13489 (2015).

28. D. E. Blanks, D. A. Holwell, M. L. Fiorentini, M. Moroni, A. Giuliani, S. Tassara, J. M. González-Jiménez, A. J. Boyce, E. Ferrari, Fluxing of mantle carbon as a physical agent for metallogenic fertilization of the crust. *Nat. Commun.* **11**, 4342 (2020).

29. G. N. Phillips, K. A. Evans, Role of CO_2 in the formation of gold deposits. *Nature* **429**, 860–863 (2004).

30. R. J. Goldfarb, D. I. Groves, Orogenic gold: Common or evolving fluid and metal sources through time. *Lithos* **233**, 2–26 (2015).

31. R. J. Goldfarb, R. D. Taylor, G. S. Collins, N. A. Goryachev, O. F. Orlandini, Phanerozoic continental growth and gold metallogeny of Asia. *Gondw. Res.* **25**, 48–102 (2014).

32. L. Krmíček, R. L. Romer, M. J. Timmerman, J. Ulrych, J. Glodny, A. Přichystal, M. Sudo, Long-lasting (65 Ma) regionally contrasting late- to post-orogenic Variscan mantle-derived potassic magmatism in the Bohemian Massif. *J. Petrol.* **61**, egaa072 (2020).

33. X. Wang, Z. Wang, H. Cheng, K. Zong, C. Y. Wang, L. Ma, Y.-C. Cai, S. Foley, Z. Hu, Gold endowment of the metasomatized lithospheric mantle for giant gold deposits: Insights from lamprophyre dykes. *Geochim. Cosmochim. Acta* **316**, 21–40 (2022).

34. E. Choi, M. L. Fiorentini, H. S. R. Hughes, A. Giuliani, Platinum-group element and Au geochemistry of late Archean to Proterozoic calc-alkaline and alkaline magmas in the Yilgarn Craton, Western Australia. *Lithos* **374–375**, 105716 (2020).

35. J. Deng, X. Liu, Q. Wang, Y. Dilek, Y. Liang, Isotopic characterization and petrogenetic modeling of early Cretaceous mafic diking—Lithospheric extension in the North China Craton, Eastern Asia. *GSA Bull.* **129**, 1379–1407 (2017).

36. N. M. Rock, D. I. Groves, Do lamprophyres carry gold as well as diamonds? *Nature* **332**, 253–255 (1988).

37. D. Müller, D. I. Groves, Indirect associations between lamprophyres and gold-copper deposits in *Potassic Igneous Rocks and Associated Gold-Copper Mineralization* (Springer Cham, 2019), pp. 279–306.

38. K.-F. Qiu, R. J. Goldfarb, J. Deng, H.-C. Yu, Z.-Y. Gou, Z.-J. Ding, Z.-K. Wang, D.-P. Li, Gold deposits of the Jiaodong Peninsula, Eastern China, in *Geology of the World's Major Gold Deposits and Provinces*, R. H. Sillitoe, R. J. Goldfarb, F. Robert, S. F. Simmons, Eds. (Society of Economic Geologists, 2020), vol. 23, pp. 753–773; https://doi.org/10.5382/SP.23.35.

39. J.-Y. Zhang, K.-F. Qiu, R. Yin, Z.-Y. Long, Y.-C. Feng, H.-C. Yu, Z.-Y. Gao, J. Deng, Lithospheric mantle as a metal storage reservoir for orogenic gold deposits in active continental margins: Evidence from Hg isotopes. *Geology* **52**, 423–428 (2024).

40. X. Geng, Y. Liu, X.-C. Wang, Z. Hu, L. Zhou, S. Gao, The role of Earth's deep volatile cycling in the generation of intracontinental high-Mg andesites: Implication for lithospheric thinning beneath the North China Craton. *J. Geophys. Res. Solid Earth* **124**, 1305–1323 (2019).

41. Q.-K. Xia, J. Liu, S.-C. Liu, I. Kovacs, M. Feng, L. Dang, High water content in Mesozoic primitive basalts of the North China Craton and implications on the destruction of cratonic mantle lithosphere. *Earth Planet. Sci. Lett.* **361**, 85–97 (2013).

42. J. Deng, L.-Q. Yang, D. I. Groves, L. Zhang, K.-F. Qiu, Q.-F. Wang, An integrated mineral system model for the gold deposits of the giant Jiaodong province, Eastern China. *Earth Sci. Rev.* **208**, 103274 (2020).

43. X. Geng, S. F. Foley, Y. Liu, Z. Wang, Z. Hu, L. Zhou, Thermal-chemical conditions of the North China Mesozoic lithospheric mantle and implication for the lithospheric thinning of cratons. *Earth Planet. Sci. Lett.* **516**, 1–11 (2019).

44. R. J. Goldfarb, M. Santosh, The dilemma of the Jiaodong gold deposits: Are they unique? *Geosci. Front.* **5**, 139–153 (2014).

45. K.-F. Qiu, J. Deng, C. Laflamme, Z.-Y. Long, R.-Q. Wan, F. Moynier, H.-C. Yu, J.-Y. Zhang, Z.-J. Ding, R. Goldfarb, Giant Mesozoic gold ores derived from subducted oceanic slab and overlying sediments. *Geochim. Cosmochim. Acta* **343**, 133–141 (2023).

46. Q. Wang, X. Liu, R. Yin, W. Weng, H. Zhao, L. Yang, D. Zhai, D. Li, Y. Ma, D. I. Groves, J. Deng, Metasomatized mantle sources for orogenic gold deposits hosted in high-grade metamorphic rocks: Evidence from Hg isotopes. *Geology* **52**, 115–119 (2023).

47. T. Kusky, B. F. Windley, M.-G. Zhai, Tectonic evolution of the North China Block: From orogen to craton to orogen. *Geol. Soc. Lond. Spec. Publ.* **280**, 1–34 (2007).

48. Z. Wang, Z. Xu, H. Cheng, Y. Zou, J. Guo, Y. Liu, J. Yang, K. Zong, L. Xiong, Z. Hu, Precambrian metamorphic crustal basement cannot provide much gold to form giant gold deposits in the Jiaodong Peninsula, China. *Precambrian Res.* **354**, 106045 (2021).

49. J.-H. Yang, F.-Y. Wu, S. A. Wilde, E. Belousova, W. L. Griffin, Mesozoic decratonization of the North China block. *Geology* **36**, 467–470 (2008).

50. L. Ma, S.-Y. Jiang, A. W. Hofmann, B.-Z. Dai, M.-L. Hou, K.-D. Zhao, L.-H. Chen, J.-W. Li, Y.-H. Jiang, Lithospheric and asthenospheric sources of lamprophyres in the Jiaodong Peninsula: A consequence of rapid lithospheric thinning beneath the North China Craton? *Geochim. Cosmochim. Acta* **124**, 250–271 (2014).

51. X. Wang, Z. Wang, W. Zhang, L. Ma, W. Chen, Y.-C. Cai, S. Foley, C. Y. Wang, J. Li, J. Deng, Y. Feng, K. Zong, Z. Hu, Y. Liu, Sulfur isotopes of lamprophyres and implications for the control of metasomatized lithospheric mantle on the giant Jiaodong gold deposits, Eastern China. *GSA Bull.* **136**, 340–3418 (2024).

52. K.-F. Qiu, J. Deng, S.-X. Sai, H.-C. Yu, M. T. Tamer, Z.-J. Ding, X.-F. Yu, R., Goldfarb, low-temperature thermochronology for defining the tectonic controls on heterogeneous gold endowment across the Jiaodong Peninsula, Eastern China. *Tectonics* **42**, e2022TC007669 (2023).

53. H. R. Marschall, V. D. Wanless, N. Shimizu, P. A. E. Pogge von Strandmann, T. Elliott, B. D. Monteleone, The boron and lithium isotopic composition of mid-ocean ridge basalts and the mantle. *Geochim. Cosmochim. Acta* **207**, 102–138 (2017).

54. J. E. Saunders, N. J. Pearson, S. Y. O'Reilly, W. L. Griffin, Sulfide metasomatism and the mobility of gold in the lithospheric mantle. *Chem. Geol.* **410**, 149–161 (2015).

55. Z. Wang, H. Cheng, K. Zong, X. Geng, Y. Liu, J. Yang, F. Wu, H. Becker, S. Foley, C. Y. Wang, Metasomatized lithospheric mantle for Mesozoic giant gold deposits in the North China craton. *Geology* **48**, 169–173 (2020).

56. H. Palme, H. St. C. O'Neill, Cosmochemical Estimates of Mantle Composition, in *Treatise on Geochemi*stry, H. D. Holland, K. K. Turekian, Eds. (Pergamon, Oxford, 2007), pp. 1–38; https://www.sciencedirect.com/science/article/pii/B0080437516021770.

57. J. E. Saunders, N. J. Pearson, S. Y. O'Reilly, W. L. Griffin, Gold in the mantle: A global assessment of abundance and redistribution processes. *Lithos* **322**, 376–391 (2018).

58. D. A. Holwell, M. Fiorentini, I. McDonald, Y. Lu, A. Giuliani, D. J. Smith, M. Keith, M. Locmelis, A metasomatized lithospheric mantle control on the metallogenic signature of post-subduction magmatism. *Nat. Commun.* **10**, 3511 (2019).

59. J. Hermann, C. J. Spandler, Sediment melts at sub-arc depths: An experimental study. *J. Petrol.* **49**, 717–740 (2008).

60. S. Y. O'Reilly, W. L. Griffin, Mantle metasomatism in *Metasomatism and the chemical transformation of rock* (Springer Berlin, 2013) pp. 471–533.

61. F. P. Bierlein, S. Pisarevsky, Plume-related oceanic plateaus as a potential source of gold mineralization. *Econ. Geol.* **103**, 425–430 (2008).

62. D. I. Groves, K. C. Condie, R. J. Goldfarb, J. M. A. Hronsky, R. M. Vielreicher, Secular changes in global tectonic processes and their influence on the temporal distribution of gold-bearing mineral deposits. *Econ. Geol.* **100**, 203–224 (2005).

63. Y. Weiss, C. Class, S. L. Goldstein, T. Hanyu, Key new pieces of the HIMU puzzle from olivines and diamond inclusions. *Nature* **537**, 666–670 (2016).

64. L. Sauzéat, R. L. Rudnick, C. Chauvel, M. Garçon, M. Tang, New perspectives on the Li isotopic composition of the upper continental crust and its weathering signature. *Earth Planet. Sci. Lett.* **428**, 181–192 (2015).

65. M. Tang, R. L. Rudnick, C. Chauvel, Sedimentary input to the source of Lesser Antilles lavas: A Li perspective. *Geochim. Cosmochim. Acta* **144**, 43–58 (2014).

66. H. R. Marschall, P. A. E. Pogge von Strandmann, H.-M. Seitz, T. Elliott, Y. Niu, The lithium isotopic composition of orogenic eclogites and deep subducted slabs. *Earth Planet. Sci. Lett.* **262**, 563–580 (2007).

67. D. Prelević, D. E. Jacob, S. F. Foley, Recycling plus: A new recipe for the formation of Alpine–Himalayan orogenic mantle lithosphere. *Earth Planet. Sci. Lett.* **362**, 187–197 (2013).

68. C. G. Soder, R. L. Romer, Post-collisional potassic–ultrapotassic magmatism of the Variscan orogen: Implications for mantle metasomatism during continental subduction. *J. Petrol.* **59**, 1007–1034 (2018).

69. K. M. Abdelfadil, R. L. Romer, J. Glodny, Mantle wedge metasomatism revealed by Li isotopes in orogenic lamprophyres. *Lithos* **196–197**, 14–26 (2014).

70. L. Dong, Z. Yang, Y. Liu, M. Song, Possible source of Au in the Jiaodong area from lower crustal sulfide cumulates: Evidence from oxygen states and chalcophile elements contents of mesozoic magmatic suites. *Ore Geol. Rev.* **153**, 105268 (2023).

71. L. D. Benton, J. G. Ryan, I. P. Savov, Lithium abundance and isotope systematics of forearc serpentinites, Conical Seamount, Mariana forearc: Insights into the mechanics of slab-mantle exchange during subduction. *Geochem. Geophys. Geosyst.* **5**, Q08J12 (2004).

72. T. Elliott, A. Thomas, A. Jeffcoate, Y. Niu, Lithium isotope evidence for subduction-enriched mantle in the source of mid-ocean-ridge basalts. *Nature* **443**, 565–568 (2006).

73. A. Giuliani, V. S. Kamenetsky, D. Phillips, M. A. Kendrick, B. A. Wyatt, K. Goemann, Nature of alkali-carbonate fluids in the sub-continental lithospheric mantle. *Geology* **40**, 967–970 (2012).

74. Z.-X. Wang, S.-A. Liu, S. Li, D. Liu, J. Liu, Linking deep CO_2 outgassing to cratonic destruction. *Natl. Sci. Rev.* **9**, nwac001 (2022).

75. D. Liu, Z. Zhao, D.-C. Zhu, Y. Niu, E. Widom, F.-Z. Teng, D. J. DePaolo, S. Ke, J.-F. Xu, Q. Wang, X. Mo, Identifying mantle carbonatite metasomatism through Os–Sr–Mg isotopes in Tibetan ultrapotassic rocks. *Earth Planet. Sci. Lett.* **430**, 458–469 (2015).

76. D.-B. Tan, Y. Xiao, L.-Q. Dai, H. Sun, Y. Wang, H.-O. Gu, Differentiation between carbonate and silicate metasomatism based on lithium isotopic compositions of alkali basalts. *Geology* **50**, 1150–1155 (2022).

77. D. Prelević, C. Akal, S. F. Foley, R. L. Romer, A. Stracke, P. Van Den Bogaard, Ultrapotassic mafic rocks as geochemical proxies for post-collisional dynamics of orogenic lithospheric mantle: The case of Southwestern Anatolia, Turkey. *J. Petrol.* **53**, 1019–1055 (2012).

78. D. Prelević, C. Akal, R. L. Romer, R. Mertz-Kraus, C. Helvacı, Magmatic response to slab tearing: Constraints from the Afyon alkaline volcanic complex, Western Turkey. *J. Petrol.* **56**, 527–562 (2015).

79. S. Graham, D. Lambert, S. Shee, The petrogenesis of carbonatite, melnoite and kimberlite from the Eastern Goldfields province, Yilgarn Craton. *Lithos* **76**, 519–533 (2004).

80. J. M. McArthur, R. J. Howarth, G. A. Shields, Y. Zhou, Strontium isotope stratigraphy, in *Geologic Time Scale 2020*, F. M. Gradstein, J. G. Ogg, M. D. Schmitz, G. M. Ogg, Eds. (Elsevier, 2020), pp. 211–238.

81. Z.-Y. Zhu, T. Yang, X.-K. Zhu, Achieving rapid analysis of Li isotopes in high-matrix and low-Li samples with MC-ICP-MS: New developments in sample preparation and mass bias behavior of Li in ICPMS. *J. Anal. At. Spectrom* **34**, 1503–1513 (2019).

82. G. D. Flesch, A. R. Anderson, H. J. Svec, A secondary isotopic standard for $^{6}Li/^{7}Li$ determinations. *Int. J. Mass Spectrom.* **12**, 265–272 (1973).

83. S. S. Sun, W. F. McDonough, Chemical and isotopic systematics of oceanic basalts: Implications for mantle composition and processes. *Geol. Soc. Lond. Spec. Publ.* **42**, 313–345 (1989).

84. L. Hong, Y. Xu, L. Zhang, Z. Liu, X. Xia, Y. Kuang, Oxidized late mesozoic subcontinental lithospheric mantle beneath the eastern North China Craton: A clue to understanding cratonic destruction. *Gondw. Res.* **81**, 230–239 (2020).

85. G. Zhao, M. Sun, S. A. Wilde, L. Sanzhong, Late Archean to Paleoproterozoic evolution of the North China Craton: Key issues revisited. *Precambrian Res.* **136**, 177–202 (2005).

86. T. M. Kusky, A. Polat, B. F. Windley, K. C. Burke, J. F. Dewey, W. S. F. Kidd, S. Maruyama, J. P. Wang, H. Deng, Z. S. Wang, C. Wang, D. Fu, X. W. Li, H. T. Peng, Insights into the tectonic evolution of the North China Craton through comparative tectonic analysis: A record of outward growth of Precambrian continents. *Earth. Sci. Rev.* **162**, 387–432 (2016).

87. R.-X. Zhu, J.-H. Yang, F.-Y. Wu, Timing of destruction of the North China Craton. *Lithos* **149**, 51–60 (2012).

88. B. F. Windley, D. Alexeiev, W. Xiao, A. Kröner, G. Badarch, Tectonic models for accretion of the Central Asian orogenic belt. *J. Geol. Soc. London* **164**, 31–47 (2007).

89. Y.-F. Zheng, Z.-F. Zhao, R.-X. Chen, "Ultrahigh-pressure metamorphic rocks in the Dabie–Sulu orogenic belt: Compositional inheritance and metamorphic modification" in *HP–UHP Metamorphism and Tectonic Evolution of Orogenic Belts*, L. Zhang, Z. Zhang, H.-P. Schertl, C. Wei, Eds. (Geological Society of London, 2019), vol. 474, p. 0; https://doi.org/10.1144/SP474.9.

90. H.-Y. Li, X.-L. Huang, Constraints on the paleogeographic evolution of the North China Craton during the late Triassic–Jurassic. *J. Asian Earth Sci.* **70-71**, 308–320 (2013).

91. Z. Wu, C. Lu, L. Qiu, H. Zhao, H. Wang, W. Tan, M. Zhong, New detrital zircon geochronological results from the Meso-Neoproterozoic sandstones in the southern-eastern Liaoning region, North China Craton, and their paleogeographic implications. *Precambrian Res.* **381**, 106847 (2022).

92. D.-B. Yang, H.-T. Yang, J.-P. Shi, W.-L. Xu, F. Wang, Sedimentary response to the paleogeographic and tectonic evolution of the southern North China Craton during the late paleozoic and mesozoic. *Gondw. Res.* **49**, 278–295 (2017).

93. T. M. Kusky, J. Li, Paleoproterozoic tectonic evolution of the North China Craton. *J. Asian Earth Sci.* **22**, 383–397 (2003).

94. Y. Wang, L. Zhou, S. Liu, J. Li, T. Yang, Post-cratonization deformation processes and tectonic evolution of the North China Craton. *Earth Sci. Rev.* **177**, 320–365 (2018).

95. H.-F. Zhang, M. Sun, X.-H. Zhou, W.-M. Fan, M.-G. Zhai, J.-F. Yin, Mesozoic lithosphere destruction beneath the North China Craton: Evidence from major-, trace-element and Sr–Nd–Pb isotope studies of Fangcheng basalts. *Contrib. Mineral. Petrol.* **144**, 241–254 (2002).

96. Q.-L. Yang, Z.-F. Zhao, Y.-F. Zheng, Modification of subcontinental lithospheric mantle above continental subduction zone: Constraints from geochemistry of Mesozoic gabbroic rocks in Southeastern North China. *Lithos* **146–147**, 164–182 (2012).

97. X. Wang, Z. Wang, H. Cheng, S. Foley, L. Xiong, Z. Hu, Early cretaceous lamprophyre dyke swarms in Jiaodong Peninsula, eastern North China Craton, and implications for mantle metasomatism related to subduction. *Lithos* **368–369**, 105593 (2020).

98. F.-Y. Wu, J.-H. Yang, Y.-G. Xu, S. A. Wilde, R. J. Walker, Destruction of the North China Craton in the Mesozoic. *Annu. Rev. Earth Planet. Sci.* **47**, 173–195 (2019).

99. S.-G. Li, W. Yang, S. Ke, X. Meng, H. Tian, L. Xu, Y. He, J. Huang, X.-C. Wang, Q. Xia, W. Sun, X. Yang, Z.-Y. Ren, H. Wei, Y. Liu, F. Meng, J. Yan, Deep carbon cycles constrained by a large-scale mantle Mg isotope anomaly in Eastern China. *Natl. Sci. Rev.* **4**, 111–120 (2017).

100. Y. Liang, X. Liu, C. Qin, Y. Li, J. Chen, J. Jiang, Petrogenesis of early Cretaceous mafic dikes in southeastern Jiaolai Basin, Jiaodong Peninsula, China. *Intl. Geol. Rev.* **59**, 131–150 (2017).

101. X. Liu, J. Deng, Y. Liang, Q. Wang, G. Li, Y. Ma, L. Xu, Y. Lu, Geochemical, mineralogical and chronological studies of mafic-intermediate dykes in the Jiaodong Peninsula:

Implications for late Mesozoic mantle source metasomatism and lithospheric thinning of the Eastern North China Craton. *Intl. Geol. Rev.* **62**, 2239–2260 (2020).

102. A. R. Woolley, S. C. Bergman, A. D. Edgar, M. J. Le Bas, R. H. Mitchell, N. M. Rock, B. H. Scott Smith, Classification of lamprophyres, lamproites, kimberlites, and the kalsilitic, melilitic, and leucitic rocks. *Can. Mineral.* **34**, 175–186 (1996).

103. A. Peccerillo, S. R. Taylor, Geochemistry of eocene calc-alkaline volcanic rocks from the Kastamonu area, Northern Turkey. *Contr. Mineral. Petrol.* **58**, 63–81 (1976).

104. S. Turner, N. Arnaud, J. Liu, N. Rogers, C. Hawkesworth, N. Harris, S. V. Kelley, P. Van Calsteren, W. Deng, Post-collision, shoshonitic volcanism on the Tibetan Plateau: Implications for convective thinning of the lithosphere and the source of ocean island basalts. *J. Petrol.* **37**, 45–71 (1996).

105. N. M. S. Rock, The nature and origin of lamprophyres: An overview. *Geol. Soc. Lond. Spec. Publ.* **30**, 191–226 (1987).

106. S. Duggen, K. Hoernle, P. van den Bogaard, D. Garbe-Schönberg, Post-collisional transition from subduction- to intraplate-type magmatism in the westernmost Mediterranean: Evidence for continental-edge delamination of subcontinental lithosphere. *J. Petrol.* **46**, 1155–1201 (2005).

107. D. A. Ionov, W. L. Griffin, S. Y. O'Reilly, Volatile-bearing minerals and lithophile trace elements in the upper mantle. *Chem. Geol.* **141**, 153–184 (1997).

108. T. Furman, D. Graham, Erosion of lithospheric mantle beneath the East African Rift system: Geochemical evidence from the Kivu volcanic province. *Lithos* **24**, 237–262 (1999).

109. R. E. Ernst, S. M. Jowitt, Large Igneous Provinces (LIPs) and metallogeny, in *Tectonics, Metallogeny, and Discovery: The North American Cordillera and Similar Accretionary Settings*, M. Colpron, T. Bissig, B. G. Rusk, J. F. H. Thompson, Eds. (Society of Economic Geologists, 2013), vol. 17, p. 0; https://doi.org/10.5382/SP.17.02.

110. P. B. Tomascak, F. Tera, R. T. Helz, R. J. Walker, The absence of lithium isotope fractionation during basalt differentiation: New measurements by multicollector sector ICP-MS. *Geochim. Cosmochim. Acta* **63**, 907–910 (1999).

111. L. H. Chan, J. M. Edmond, G. Thompson, K. Gillis, Lithium isotopic composition of submarine basalts: Implications for the lithium cycle in the oceans. *Earth Planet. Sci. Lett.* **108**, 151–160 (1992).

112. R. L. Romer, A. Meixner, H.-J. Förster, Lithium and boron in late-orogenic granites–Isotopic fingerprints for the source of crustal melts? *Geochim. Cosmochim. Acta* **131**, 98–114 (2014).

113. R. L. Rudnick, P. B. Tomascak, H. B. Njo, L. R. Gardner, Extreme lithium isotopic fractionation during continental weathering revealed in saprolites from South Carolina. *Chem. Geol.* **212**, 45–57 (2004).

114. W. Fang, L.-Q. Dai, Y.-F. Zheng, Z.-F. Zhao, Basalt Mo isotope evidence for crustal recycling in continental subduction zone. *Geochim. Cosmochim. Acta* **334**, 273–292 (2022).

115. J. Deng, Q.-F. Wang, L. Zhang, S.-C. Xue, X.-F. Liu, L. Yang, L.-Q. Yang, K.-F. Qiu, Y.-Y. Liang, Metallogenetic model of Jiaodong-type gold deposits, Eastern China. *Sci. China Earth Sci.* **66**, 2287–2310 (2023).

116. S. Penniston-Dorland, X.-M. Liu, R. L. Rudnick, Lithium isotope geochemistry. *Rev. Mineral. Geochem.* **82**, 165–217 (2017).

117. X.-J. Wang, L.-H. Chen, A. W. Hofmann, T. Hanyu, H. Kawabata, Y. Zhong, L.-W. Xie, J.-H. Shi, T. Miyazaki, Y. Hirahara, Recycled ancient ghost carbonate in the Pitcairn mantle plume. *Proc. Natl. Acad. Sci. U.S.A.* **115**, 8682–8687 (2018).

118. M. C. Johnson, T. Plank, Dehydration and melting experiments constrain the fate of subducted sediments. *Geochem. Geophys. Geosyst.* **1**, 1007 (2000).

119. Y.-R. Qu, S.-A. Liu, H. Wu, M.-L. Li, H.-C. Tian, Tracing carbonate dissolution in subducting sediments by zinc and magnesium isotopes. *Geochim. Cosmochim. Acta* **319**, 56–72 (2022).

120. V. J. M. Salters, A. Stracke, Composition of the depleted mantle. *Geochem. Geophys. Geosyst.* **5**, Q05B07 (2004).

121. S. Labanieh, C. Chauvel, A. Germa, X. Quidelleur, E. Lewin, Isotopic hyperbolas constrain sources and processes under the lesser antilles arc. *Earth Planet. Sci. Lett.* **298**, 35–46 (2010).

122. T. Plank, C. H. Langmuir, The chemical composition of subducting sediment and its consequences for the crust and mantle. *Chem. Geol.* **145**, 325–394 (1998).

123. C. Ma, C. Ehlers, C. Xu, Z. Li, K. Yang, The roots of the dabieshan ultrahigh-pressure metamorphic terrane: Constraints from geochemistry and Nd–Sr isotope systematics. *Precambrian Res.* **102**, 279–301 (2000).

124. F. Ridolfi, A. Zanetti, A. Renzulli, D. Perugini, F. Holtz, R. Oberti, AMFORM, a new mass-based model for the calculation of the unit formula of amphiboles from electron microprobe analyses. *Am. Mineral.* **103**, 1112–1125 (2018).

125. F. Ridolfi, A. Renzulli, M. Puerini, Stability and chemical equilibrium of amphibole in calc-alkaline magmas: An overview, new thermobarometric formulations and application to subduction-related volcanoes. *Contrib. Mineral. Petrol.* **160**, 45–66 (2010).

126. X. Liu, "Deep magmatic process of the Early Cretaceous gold metallogenic system in the eastern North China Craton: Evidence from in-situ geochemical analyses of minerals and melt inclusions from mantle xenoliths and intermediate-basic dykes," thesis, China Univ. of Geoscience (2022).

127. Y.-Y. Liang, "Petrogenesis of the Early Cretaceous Mafic Dikes and Metallogenic Dynamics in Jiaodong Peninsula," thesis, China Univ. of Geosciences (Beijing) (2017).
